# Recent Advances in Synthetic Quinoline-Based Compounds in Anticancer Drug Design

**DOI:** 10.3390/molecules31214000

**Published:** 2026-11-06

**Authors:** Łukasz Balewski, Chintankumar Padariya, Anita Kornicka

**Affiliations:** Department of Chemical Technology of Drugs, Faculty of Pharmacy, Medical University of Gdańsk, 80-416 Gdańsk, Poland; chintankumar.padariya@gumed.edu.pl (C.P.); anita.kornicka@gumed.edu.pl (A.K.)

**Keywords:** quinoline derivatives, benzo[*b*]pyridine derivatives, 2,3-benzopyridine derivatives, 1-azanaphthalene derivatives, anticancer activities, antiproliferative properties, biological properties, pharmacological activities, metal complexes

## Abstract

Compounds based on the quinoline (benzo[*b*]pyridine, 2,3-benzopyridine, or 1-azanaphthalene) core demonstrate extensive biological functionality. In recent years, scientific interest has converged on their antitumor properties, where they trigger antiproliferative responses through a variety of mechanisms, including the targeting of distinct signaling cascades. To overcome the limitations of high-specificity targeted therapies such as tumor resistance, recent research has shifted toward multi-target therapeutic strategies. The lack of strict drug specificity may be an advantage, offering improved pharmacodynamics. Compared to highly specific therapies, this multi-target strategy potentially may reduce the risks of cancer resistance. This review provides a comprehensive analysis of recent studies on the most critical targets for anticancer quinoline-based derivatives, detailing their mechanisms of inhibition or activation across various proteins, enzymes, and signaling pathways. These targets span receptor tyrosine kinases as well as cell-surface receptor complexes and integrin signaling modules. Inside the cell, quinolines modulate intracellular signaling and nuclear transcription factors alongside epigenetic factors. Furthermore, these compounds dictate cell cycle regulation and mitotic arrest. They also influence DNA integrity and post-translational modifications. Finally, quinolines address tumor microenvironment adaptation and efflux-mediated resistance. A wide array of anticancer activities indicates that quinolines may inhibit tubulin polymerization and induce apoptosis via caspase, or ROS-mediated pathways. Finally, due to the presence of aromatic nitrogen atoms, the quinoline derivatives constitute appropriate ligands that may be employed in the development of metal complexes. It has been demonstrated that such compounds, including 8-hydroxyquinoline-containg ligands, possess exceptional coordination abilities and transition metal recognizing properties. Various quinoline chelates containing calcium, nickel, zinc, ruthenium, hafnium, or silver possess improved anticancer properties which place them in a leading position for the development of effective chemotherapeutic agents. Given the vast amount of literature, this review summarizing the recent developments on synthetic quinoline-containing compounds and their metal complexes with proven anticancer properties is restricted to the short period with an emphasis on the 2025–2026 literature, while including essential background from earlier recent work (2022–2024).

## 1. Introduction

Quinoline is a nitrogen-containing carbocyclic system, also known as benzo[*b*]pyridine, 2,3-benzopyridine, or 1-azanaphthalene, which consists of the fusion of the aromatic ring of benzene and the heteroaromatic ring of pyridine (Figure 1). Structurally related to quinoline are its reduced forms: 1,2-dihydroquinoline, 1,2,3,4-tetrahydroquinoline, and decahydroquinoline which contain two, four, or eight hydrogen atoms added. These derivatives can be readily prepared by direct reduction of the corresponding quinoline-based compounds. Both quinoline derivatives and its reduced forms constitute valuable building blocks for further modifications of synthetic and natural quinoline-containing products with potential biological properties (Figure 1).

Quinolin’s skeleton is the structural component of a large group of compounds called alkaloids, found primarily in flowering plants across several families, including *Rutaceae*, *Annonaceae*, *Moraceae*, and *Rubiaceae* [1,2]. Many quinoline-based compounds are found in thousands of genera in these plant families, most of which have broad pharmacological properties, including antimicrobial, antiparasitic, anticancer, or cardiovascular activities [3,4,5,6]. Among the natural alkaloids bearing quinoline skeleton, quinine and quinidine are the most famous (Figure 1). Quinine comes from the *Cinchona* tree’s bark (*Cinchona officinalis*, *Rubiaceae*)—native to the Andes Mountains [7,8]. There has been a long tradition of using these naturally occurring compounds for antiparasitic purposes. Historically, *Cinchona* bark extracts have been used to combat malaria. Another example of quinoline-bearing alkaloid is shown in Figure 1 (4*S*)-4-ethyl-4-hydroxy-1*H*-pyrano[3′,4′:6,7]indolizino[1,2-*b*]quinoline-3,14(4*H*,12*H*)-dione—known under the brand name—camptothecin. This compound was isolated through systematic screening of natural sources for anticancer treatment from the bark and stem of Chinese tree *Camptotheca acuminata* (*Nyssaceae*) [9]. Apparently in vitro, both cytotoxic, DNA intercalating, and antiplasmodial properties have been shown by natural quinoline-based compound—cryptolepine (Figure 1) isolated from *Cryptolepis sanguinolenta* (*Periplocaceae*) [10,11].

In this regard, compounds containing a quinoline ring have been extensively studied in consideration of their biological properties. It was found that they exhibited potent and diverse therapeutic activities. Therefore, the quinoline ring is foreseen as a promising skeleton or privileged framework for the design of novel biologically active compounds [12]. Quinoline often constitutes a pharmacophore element responsible for the desired pharmacological activity and high affinity for a biological target [13,14]. This planar ring is rich in electrons and may interact with other molecules or chemical groups with electron-accepting properties resulting in the formation of charge-transfer complexes, whose stabilization results from electrostatic forces. Furthermore, compounds containing quinoline in their structure—due to the heteroatom’s presence—form stable dipoles, which can orient themselves in the electrostatic field of the binding site through dipole–dipole or ion–dipole interactions [15,16] The planar heteroaromatic quinoline ring can interact with biological targets through intercalation, inserted into base pairs in the DNA/RNA structure. Furthermore, the conjugated aromatic system of quinoline in the active center of the protein allows for hydrophobic and π–π interactions, which involve an electron cloud overlapping with aromatic amino acid residues (phenylalanine, tyrosine, and tryptophan) [17,18]. The presence of the quinoline ring increases the hydrophobicity of these molecules, thereby promoting more favorable interactions with hydrophobic binding pockets in enzymes and receptors. The quinoline nitrogen atom possesses a lone pair of electrons and acts as an acceptor of hydrogen bonds with hydroxyl or amino groups. Moreover, quinoline derivatives can also enter cation-π interactions, in which the delocalized electrons of the heteroaromatic ring can attract the positively charged amino groups of the prototonated amino acids side chains, i.e., lysine, arginine [19].

With all this in mind, quinoline occupies a prominent place in contemporary medicinal chemistry, and a wide range of drugs are based on the 2,3-benzopyridine scaffold. These include registered agents used in the treatment of malaria, cancers, bacterial and viral infections, cardiovascular diseases, or inflammations. For example, quinine became the framework of commonly used antimalarial agents [20,21]—chloroquine [22] and its analogs: amodiaquine [23], mefloquine [24], piperaquine [25], primaquine [26] (Figure 2).

Quinoline moiety is also present in antiretroviral drug saquinavir (Figure 3) [27], NS3/4A protease inhibitor—simeprevir (Figure 3) [28]—indicated against chronic HCV infections, and antituberculostatic agent bedaquiline [29] (Figure 3).

Hipercholesterolemia associated with high levels of trigycerides can be treated with a 2,3,4-trisubstituted quinoline derivative—pitavastatin [30]—a third-generation statin with HMG-CoA reductase inhibitory activity. This compound was approved by the US Food and Drug Administration (FDA) in 2009 in the form of calcium salt, under the trade name *Livalo* (Figure 4).

Quinoline also constitutes the skeleton of drugs used for pulmonary disorders such as chronic obstructive pulmonary disease, asthma, and bronchoconstriction. Examples are a long-acting β_2_-adrenoceptor agonist—8-hydroxyquinolin-2(1*H*)-one derivative—indacaterol (Figure 4) [31]—developed by *Novartis* and leukotriene receptor antagonist (LTRA)—montelukast (Figure 4) [32]—approved for clinical use in 1998 (USA) under the trade name *Singulair* (*Merck*).

It should be emphasized that numerous quinoline-incorporating compounds in recent years have been in clinical trials, with special attention to potential antiparasitic agents. A promising candidate for the next-generation antimalarial drug is ferroquine (SSR97193) and its derivatives [33,34].

Finally, quinoline derivatives are well known for their potent antiproliferative properties, particularly through their inhibition of VEGFR-2 signaling paths. Hydrophobic and π–π stacking interactions occur within the VEGFR-2 receptor binding pocket due to the aromatic ring. Hydrogen bonds may be formed by the quinoline nitrogen atom, which allow specific interactions with key residues in the ATP-binding hinge region [35]. Consequently, several approved anticancer drugs that predominantly act via the inhibition of VEGFR-mediated signaling pathways incorporate the quinoline scaffold.

Quinoline core is a major structural component of tivozanib [36,37,38,39], lenvatinib [40,41,42,43,44,45,46,47,48,49,50,51], and cabozantinib [52,53,54]—multi-targeted tyrosine kinase blocking agents—approved for the treatment of several cancers such as metastatic medullary thyroid cancer (MTC), differentiated thyroid cancer (DTC), advanced renal cell carcinoma (RCC), hepatoblastoma, and neuroendocrine tumors (NETs) (Figure 5). Recently, a potent oral Src/Abl tyrosine kinase inhibitor—bosutinib—was developed by *Pfizer* company for the treatment of chronic myeloid leukemia (CML) (Figure 5) [55,56].

On the other hand, irinotecan (Figure 5) [57] belongs to the class of plant alkaloid-derived chemotherapeutics, a semi-synthetic derivative of camptothecin that specifically targets topoisomerase I. The blockade of this nuclear enzyme prevents cancer cells from replicating and repairing their DNA, ultimately causing the cell death.

Recently, the pharmacological significance of the quinoline scaffold has triggered a substantial rise in the literature. Driven by the diverse potent biological profiles of quinolines, particularly their anticancer activities and recently published review articles on quinoline-based compounds [13,58,59,60,61,62,63,64,65,66,67,68,69,70], this comprehensive mini review restricts its scope to literature published within the last two years to provide a concise and updated overview. Here, we highlighted the key advancements in anticancer-proven quinolines and metal complexes incorporating quinoline-based ligands, detailing their mechanism of inhibition or activation across key proteins, enzymes, and signaling pathways. These targets span receptor tyrosine kinases (RTKs)—such as EGFR, VEGFR-2, PDGFR-α, c-Met, FGFR, and AXL—as well as cell-surface receptor complexes and integrin signaling modules, including TGF-βR, the TLR4-MD2/LPS heterooctameric complex, NTPDase, and FAK [71,72,73]. Inside the cell, quinolines modulate intracellular signaling and nuclear transcription factors (specifically STAT3, the MKK7-JNK pathway, BTK, SGK1, ERα, and MDM2-MDM4 E3 ligase modulators) alongside epigenetic factors like DNMT [74,75]. Furthermore, these compounds dictate cell cycle regulation and mitotic arrest by targeting CDKs, GAK, and KSP (Eg5), leading to the induction of cell cycle arrest in the G2/M phase. They also influence DNA integrity and translational modifications, acting on Topo-I and MetAP, while functioning as DNA intercalators and RAD51-BRCA2 interaction inhibitors [76]. Finally, quinolines address tumor microenvironment adaptation and efflux-mediated resistance via NOS, NQO1, and the P-gp efflux pump, as well as cellular metabolism and multidrug resistance (MDR) by modulating HDAC6/tubulin polymerization, ROS-mediated pathways, BCL-2, and caspase-3/8 activation (Figure 6) [77].

## 2. Anticancer Quinoline-Based Derivatives

### 2.1. Receptor Tyrosine Kinases (RTKs) as Initiators of Oncogenic Signaling

This group comprises cell-surface receptor tyrosine kinases that function as molecular ‘antennas’, sensing extracellular growth signals and transmitting them to intracellular signaling networks that regulate tumor cell proliferation, survival, angiogenesis, invasion, metastasis, and therapeutic resistance. Among these receptors, the epidermal growth factor receptor (EGFR) represents a classical model of mitogenic signaling, with activated mutations and gene amplification serving as important oncogenic drivers in a variety of solid tumors [78]. Vascular endothelial growth factor receptor 2 (VEGFR-2) is a major mediator of tumor angiogenesis, primarily through the regulation of endothelial cell proliferation, migration, and survival [79]. Platelet-derived growth factor receptor-a (PDGFR-α) contributes to tumor–stroma interactions, stromal cell recruitment, and vascular support, while aberrant PDGFR signaling is implicated in the pathogenesis of several malignancies, including gastrointestinal stromal tumors (GISTs) [80]. Subsequently, the hepatocyte growth factor receptor c-Met represents another significant oncogenic receptor tyrosine kinase that promotes tumor cell invasion and metastasis and can mediate bypass mechanisms underlying resistance to EGFR-targeted therapies [81]. Similarly, the fibroblast growth factor receptor (FGFR) family regulates multiple processes involved in tumor progression and has been implicated in the development of resistance to antiangiogenic therapies, including those targeting the VEGF/VEGFR axis [82,83]. Finally, AXL, a member of the TAM family of receptor tyrosine kinases, is increasingly recognized as a key regulator of epithelial-to-mesenchymal transition (EMT), metastasis, and resistance to multiple anticancer therapies [84].

In 2025, Şenol and coworkers designed novel 4-aminoquinoline derivatives functionalized with a phenylalanine methyl ester group [85] Synthesized compounds were assessed against human lung carcinoma A549 and human breast adenocarcinoma MCF-7, as well as healthy mouse embryonic fibroblasts NIH3T3. The most promising methyl (2-amino-7-chloro-3-cyanoquinolin-4-yl)-L-phenylalaninate (**1a**) and methyl (2-amino-6-chloro-3-cyanoquinolin-4-yl)-L-phenylalaninate (**1b**) (Figure 7)—at effective concentrations—displayed significant cytotoxicity against A549 and MCF-7 cells, exhibiting negligible toxicity toward non-cancerous cells. In A549 cells, compounds **1a** and **1b** achieved IC_50_ concentrations of 3.317 μM and 4.648 μM. In the case of the MCF-7 line, these derivatives inhibited cancer cell growth at IC_50_ = 7.711 μM and 6.114 μM, respectively. Furthermore, quinolines exhibited potent inhibitory activity against EGFR kinase, with compound **1b** showing an IC_50_ value of 0.058 µM, compared to 0.069 µM for **1a** and 0.002 µM for the reference drug erlotinib.

Based on these HOMO–LUMO analyses and global descriptors, significant trends were revealed among active compounds. It was found that these frontier orbitals were predominantly dispersed over the quinoline skeleton. Compounds **1a** and **1b** had lower HOMO–LUMO gaps and higher softness values than other synthesized quinoline derivatives, indicating their higher reactivity and possible ability to interact with targets.

Docking analyses of quinoline derivative **1a** into the EGFR active site indicated that heteroaromatic ring—similarly to erlotinib—forms a hydrogen bond with the carbonyl group of Met769. The primary amino group of the quinoline ring contributes to polar interactions. Both compounds **1a** and **1b** formed strong hydrogen bonds between the NH_2_ and Gln767 (Figure 7).

Abu-Hashem and coworkers synthesized intriguing fused heterocyclic compounds containing a pyridothiazolopyrimidine scaffold and a quinoline core to demonstrate their antiproliferative activity against multiple human cancer cell lines [86]. The most effective anticancer potential was observed for 2,3-dihydro-10*H*-isoxazolo[5′,4′:4,5]thiazolo[3,2-*a*]pyrido[2,3-*d*]pyrimidin-10-ones **2a** and **2b**, and pyrimido[4′,5′:4,5]thiazolo[2,3-*b*]pyrido[3″,2″-*e*]pyrimidine-11-one **3a**, and pyrimido[4′,5′:4,5]thiazolo[2,3-*b*]pyrido[3″,2″-e]pyrimidine-11-one **3b** derivatives (Figure 8), as well as their direct precursors.

These compounds were highly effective towards human carcinoma cell lines, including HeLa derivatives historically designated as oral carcinoma cells (KB, ICLAC-00010), nasopharyngeal carcinoma cells (CNE2, ICLAC-00474), and gastric carcinoma cells (MGC-803, ICLAC-00588), as well as authentic breast adenocarcinoma cells (MCF-7). It was found that all tested compounds had superior cytotoxicity, outperforming the reference drug, 5-fluorouracil. Specifically, **2a**, **2b**, **3a**, and **3b** derivatives demonstrated lower half-maximal inhibitory concentration IC_50_ values, ranging from 9.1 µM to 11.5 µM, compared to reference drug, which ranged from 11.5 µM to 13.1 µM. The highest susceptibility was observed against the drug-sensitive KB cells, where compound **3a** reached an IC_50_ value of 9.1 µM.

To rationalize these in vitro findings and elucidate the potential mechanism of action, molecular docking simulations were performed against key oncogenic targets, including epidermal growth factor receptor EGFR. The computational studies strongly supported the biological data, revealing favorable docking scores. For instance, 4-amino-3-benzoyl-2-oxo-8-phenyl-10-(quinolin-2-yl)-pyrimido[4′,5′:4,5]thiazolo[2,3-*b*]pyrido[3″,2″-e]pyrimidine-11-one (**3b**) displayed exceptional affinity for EGFR (PDB ID:1M17) with an estimated binding free energy (Δ*G*_calc_) of —12.00 kcal/mol, driven by key conventional hydrogen bonds with Asp831 and Cys773. The binding energy of **3b** exceeds that of 5-fluorouracil, which showed a calculated binding free energy (Δ*G*_calc_) of −8.2 kcal/mol. Additionally, compound engages in non-hydrophilic interactions with various amino acids, including π-alkyl Leu820, Leu764, Ala719, Val702, and π-anion interactions with Asp776. The binding interactions also include π-sulfur interaction with Met742 (Figure 8).

In their recent study, Manni and coworkers utilized an integrated computational and experimental framework to design, synthesize, and structurally verify a novel series of toluidine-based compounds as potential targeted anticancer agents [87]. Among the synthesized libraries, ethyl 6-methyl-4-(2-methyl-5-(3-morpholinopropyl)-4-oxothiazolidin-3-yl)-2-(thiophen-2-yl)quinoline-3-carboxylate (**4**) (Figure 9) stood out during antiproliferative screening.

This quinoline-based compound **4** was identified as a lead candidate displaying potent cytotoxicity against hepatoblastoma cells HepG-2 (IC_50_ = 7.84 μM), alongside a highly favorable safety margin for normal human fibroblast cells WI-38 (IC_50_ = 67.75 μM). To uncover the mechanism of action, further studies focused on evaluating VEGFR-2 inhibition, cell cycle progression, and apoptosis induction. These experimental findings were complemented by in silico studies, comprising molecular docking, molecular dynamics simulations, ADMET profiling, and density functional theory (DFT) calculations to characterize binding stability, molecular interactions, and drug-like properties. An enzymatic assay revealed that quinoline derivative **4** possessed VEGFR-2 inhibitory effects, yielding a sub-micromolar IC_50_ value of 0.55 µM—reference drug sorafenib achieved a value IC_50_ = 0.17 µM. Flow cytometry analyses demonstrated that treatment with compound **4** disrupts the cell cycle kinetics in HepG-2 cells, primarily by inducing a blockage at the G0/G1 checkpoint. Specifically, the G0/G1 cell population increased from 49.61% to 72.86%. The cell arrest was accompanied by a significant decline from 35.44% to 22.32% in the S phase fraction. This indicates a strong suppression of active DNA replication. Further to validate the apoptotic pathway triggered by compound **4,** an ELISA assay was used. Treatment of HepG-2 cells with **4** led to a striking elevation of active caspase-3 reaching 530.21 pg/mL level compared to a baseline of 99.69 pg/mL in the untreated control group.

In silico docking simulations into the VEGFR-2 reveal that compound **4** displays a high-affinity binding pose characterized by a docking score of Δ*G* = −8.50 kcal/mol. The hydrophobic core of compound **4** which consists of quinoline and thiophene rings fits into the pocket via key π-alkyl contacts with Pro1068, Leu1049, Ile888, Ala881, and Leu889 (Figure 9). Beyond the crucial hydrogen bond with Glu885 (2.23 Å), the side chain terminated with morpholine acts as a flexible modification vector targeting Arg1027, Arg1066, and Pro1068 near solvent-exposed regions. Within the ATP-binding domain, compound **4** participates in π-cation interactions with Asp1046 and additional structural anchoring provided by a carbon-hydrogen bond with Arg1066. Ultimately, the alignment between computational predictions and experimental data establishes quinoline-based compound **4** as VEGFR-2-targeted anticancer lead suitable for further optimization.

In 2025, Kiliccioglu and coworkers evaluated a novel series of quinoline–chalcone hybrids, focusing on their anticancer, antimicrobial, and in silico profiles [88]. Among the compound’s library, (*E*)-1-(4-(1*H*-imidazol-1-yl)phenyl)-3-(2-methoxy-6-methyl-quinolin-3-yl)prop-2-en-1-one (**5**) (Figure 10) displayed exceptional anticancer activity against gastrointestinal cancer panels (AGS, HepG2, and HCT116).

Quinoline–chalcone hybrid **5** emerged as the primary antiproliferative lead, demonstrating superior efficacy to oxaliplatin, particularly in AGS gastric cancer cells. Mechanistically, compound targets the intrinsic mitochondrial pathway to drive apoptosis and suppress the epithelial–mesenchymal transition (EMT) in AGS lines, acting synergistically to enhance the reference drug potency. Notably, **5** proved selectively cytotoxic to tumor cells, sparing healthy HUVEC control cells. Quinoline derivative **5** demonstrated significant experimental IC_50_ values across AGS, HepG2, and HCT116 cell lines (7.20 µM, 16.65 µM, and 6.47 µM at 48 h, respectively). Quantum chemical insights (DFT, MEP, RDG, ELF, LOL, and ALIE) successfully displayed its electronic properties. Cytotoxic inhibitory activities of the compounds correlated well with the binding scores obtained from the molecular docking simulations, which identified VEGFR-2 and EGFR as preferential targets with nanomolar binding affinities. Finally, ADME profiling confirmed favorable drug-likeness properties for this scaffold.

Following the initial synthetic step described by Sabbar in 2022 [89], further biological investigations established the 3-benzamido-3-phenyl-2-(4-((quinolin-8-yloxy)methyl)-1*H*-1,2,3-triazol-1-yl)propanoic acid (**6**) (Figure 11) as a promising anticancer drug candidate.

In their study, Hussein and collaborators demonstrated that this 1,2,3-triazole and quinoline-based compound exerts potent antiproliferative and anti-angiogenic effects across ex vivo rat aorta and in vivo chorioallantoic membrane models [90]. Mechanistically, compound suppresses VEGF-induced tube formation in human umbilical vein endothelial cells HUVECs and inhibits the VEGFR-2 gene in colorectal carcinoma HCT116 and ER-positive breast adenocarcinoma MCF7 cell lines.

In the RAR assay, compound **6** demonstrated anti-angiogenic activity (IC_50_ = 18.91 μg/mL; 38.32 μM) and anti-proliferative effects on HUVEC, colon cancer, and breast cancer cells, achieving IC_50_ values of 68.85 μg/mL (139.51 μM), 124.75 μg/mL (252.78 μM), and 112.31 μg/mL (227.57 μM), respectively. Moreover, it was demonstrated that at a concentration of 200 μg/mL (405.26 μM) derivative **6** decreases the expression of the VEGFR-2 gene in Kaposi’s Sarcoma (KS) cells.

Molecular docking and molecular dynamics simulations confirmed that quinoline-based compound **6** induces higher conformational rigidity and stability in the VEGFR-2 structure than axitinib. Coupled with favorable ADME characteristics, these recent results justify future structural alterations and preclinical trials to optimize its therapeutic efficacy.

Yadav and coworkers described the strategic design and synthesis of a novel library of fluorinated quinoline analogs [91]. This study was coupled with an extensive evaluation of their anticancer profiles in both monolayer cultures and 3D tumor spheroids. Among the synthesized libraries, derivatives **7a**, **7b**, and **7c** (Figure 12) emerged as highly potent lead compounds and exhibited profound cytotoxic selectivity toward triple-negative breast cancer (TNBC, MDA-MB-468) cells (IC_50_ = 0.02–0.03 µM) and double-positive MCF7 cell lines (IC_50_ = 0.02–0.03 µM) over the reference drug cisplatin (IC_50_ = 1.6 µM and 0.9 µM). Crucially, these candidate structures maintained an excellent safety profile, not damaging non-tumorigenic breast epithelial cells (MCF-10A, IC_50_ ≥ 100 µM).

According to structural analysis, the observed cytotoxicity is caused by ester functionality combined with fluorine on the quinoline core. Detailed structure–activity relationship (SAR) mapping revealed a clear topographical preference: the *meta* position is ideal for anchoring electron-withdrawing groups, whereas bulky substituents are better tolerated at the *para* position for optimized potency. Mechanistic assays demonstrated that compounds **7a**, **7b**, and **7c** strongly attenuate colony formation and trigger intracellular reactive oxygen species (ROS) accumulation in TNBC targets. Notably, **7b** and **7c** successfully compromised the structural integrity and volume of 3D TNBC spheroids (MDA-MB-468 and 4T1), validating their predictive in vivo efficacy.

These empirical trends were robustly mirrored by molecular docking simulations, which identified the crucial TNBC biomarkers CK2 and VEGFR2 as high-affinity targets for **7a** and **7c**, respectively. Compound **7a** displayed an exceptionally strong docking score of −9.887 kcal/mol with TNBC-relevant protein Casein kinase 2 (CK2, PDB: 7L1X), achieving the best binding score. This suggests excellent structural fitting and interaction with the protein. Key residues such as Val66, Ile174, Met163, and His115 were found to be crucial for forming hydrophobic and ionic interactions (Figure 12). On the other hand, the quinoline-based ethyl 6-(4-(benzyloxy)-3-fluorophenyl)-4-hydroxy-2-methylquinoline-3-carboxylate (**7c**) showed a highly favorable docking score of −9.366 kcal/mol, indicating an excellent binding potential to vascular endothelial growth factor receptor 2 (VEGFR2, PDB: 4ASE). An extensive network of residues was involved in binding interactions, including Phe918, Cys1045, Asp1046, and Ala866. According to this result, compound **7c** is likely to possess a strong and specific fit within the active site of VEGFR2. There is a possibility that fluorinated quinoline-based compounds **7a**, **7b**, and **7c** could be used to combat drug-resistant breast cancers. However, these compounds have a metabolically labile ester functionality that could limit their therapeutic application. Therefore, it is essential that future refinements focus on modifications of the ester group to enhance resistance to enzyme hydrolysis.

Tumor growth and metastasis are both linked to abnormal or uncontrolled angiogenesis. An angiogenic switch is activated by a large number of pro-angiogenic factors released by tumors during early stages of tumor formation. As a result, peripheral blood vessels form at the tumor site, bringing blood and oxygen to the site [92].

Several angiogenesis factors and receptors have been developed as targets for anticancer drugs in recent years, including anti-angiogenic inhibitors. Crucial to angiogenesis is platelet-derived growth factor (PDGF), specifically the interaction between its receptors (PDGFRs)—which possess protein tyrosine kinase activity—and their ligands [93]. Generally, the human body maintains a balance between proangiogenic and antiangiogenic factors and regulates the production of blood vessels. It is apparent, however, that aberrant overexpression of PDGFRs/PDGF contributes to the progression and invasion in the case of malignant tumors. In particular, PDGFR-α—a transmembrane glycoprotein dimer, a type III receptor tyrosine kinase—converts extracellular signals into intracellulars ones. This activates cells’ growth and proliferation. In the PDGFR-mediated intracellular pathway, upon binding of dimeric ligands, they stimulate receptor dimerization and phosphorylation of tyrosine residues. The activated PDGFR-α complex triggers JAK/STAT, PI3K/AKT, and Ras-MAPK/ERK signaling. As a result, PDGFRs have emerged as a target for treating tumor-related disorders, and several PDGFR blockers have been developed.

This line of research is currently being continued by Wang and coworkers who designed and synthesized novel platelet-derived growth factor receptor-α (PDGFR-α) inhibitors based on 4-anilinoquinoline scaffold. Novel compounds were designed as hybrids based on approved tyrosine kinase inhibitors—lenvatinib, ripretinib, and sorafenib—and evaluated in vitro for both their anticancer activity against human colon cancer HT-29 cells and inhibitory effects on PDGFR-α kinase [94]. The most prominent quinoline-based hybrid **8** (Figure 13) exhibited stronger antiproliferative activity against HT-29 cells than sorafenib IC_50_ = 0.67 µM vs. IC_50_ = 3.55 µM. In particular, this compound at a concentration of at 10 μM achieved the highest kinase inhibitory rate towards PDGFR-α (98.85%) in 76 different kinase selectivity profile assays.

Furthermore, to elucidate the potential binding of designed quinoline-based compound **8** to the ATP site of PDGFR-α, a molecular docking was performed using the reported lead compounds lenvatinib, ripretinib, and sorafenib and the crystal structure of protein. Specifically, the diphenylurea moiety contributes to bonding with PDGFR-α5p through conventional hydrogen bonding with Thr674 and aromatic hydrogen interactions with Glu644 and Glu675.

In 2025, Bhukya and coworkers prepared a novel series of quinoline-based 1,2,3-triazoles utilizing Suzuki–Miyaura cross-coupling and metal-free multicomponent reactions [95]. Specifically, compound bearing a chlorine atom at the R^2^ position of the phenyl ring **9a** (Figure 14) showed strong cytotoxicity against breast adenocarcinoma MCF-7, lung adenocarcinoma A-549, and hepatoblastoma HepG2 cell lines, with IC_50_ values of 9.25 μM, 9.56 μM, and 10.56 μM, respectively. Compound **9d** (Figure 14), which contains chlorine substituent and methoxy group, also demonstrated high potency, yielding IC_50_ values of 10.49 μM, 10.27 μM, and 11.27 μM against these three cancer cell lines. Another highly active derivative, compound **9c** (Figure 14), characterized by chlorine and iodine substitution pattern, achieved IC_50_ values of 11.40 μM (MCF-7), 10.42 μM (A-549), and 12.32 μM (HepG2). Additionally, compound **9b** (Figure 14) displayed marked activity, with IC_50_ values of 10.42 μM, 12.97 μM, and 13.05 μM for the respective cell lines. Cytotoxicity assays against non-cancerous Hek-293 cells confirmed the safety profile of these derivatives.

In silico molecular docking and screening indicated that these structures possess favorable drug-likeness parameters and establish key binding interactions within the active site of fibroblast growth factor receptor 1 (FGFR1). Quinoline-triazole derivative **9a** exhibited the highest binding affinity with a score of −10.5 kcal/mol. The nitrogen atoms of its 1,2,3-triazole moiety acted as a critical pharmacophoric anchor, forming two conventional hydrogen bonds with the hinge-region residue Ala564 (Figure 14). This interaction was strongly reinforced by an extensive network of hydrophobic and π-mediated contacts with Leu484, Ala488, Phe489, Val492, Ala512, Ile545, Val561, Ala564, Asn568, Glu571, and Leu630. Compound **9d** yielded a potent docking score of −10.2 kcal/mol, perfectly mimicking the energy profile of lenvatinib. Structurally, quinoline derivative containing R = OCH_3_ substituent maintained the characteristic dual hydrogen bonding with Ala564. Its methoxy group and chlorine atom also permitted for a usage of hydrophobic sub-pockets via specialized aromatic stacking π–π T-shaped with Phe489 and multiple aliphatic and π-alkyl contacts with Ile545 and Val561 (Figure 14).

The hepatocyte growth factor (HGF/c-Met) signaling pathway plays a crucial role in regulating cell proliferation, migration, survival, and tissue regeneration. Abnormal activation of c-Met has been involved in tumor growth, invasion, metastasis, and resistance to anticancer drugs [96]. The quinoline scaffold is a valuable structural motif in the design of kinase inhibitors owing to its ability to promote favorable interactions within kinase-binding pockets [97,98]. Accordingly, the combination of quinoline and triazolo–tetrazine scaffolds may represent a promising strategy for the development of novel c-Met inhibitors with potential anticancer activity.

Yang et al. developed two series of [1,2,4]triazolo[4,3-*b*][1,2,4:5]tetrazine derivatives containing quinoline or quinolon-4(1*H*)-one moiety and evaluated for their antitumor and c-Met inhibitory activities [99]. The selected compounds **10a**–**e** (Figure 15) were found to be highly effective against lung cancer A549 and H460 cell lines with IC_50_ values in the range of 2.97–16.50 μM. Among them, the most active derivative **10a** exhibited comparable cytotoxicity to the well-known ErbB family inhibitor afatinib, which was used in that study as a reference antitumor agent (IC_50_ = 2.97–3.16 µM vs. IC_50_ = 2.42–3.65 µM).

In the HTRF immunoassay, compounds **10a**–**e** demonstrated potent inhibitory activity against c-Met kinase, with IC_50_ values ranging from 0.42 µM to 2.24 µM. Notably, **10a** again emerged as the most active derivative, exhibiting the highest inhibitory potency (IC_50_ = 0.42 µM), consistent with its activity observed in an in vitro cytotoxic assay. Although all tested derivatives were less potent than the reference inhibitor staurosporine (IC_50_ = 0.042 µM), the results indicate that the antitumor activity of compounds **10a**–**e** may be, at least in part, related to their ability to inhibit c-Met kinase. The particularly pronounced activity of **10a** further supports its identification as a lead compound in this series. These experimental findings were completed by in silico studies, comprising molecular docking of **10a**, which revealed that the molecule was well embedded in the active pocket of receptor (Figure 15). Molecular docking studies showed that compound **10a** interacts with the c-Met binding pocket via three hydrogen bonds with Met1160 and Asp1222, as well as additional hydrophobic interactions such π–π bond with Tyr1230 and C-H-π interaction with Met1211.

In 2025, La Monica and coworkers successfully employed a molecular hybridization approach to design, synthesize, and evaluate a novel series of 4-(4-benzoylpiperazin-1-yl)-6-nitroquinoline-3-carbonitrile derivatives as potential anticancer agents [100]. Preliminary in silico ADMET and drug-likeness screening demonstrated favorable profiles for the designed hybrids, justifying the development of an efficient synthetic strategy to afford them. The in vitro biological screening revealed potent anticancer efficacy, particularly against the renal cell carcinoma (RCC) cell line UO-31. Notably, the derivatives **11a** and **11b** (Figure 16) were the most promising, showing a more balanced antiproliferative/cytotoxic profile in the one-dose assay (10^−5^ M), with growth percentages (G%) values of 6.68 and 19.40, respectively. Special attention should also be paid to compound **11b**, which features a bromine substituent on the side of phenyl fragment. Unlike the other analogs, **11b** showed a significantly mean G% of 50.85, demonstrating an overall remarkable antiproliferative profile with low to weak cytotoxic effects. Interestingly, **11b** also displayed robust antiproliferative activity against other aggressive and resistant malignancies, including non-small cell lung cancer (NSCLC), central nervous system (CNS), and breast cancers, achieving G% near zero.

Extensive induced-fit molecular docking and molecular dynamics simulations provided critical mechanistic insights, highlighting strong binding affinities for key oncogenic proteins driving RCC progression, namely AXL receptor tyrosine kinase (AXL), RAF proto-oncogene serine/threonine-protein kinase (Rapidly Accelerated Fibrosarcoma, C-RAF), and LSD1 (Lysine Demethylase 1A). Detailed ligand–protein contact analysis revealed that the quinoline core, the piperazine ring, and the benzoylamino moiety are crucial structural motifs responsible for stable complex formation. These findings underscore the potential of these hybrid scaffolds for future optimization of potential anticancer drugs.

### 2.2. Cell-Surface Receptor Complexes and Integrin Signaling Modules

This section examines the key cell-surface complexes and their cytoplasmic signaling partners that dictate malignancy. It highlights how these molecules act as critical checkpoints to regulate immune evasion, inflammation, and extracellular matrix interactions during cancer progression, focusing on four primary drivers: transforming growth factor-β receptor (TGF-βR), a molecular switch acting as a tumor suppressor in early stages and a promoter of metastasis and fibrosis in late-stage disease; TLR4-MD2/LPS heterooctameric complex, a pattern recognition receptor complex that drives chronic, bacterial LPS-mediated inflammation to promote tumor cell survival; nucleoside triphosphate diphosphohydrolase (NTPDase/CD39), an ecto-enzyme responsible for degrading extracellular ATP into AMP, initiating the immunosuppressive adenosine pathway; and focal adhesion kinase (FAK) which is a cytoplasmic kinase tightly linked to integrin signaling that governs cell adhesion, motility, and metastatic dissemination.

The transforming growth factor-beta (TGF-β) type 1 receptor (TGFβR1) is a member of the serine–threonine kinase receptor family involved in the TGF-β signaling pathway. This pathway is a key regulator of cell proliferation, differentiation, apoptosis, and extracellular matrix production. Under normal signaling conditions, TGF-β promotes cellular dormancy and apoptosis, acting as a tumor suppressor. In pathological conditions such as inflammation or cancer, it activates processes promoting tissue remodeling and cancer progression. Given the pivotal role of the transforming growth factor β (TGF-β) type 1 receptor in the tumor microenvironment, targeting TGFβR1 offers a viable approach for cancer treatment, making small-molecule TGFβR1 inhibitors a promising therapeutic strategy for malignant tumors [101].

In 2025, Liu et al. identified a novel series of 4-((3-(tetrahydro-2*H*-pyran-4-yl)-1*H*-pyrazol-4-yl)oxy)quinoline derivatives as potential TGFβR1 inhibitors [102]. Among them, the most potent derivative, **12** (Figure 17), effectively suppressed SMAD2/3 phosphorylation and inhibited mouse hepatocellular carcinoma cell H22 viability, yielding IC_50_ values of 12 nM and 65 nM, respectively.

Compound **12** demonstrated superior in vivo efficacy in an H22 xenograft mouse model compared to galunisertib, achieving a 79.6% tumor growth inhibition (TGI) rate (*vs*. 59.1% for galunisertib) and exhibiting excellent tolerability at 60 mg/kg BID. The treatment inhibited tumor proliferation and enhanced CD8+ T-cell infiltration, indicating a potent antitumor effect linked to reduced SMAD2 phosphorylation. Quinoline derivative **12** demonstrated favorable pharmacokinetic profiles and achieved robust in vivo antitumor efficacy. Crucially, **12** also exhibited a powerful synergistic pro-apoptotic effect when combined with sorafenib, establishing it as a lead structure for the development of innovative anticancer therapeutics. To explore the binding mechanism and rationalize the anti-TGFβR1 potency of compound **12**, computational modeling studies were conducted (Figure 17).

Compound **12** showed a spatial orientation highly congruent with LY-3200882—a highly selective inhibitor of the transforming growth factor-beta (TGF-β). Analysis of the trajectory confirmed a key conserved interaction: a hydrogen bond between the quinoline scaffold and the hinge residue His-283, which remained stable for 90% of the simulation time. Deep pocket penetration was achieved by the cyclopropyl group, anchoring a water-mediated hydrogen bond network with Glu-245, Tyr-249, and Asp-351. Collectively, these findings clarify the molecular basis of its potency and assist in future inhibitor optimization.

Activation of TLR4 by bacterial lipopolysaccharides triggers breast cancer development via NF-κB, MAPK, and PI3K/Akt signal transduction pathways, resulting in inflammatory cytokine production, epithelial–mesenchymal transition, migration, and metastasis. Batin Rahaman et al. synthesized 4-aminoquinoline-thiazolidinone hybrids as anticancer drugs targeting the TLR4-LPS axis [103]. Among the derivatives exhibiting low micromolar activity against breast cancer MCF-7 cell line and triple-negative breast cancer MDA-MB-231 cell line, compound **13** (Figure 18) was identified as the lead candidate. It demonstrated significant potency in MCF-7 (IC_50_ = 2.36 µM) and MDA-MB-231 (IC_50_ = 3.26 µM) cell lines, while its CC_50_ value of 23.43 µM in non-tumorigenic NKE cells indicated preferential activity toward malignant cells. Moreover, **13** exhibited the most promising anti-inflammatory activity in macrophage cell line RAV267.7 (IC_50_ = 2.38 µM). Further, mechanistic analysis in MDA-MB-231 cells demonstrated that 4-aminoquinoline–thiazolidinone hybrid **13** induces apoptosis in cancer cells accompanied by G2/M cell cycle arrest and increased intracellular ROS accumulation. Moreover, at a concentration of 1.25 µM, **13** reduced LPS-mediated cancer cell migration, an effect that may be associated with inhibition of TLR4-LPS axis.

Molecular dynamic simulations performed for compound **13** revealed that the binding stability of the compound within the Toll-like receptor 4 (TLR4) pocket is sustained by a network of non-covalent interactions. Hydrogen bonds formed with key residues, including Ser441 and Tyr131, contribute significantly to anchoring the ligand. This binding is further reinforced by hydrophobic contacts with Phe121 and Phe126, which are crucial for locking the molecule inside the hydrophobic subpockets. Additionally, water bridges involving Glu439 underscore the active role of solvent molecules in mediating complex stability. Ultimately, this interaction profile—hydrogen bonding, hydrophobic forces, and water-mediated bridges—provides a comprehensive understanding essential for drug design and optimization.

Nucleoside triphosphate diphospho-hydrolases (NTPDases)—ectonucleotidases—are a family of enzymes responsible for hydrolyzing nucleotides like ATP, UTP, ADP, and UDP to monophosphates [104]. Ectonucleotidases have the potential to emerge as anticancer therapeutic targets, given their critical role in regulating purinergic signaling within the extracellular tumor microenvironment. Human NTPDases and ENTPP1 hydrolyze ATP and other nucleotides, producing AMP, which is then transformed into adenosine by ecto-5′-nucleotidase/CD73. The resulting accumulation of extracellular adenosine stimulates A_2_A and A_2_B receptors and suppresses the affinity of T cells, B cells, and NK cells, thereby enabling tumor immune escape. Thus, inhibiting ectonucleotidase may help conserve immune-stimulating ATP, reduce adenosine synthesis, and restore antitumor immunity [105].

Ishaq et al. [105] synthesized quinoline-6-carboxamide derivatives and investigated their ectonucleotidase-inhibitory and antiproliferative activities. The compounds were examined against h-NTPDase1, h-NTPDase2, h-NTPDase3, h-NTPDase8, h-ENPP1, and h-e5′NT/CD73, followed by cytotoxicity and antiproliferation studies in MCF-7 breast cancer cells [105]. Among the tested compounds, quinoline-6-carboxamide derivative **14** (Figure 19) was found as the most potent dual inhibitor of h-NTPDase 1 and h-NTPDase 2 (IC_50_ = 0.28 µM and 0.92 µM, respectively), which was supported by its docking score (–7.8 kcal/mol and –8.5 kcal/mol, respectively).

Moreover, the cytotoxicity profile of compound **14** in MCF-7 breast cancer cells demonstrated moderate anti-proliferative effect (48% cell viability at 100 μM). Overall, the anti-proliferative effect observed was moderate, the results of enzymatic inhibition assays and molecular docking studies collectively highlight quinoline-6-carboxylic acid derivative **14** as promising candidate for the development of ectonucleotidase inhibitors with potential applications in cancer immunotherapy.

Focal adhesion kinase (FAK) belongs to the cytoplasmic non-receptor tyrosine kinase family. An enzyme is essential for cellular homeostasis due to transducing signals between the extracellular matrix (ECM) and the intracellular space. Therefore, FAK acts as a central mediator of cell adhesion, motility, survival, and proliferative pathways. In malignant cells, aberrant upregulation and hyperactivation of FAK drive tumor progression, positioning this kinase as a critical therapeutic vulnerability to disrupt metastatic dissemination and chemoresistance [106].

It was found that targeting both the epidermal growth factor receptor (EGFR) and FAK simultaneously is a potent strategy because FAK activation is a well-known resistance mechanism to EGFR inhibitors, e.g., erlotinib. When the EGFR pathway is blocked, cancer cells may upregulate FAK to bypass this signaling in and proliferate. Inhibiting both pathways at the same time could be beneficial in preventing this bypass mechanism. That would make treatment far more effective at inhibiting tumor growth.

In 2026, Elbarbry and coworkers assessed the inhibitory effects against membrane-bound cytochrome P450 enzymes: CYP1A2, CYP2A6, CYP2C9, CYP2C19, CYP2D6, CYP2E1, and CYP3A4 of 4-propoxy-2-arylquinolines **15a**, **15b**, and **15c** (Figure 20) [107]. These compounds were designed in 2022 as dual epidermal growth factor receptor (EGFR) and focal adhesion kinase (FAK) kinase inhibitors [108].

Quinoline-based dual EGFR/FAK inhibitors **15a**, **15b**, and **15c** were tested using pooled human liver microsomes (pHLM) and validated LC-MS/MS or HPLC-UV assays to assess their potential to inhibit major human CYP enzymes. Evaluating these inhibitory effects during preclinical development is vital for predicting metabolic liabilities, safety risks, and drug–drug interactions. The study found that CYP2A6, CYP2D6, and CYP2E1 were the most affected enzymes. Quinolines exhibited different and selective inhibition profiles toward major CYP enzymes, reaching the following half-maximal inhibitory concentrations IC_50_ values in the range from 0.04 µM to 0.189 µM.

### 2.3. Intracellular Signaling Cascades and Nuclear Transcription Factors

The biochemical pathways that transmit signals from surface receptors down through the cytoplasm into the nucleus include signal transducer and activator of transcription 3 (STAT3), mitogen-activated protein kinase 7 (MKK7-JNK pathway), a key MAPK cascade regulating cellular stress responses, and proliferation, and Bruton’s tyrosine kinase (BTK), a crucial node in B-cell receptor (BCR) signaling. Moreover, glucocorticoid-regulated kinase (SGK1), which is involved in cell survival, metabolic reprogramming, and resistance to chemotherapy, and α-estrogen receptor (ERα)—a nuclear hormone receptor acting as a direct transcription factor that drives hormone-dependent cancers. Furthermore, quinoline-based compounds may promote the proteasomal degradation of the oncogenic MDM2-MDM4 complex.

Molecular hybridization of quinoline pharmacophores is a plausible approach for developing multifunctional pharmaceutical medicines, as these heterocyclic scaffolds are associated with anticancer activities. Pyrazoline and quinoline are favored heterocyclic scaffolds with recognized anticancer and kinase-modulating properties. Accordingly, Singh et al. synthesized and evaluated a series of pyrazoline–quinoline conjugates using both complementary in vitro and in silico methodologies, with specific emphasis on cytotoxic efficacy against A549 human lung cancer cells [109].

The selected compounds **16a**, **16b**, **16c**, **16d**, **16e**, and **16f** (Figure 21) have the most significant antiproliferative activity against A549 human lung cancer cells, with IC_50_ values of 3.68 µM to 7.82 µM, all lower than doxorubicin (IC_50_ = 9.48 µM) under identical conditions. The dihydroxy-substituted derivative **16d** was the most active compound, followed by the halogenated derivative **16a** and the fluoro–hydroxy derivative **16c**. The structure–activity relationship indicates that hydroxyl groups promote hydrogen bonding interactions, whereas fluorine and chlorine substituents improve lipophilicity and target binding.

Future anticancer therapy research should prioritize compounds **16a**, **16b**, **16c**, **16d**, **16f**, and particularly **16d**, with a focus on tumor selectivity and determining whether these compounds cause apoptotic cell death, alter cell cycle progression, generate reactive oxygen species, and affect oncogenic signaling. Docking studies indicated substantial interactions with EGFR and STAT3, notably **16a** against EGFR and **16d** against STAT3 (Figure 21). However, direct pharmacologic suppression of these targets has not been experimentally verified.

The JNK (c-Jun *N*-terminal kinases) are serine–threonine kinases belonging to the mitogen-activated protein kinase (MAPK) family and play a key role in cellular responses to stress, inflammation, and apoptosis. The JNK signaling pathway controls both proapoptotic and antiapoptotic processes in various cell types. Dysregulation of the JNK pathway plays a crucial role in cancer growth, progression, and metastasis [110,111].

In 2025, a research team led by Ma described the synthesis and biological evaluation of novel 8-(benzyloxy)-5,7-dibromo-2-methylquinoline derivatives as targeted anticancer agents against triple-negative breast cancer cells (TNBC) [112]. Among them, compounds **17a** and **17b** (Figure 22) demonstrated high cancer cell growth inhibitory activity with IC_50_ < 1 μM. Particularly, 5,7-dibromo-8-((3-iodobenzyl)oxy)quinoline (**17b**) emerged as the leading candidate reaching an IC_50_ of 0.81 μM. Western blot analysis, flow cytometry, and Hoechst staining confirmed that compound **17b** significantly inhibited TNBC cell proliferation by inducing G2/M cell cycle arrest and activating the MKK7-JNK signaling pathway to trigger caspase-dependent apoptosis in MDA-MB-468 cells. Compound **17b** also displayed a favorable selectivity profile through significantly lower toxicity to normal human mammary epithelial MCF-10A cells (IC_50_ = 43.56 μM) and bronchial epithelial 16-HBE cells (IC_50_ = 60.45 μM). Anticancer effects were accompanied by limiting the migratory capacity of tumor cells. Importantly, quinoline derivative **17b** led to a significant increase in the level of phosphorylated JNK kinase (p-JNK) and activation of mitogen-activated protein kinase 7 (MKK7). Pharmacological inhibition of the JNK signaling pathway with the SP600125 inhibitor, as well as silencing JNK expression using siRNA, completely abolished its proapoptotic properties. These results confirm that activation of the MKK7-JNK signaling pathway plays a key role in inducing caspase-dependent apoptosis through both extrinsic and intrinsic pathways. Data obtained here support further structural optimization of 5,7-dibromo-2-methyl-8-hydroxyquinoline and indicate that activation of the JNK pathway may be a promising target for therapeutic development. In addition, the obtained data suggest that modifying the structure of 5,7-dibromo-2-methyl-8-hydroxyquinoline scaffolds indicates that it may be the starting point for the development of novel JNK pathway activators as targeted anticancer therapeutics.

In 2025, Panduranga and their research team obtained a series of diversely functionalized quinoline–sulfonamide molecular hybrids via a multi-step synthetic approach and evaluated for their anticancer potential [113]. Quinolines were screened against a broad panel of cancer cell lines (HCT116, A549, U2OS, CCRF-CEM, Jurkat, MOLT-4, RAMOS, and K562), as well as non-cancerous control lines (MRC-5 and BJ). While all synthesized derivatives showed weak activity against A549, HCT116, and U2OS cells (IC_50_ > 50 μM), they exhibited significant, selective potency against specific hematological malignancies. Particularly, in Interleukin-2-inducible T-cell kinase (ITK)-high-expressing leukemia lines, compounds **18a**, **18b**, and **18d** (Figure 23) were identified as the most active candidates, displaying IC_50_ values of 7.43 μM (Jurkat), 5.57 μM (MOLT-4), and 13.19 μM (CCRF-CEM), respectively. Furthermore, screening against Bruton’s tyrosine kinase (BTK)-high expressing lymphoma cells highlighted the high potency of molecules **18c** (Figure 23) against RAMOS (IC_50_ = 2.76 μM) and **18a** against K562 (IC_50_ = 5.47 μM). Crucially, tested quinoline derivatives remained non-toxic to human lung and skin fibroblasts (MRC-5 and BJ) with IC_50_ values exceeding 50 μM. This remarkable selectivity makes the quinoline–sulfonamide framework a promising scaffold for targeted anticancer drug development, especially for ITK- and BTK-overexpressing blood cancers.

An emerging target in oncology is serum and glucocorticoid-regulated kinase 1 (SGK1) belonging to the serine/threonine kinase family. It was found that SGK1 is dysregulated in a variety of malignancies and promotes cancer cell survival, invasiveness, metastasis, and chemotherapeutic resistance. SGK1 displays similar structure and substrate specificity to the phosphatidylinositol 3-kinase (PI3K)/AKT signaling pathway [114,115,116].

Recently, based on the ‘hinge region occupied’ design strategy of the trifluoromethyl quinoline-based ‘hit’ compound [117], Yang and collaborators identified a novel derivative **19** (Figure 24) that inhibits prostate cancer progression by targeting SGK1 and modulating the PI3K/AKT pathway [118]. It was demonstrated that the compound **19** decreased proliferation and migration in human prostate cancer (PC3) and androgen-sensitive human prostate adenocarcinoma (LNCaP) cell lines. The compound displayed in vitro selective activity with assessed IC_50_ values of 0.10 µM and 0.17 µM. In vivo xenograft experiments on naked mice showed that the compound effectively reduced tumor growth without significant toxicity. These experiments were supported by molecular docking studies. In silico simulations from a recent study revealed a favorable estimated binding energy of −10.4 kcal/mol between compound **19** and SGK1. Crucially, the authors complemented these computational findings with robust experimental evidence, demonstrating that compound **19** successfully inhibits the activation of the PI3K/AKT signaling pathway. The stability of the **19**/SGK1 complex resulted in two stable hydrogen bonds with isoleucine (Ile179) and glutamate (Glu183) residues—bond lengths of 2.2 Å and 3.3 Å, respectively. Moreover, MD simulations were conducted over a 100 ns timeframe to assess the binding stability of the SGK1/**19** complex. Significant inhibitory effects on prostate cancer accompanied by computational results provide quinoline derivative **19** as a novel candidate for targeted antitumor therapy.

The nuclear hormone receptor superfamily includes estrogen receptors (ERs), which function as ligand-activated transcription factors and exist in two main isoforms: alpha (ERα) and beta (ERβ). Dysregulation of the signaling pathways associated with these receptors can lead to unchecked cellular proliferation, genomic instability, and subsequent tumor development, as well as gynecological disorders such as endometriosis and polycystic ovary syndrome (PCOS). In this context, estrogen acts as a primary endogenous ligand for the receptor protein. Upon binding, estradiol activates ERα, which subsequently stimulates the upregulation of specific genes responsible for promoting tumor growth. Consequently, over-expression of ERα is frequently observed during breast cancer progression. Targeting these pathways to displace estradiol from the protein-binding pocket or to modulate receptor interaction dynamics represents a key therapeutic strategy, which may also aid in alleviating associated clinical complications like edema. Moreover, the proliferation of tumor cells is directly stimulated through these specific estrogen–protein interactions [119,120].

Recently, Kumar and coworkers [121] synthesized a novel library of phenyl-substituted quinoline-1,2,3-triazole glycoconjugates, utilizing a copper(I)-catalyzed azide-alkyne cycloaddition (CuAAC) click strategy. In vitro anticancer evaluations against the human MCF-7 luminal A-type breast cancer cell line revealed that while most of the synthesized series remained inactive, specific derivatives containing explicit configurations demonstrated marginal antiproliferative activity. Specifically, the glucosyl–methoxy derivative **20a**, the glucosyl-fluorophenyl derivative **20b**, and the galactosyl quinoline-based compound **20c** (Figure 25) exhibited cytotoxicity with IC_50_ values of 131.25 µM, 135.95 µM, and 108.59 µM, respectively.

To explore the potential inhibitory mechanisms and target–ligand compatibility within the ERα binding domain, structure-based in silico molecular docking simulations were performed. The docking scoring functions revealed that derivatives **20a**, **20b**, and **20c** possessed exceptionally strong binding affinities within the ERα active site, yielding docking energies of −9.1 kcal/mol, −9.5 kcal/mol, and −8.9 kcal/mol, respectively. For example, compound **20c** formed a highly coordinated system featuring two hydrogen bonds linking the acetoxy oxygen atoms (3-acetyl and 6-acetyl sugar groups) to Trp383 and Leu536, respectively. Further locking within the pocket was driven by π–anion parameters via Asp351. The quinoline scaffold participated in π–alkyl interactions with Ala350 and Leu525, while Lys529 provided strong π–alkyl stabilization to the triazole linker (Figure 25). Computational simulations predicted a potential interaction between the synthesized quinoline-1,2,3-triazole glycohybrids and estrogen receptor alpha (ER-α). However, the evaluated compounds exhibited low to negligible in vitro anticancer activity, and direct experimental validation of ER-α targeting was not established.

Recently, Wang and colleagues demonstrated that quinolinol derivatives can effectively destroy cancer cells through multi-targeted actions, including the degradation of MDM4 and FTH1 proteins [122]. Identified through high-throughput screening, quinolinol derivative **21a** and its analog, **21b** (Figure 26), exhibit potent antitumor efficacy. It has been shown that the quinoline derivative **21b** covalently binds to over 1500 intracellular proteins and disrupts cellular networks of carbon, RNA, and amino acid metabolisms.

These quinolinols simultaneously promote the proteasomal degradation of the oncogenic MDM2-MDM4 complex, thereby unlocking p53-independent apoptosis, and induce the lysosomal degradation of ferritin heavy chain 1 (FTH1). MDM4 acts as a natural inhibitor of p53—responsible for detecting DNA damage and forcing defective cells to undergo apoptosis. The cancer cells frequently overproduce MDM4. As a result, they bind and block p53 which allows cancer cells to multiply uncontrollably. On the other hand, tumors are highly dependent on iron and require it for rapid growth and division. Ferritin heavy chain 1 is a core component of ferritin, which is the primary protein cage used to safely store iron inside our cells. By triggering the lysosomal degradation of FTH1, the free iron ions present in cancer cells can cause extensive oxidative stress. This ultimately leads to a specific type of iron-dependent cell death called ferroptosis. Compound **21a** was found to modulate the activity of the MDM2-MDM4 E3 ligase. A unique property of **21a** is its ability to induce cell death across different modes, such as triggering p53-independent apoptosis in leukemic cells and ferroptosis in pancreatic cancer cells. To further enhance potency of **21a**, subsequent structure–activity relationship studies led to the development of the derivative **21b**. This optimized analog demonstrated superior efficacy compared to conventional MDM2 inhibitors in melanoma models. Crucially, **21b** effectively eliminates melanoma cells that have acquired resistance to standard therapies. It was demonstrated that compound **21b** disrupts intracellular iron homeostasis, leaks toxic free iron into the cytoplasm, and triggers ferroptosis. Both compounds **21a** and **21b** showed high cytotoxic activity against A375 melanoma cells (IC_50_ = 0.51 µM and IC_50_ = 0.11 µM, respectively), whereas their 8-*O*-methylated analogs were inactive. This significant difference in IC_50_ values confirms that this efficacy is directly due to specific covalent chemical interactions and not to nonspecific toxicity. Authors claimed that synthesized quinolinol derivatives **21a** and **21b** could induce cancer cell death compared to specific MDM2 inhibitors and BRAF inhibitors. Consequently, the acceptable tolerability profile of these quinolinols establish them as novel class of multi-targeting anticancer agents.

### 2.4. DNA Methylation and Chromatin Architecture

DNA methylation is a crucial mechanism regulating gene expression, ensuring genome stability, participating in organismal development, maintaining cellular identity, and cell differentiation. This process is mediated by enzymes from the DNA methyltransferase (DNMT) family, which are key regulators of epigenetic gene control that catalyze the transfer of a methyl group from *S*-adenosyl-L-methionine (SAM) to the carbon atom at the 5-position of cytosine, most commonly in CpG dinucleotides. The DNMT family includes the enzymes DNMT1, DNMT2, DNMT3A, DNMT3B, and DNMT3L, which differ in their biological functions and structural structure. DNMT1 is responsible for maintaining methylation in the genome, while DNMT3A and DNMT3B participate in de novo methylation. DNMT2 and DNMT3L are noncanonical members of the DNA methyltransferase family. DNMT2 is mainly responsible for RNA, methylation, while DNMT3L, despite its lack of enzymatic activity, regulates the methylation process by stimulating the activity of DNMT3A and DNMT3B. DNA methylation disorders constitute one of the most important epigenetic changes observed in cancer [123,124,125].

In 2025, Zwergel and colleagues demonstrated that the introduction of basic side chains into the structure of quinoline derivatives successfully enhanced water solubility and inhibitory activity on DNMT enzymes [126]. The antiproliferative activity of the tested quinolines with *N*-alkyl groups was assessed using the A549 and H460 non-small cell lung cancer, HCT-116 colon cancer, HeLa cervical cancer, MDA-MB-231 breast cancer, and U937 histiocytic lymphoma cell lines.

Among the synthesized derivatives, quinolines **22a** and **22b** (Figure 27) exhibited the most promising profiles emerging as potent and selective inhibitors. Specifically, compound **22a** demonstrated nanomolar potency and selectivity against DNMT3A/3L (IC_50_ = 0.019 μM), whereas compound **22b**, with two basic chain functions, bearing a -CH_2_-N(CH_3_)_2_ group, selectively targeted DNMT1 (IC_50_ = 0.044 μM, selectivity index > 227). In cellular assays using HCT-116 colon cancer cells, compound **22b** exhibited the strongest antiproliferative activity with an IC_50_ value of 0.4 μM. Quinoline derivative **22b** triggers DNA damage, leading to cell cycle arrest in the S and G2/M phases along with subsequent apoptosis. Crucially, tests on isogenic HCT-116 p53/^−^ cells showed reduced antiproliferative effects, lower apoptotic rates, and decreased levels of cleaved caspase 3, p53, and *γ*H2AX, thereby confirming a p53-dependent mechanism of action linked to DNA damage.

### 2.5. Cell Cycle Regulation and Mitotic Arrest

Dysregulation of cell cycle control and mitotic progression is a fundamental feature of cancer, contributing to sustained and uncontrolled cellular proliferation. Consequently, key regulators of cell cycle checkpoints and mitotic machinery have emerged as important therapeutic targets. Cyclin-dependent kinases (CDKs) are serine–threonine kinases whose activity depends on the presence of a regulatory subunit, a cyclin [127]. They are recognized as key regulators of cellular processes such as cell proliferation, gene transcription, translation, and RNA metabolism. Therefore, inhibition of CDK activity is being investigated as a therapeutic strategy in cancer treatment. However, due to the involvement of CDKs in various pathological processes, they also represent potential targets in neurodegenerative, metabolic, cardiovascular, and autoimmune diseases [128].

In 2025, a research team led by Jia-Xing Lu utilized the biologically active quinoline scaffold to synthesize a novel series of quinoline-based dihydrazone derivatives, performing comprehensive structural, spectral, and stability characterizations via NMR, ESI-HRMS, IR, elemental analysis, UV–Vis, and fluorescence spectroscopy [129]. Systemic toxicological assessments indicated a favorable, low-toxicity profile with no detectable nitrosamine impurities. Screening via MTT assays revealed that all derivatives possessed significant antiproliferative activity against human cancer cell lines (stomach cancer BGC-823, liver cancer BEL-7402, breast cancer MCF-7, and lung adenocarcinoma A549), with IC_50_ values ranging from 7.01 µM to 34.32 µM. Crucially, no prominent toxicity was observed against normal human liver cells (HL-7702). Specifically, derivatives **23a** and **23b** (Figure 28) exhibited exceptional potency against MCF-7 cells IC_50_ = 7.02 µM and 7.05 µM, respectively). A dual-fluorescence assay using acridine orange/ethidium bromide (AO/EB) staining, flow cytometry, and ROS detection confirmed that **23a** and **23b** triggered a dose-dependent cytotoxic effect by inducing apoptosis in MCF-7 cells. Furthermore, molecular docking simulations indicated that these two compounds could bind to DNA through partial intercalation and identified CDK2 as a primary therapeutic target. Consequently, **23a** and **23b** represent chemotherapeutic leads for oncology drug development.

Furthermore, molecular docking simulations for compounds **23a** and **23b** within an active site of CDK2 revealed that molecular forces driving the binding include hydrogen bonding, π–π stacking, and electrostatic interactions. Specifically, **23a** establishes a key hydrogen bond via the nitrogen atom of its hydrazone scaffold in the Lys88 residue. Conversely, hydrogen bonding for **23b** is mediated by the hydrogen atoms of the hydrazone linkage interacting with the Arg199 residue. Structural analyses also revealed distinct π–π interactions; for the compound **23a**, the quinoline and pyrimidine rings stack with Ala201 and Asp92. In contrast, derivative **23b** participates in π–π stacking exclusively through its pyrimidine ring with the Asp92 residue, likely owing to the steric hindrance introduced by the benzene ring. Taken together, these docking outputs corroborate that CDK2 represents a viable biological target for both **23a** and **23b**.

Cyclin G-associated kinase (GAK) is a member of the numb-associated kinase (NAK) serine–threonine kinase family. It participates in numerous biological processes, such as cell cycle progression, centrosome maturation, clathrin-mediated endocytosis, epidermal growth factor receptor signaling, and lysosomal enzyme sorting [130]. GAK expression level may serve as a prognostic biomarker in prostate cancer, with its expression increasing with tumor progression to the androgen-independent form [131]. GAK has also been shown to be overexpressed in osteosarcoma cell lines and tissues, where it contributes to cell proliferation and survival [132].

In 2026, based on a cell phenotypic screening strategy, Gentz and coworkers identified 4,6-disubstituted quinolines as potent modulators of Ewing’s sarcoma (ES) cell viability [133]. Among the derivatives exhibiting low micromolar activity, structural optimization of hit compounds yielded thiosemicarbazones with enhanced potency. Structure–activity relationship (SAR) analysis demonstrated that the cytotoxicity of this thiosemicarbazone series is critically dependent on the nature of the substitution at the C-6 position of the quinoline core. The most prominent candidate with IC_50_ = 1.1 μM against the RD-ES cell line is represented by 2-((6-((3,5-dimethylphenyl)amino)quinolin-4-yl)methylene)hydrazinecarbothioamide (**24**) (Figure 29).

Docking analysis of this derivative **24** showed that its 3,5-dimethylphenyl ring fits well into the hydrophobic region of GAK (PDB: 5Y7Z), interacting with Val54, Lys69, and Ala67. The quinoline ring anchors in the hinge region, mediated by a crucial hydrogen bond between the quinoline nitrogen and the backbone amide of Cys126. Uniquely, hydrazinecarbothioamide moiety also forms two hydrogen bonds with the Leu46 backbone. It was suggested that the smaller, more flexible thiosemicarbazone group boosts the potency of compound compared to the bulkier hydrazone group.

Beyond their computational profiling, Gentz et al. [133] validated the mechanism of a new series of 4,6-disubstituted quinolines through extensive in vitro kinase inhibition assays against a broad panel of enzymes. The evaluation revealed a narrow selectivity profile, with the lead derivatives predominantly targeting NAK family kinases. Remarkably, the compounds demonstrated potent nanomolar inhibitory activity against cyclin G-associated kinase (GAK) in enzyme kinetic assays, confirming their targeted mechanism alongside their antiproliferative effects on Ewing Sarcoma cells.

Kinesin spindle protein, KSP (Eg5) is a microtubule-associated motor protein from the kinesin-5 family that plays a key role in the architecture and progression of mitosis. Structurally, Eg5 is a homotetramer with a bipolar configuration where pairs of catalytic motor domains are located at opposite ends of a central core. This three-dimensional structure allows the protein to simultaneously cross-link and slide into anti-parallal interpolar microtubules. Using the energy from ATP hydrolysis, Eg5 generates a mechanical force directed toward the plus (+) ends of interacting microtubules. This process generates a repulsive force that drives centrosome separation and, consequently, induces the formation, stabilization, and maintenance of the bipolar mitotic spindle [134,135]. Proper formation of bipolar spindles is essential for precise chromosome segregation. Blocking the Eg5 protein’s activity disrupts the mitotic apparatus and prevents centrosome separation [136]. This leads to the collapse of the spindle architecture into a characteristic unipolar phenotype. Furthermore, the structural defect activates the spindles assembly checkpoint (SAC), causing prolonged cell cycle arrest at the transition from pro-metaphase to metaphase. Ultimately, this prolonged checkpoint activation drives the cells toward a mitotic catastrophe and triggers the intrinsic apoptotic pathway [137]. Since cancer cells exhibit a significantly higher mitotic index and are more dependent on the division apparatus than quiescent normal cells, targeting Eg5 avoids peripheral neurotoxicity—a major limitation of classical microtubule-destroying chemotherapeutics (e.g., taxanes) [138]. Moreover, Eg5 represents a strategic target for overcoming chemoresistance. This is particularly relevant in highly aggressive malignancies, such as triple-negative breast cancer (TNBC), which characteristically exhibit overexpressed mitotic machinery and rapid recurrence rates.

In 2025, Alegaon and collaborators designed and synthesized quinoline-based Schiff bases targeting Eg5 [139]. The synthesized compounds were evaluated in vitro, including enzyme assays and cytotoxicity tests against the triple-negative breast cancer cell line MDA-MB-231 and the normal mouse fibroblast cell line L929 via MTT assays. Among them, compound **25** (Figure 30) demonstrated potent Eg5 inhibition, yielding an IC_50_ of 2.54 μM in the Malachite Green assay and 4.03 μM in the steady-state ATPase assay, alongside moderate cytotoxicity against MDA-MB-231 cells. An inhibitory effect was compared to a control—*S*-trityl-L-cysteine (IC_50_ value of 2.50 μM). The ATPase activity is directly linked to motor function during mitotic spindle formation. Inhibitors that reduce ATPase activity typically impair the ability of Eg5 to generate force and assemble the spindle, leading to mitotic arrest and apoptosis.

Molecular docking results revealed that compound **25** forms key hydrogen bonds at the allosteric site of the Eg5 protein, specifically interacting with the Lys220 residue at a bond length of 1.82 Å. Additionally, a hydroxyl group participates in a hydrogen bond with Lys216 (a bond length of 1.87 Å). The quinoline ring also engages in π–π stacking with Arg221, underscoring the critical role of this heterocycle in driving binding affinity and establishing the correct orientation within the allosteric pocket of the Eg5 protein (Figure 30).

Recently, Peer and coworkers evaluated novel quinoline-containing conjugates against selected cancer cell lines (DU-145, LNCaP, Hep-G2, MCF-7, MDA-MB-231, and MDA-MB-453) [140]. Screening of the carboxamide hybrid series revealed that *N*-(4-((6-methoxyquinolin-8-yl)amino)pentyl)-1*H*-indole-2-carboxamide (**26**) (Figure 31) possessed the highest cancer-selective anti-proliferative efficacy, surpassing 8-aminoquinoline-based antimalarial—primaquine (PQ). Specifically, hybrid **26** was roughly 1.7-fold less active on MDA-MB-453 cells than the parent reference compound (IC_50_ = 23.83 µM vs. IC_50_ = 13.72 µM). On the contrary, the LNCaP prostate cancer line exhibited the highest sensitivity to compound **26** (IC_50_ = 11.9 µM), with a response profile correlated with PQ (IC_50_ = 29.5 µM). Importantly, this dual-pharmacophore design—linked quinoline–indole scaffold—yielded superior cytotoxic outcomes compared to the free combination of individual indole carboxylic acid and primaquine therapies.

Furthermore, to determine the pro-apoptotic mechanism of quinoline–indole hybrid **26**, the effect on cell cycle distribution was investigated. Disruption of cell cycle checkpoints, which normally trigger DNA repair or execute apoptosis upon critical damage, is a hallmark of uncontrolled proliferation in cancer. Therefore, enforcing cell cycle arrest remains a primary objective in reversing chemoresistance. After exposure to the quinoline–indole derivative **26**, LNCaP cells accumulated in the S and G2/M phases in a dose-dependent manner. At the maximum concentration tested, S-phase arrest was the predominant cytostatic effect, while G2/M percentages returned to the control, suggesting a concentration-dependent mechanism.

### 2.6. DNA-Targeting and Repair Agents

Human topoisomerase I (Topo-I)—a key enzyme involved in the regulation of DNA topology during replication, transcription, and DNA repair—has emerged as a therapeutic target. Its inhibition can cause DNA damage and consequently impair cancer cell proliferation [141]. The introduction of substituents such as bromine, methoxyl, hydroxyl, or a nitro group can alter the electronic and hydrophobic properties of quinoline derivatives, thereby enhancing their interaction with biological targets and potentially improving their inhibitory activity. Instead of a generalized effect, the strategic positioning of these substituents on the quinoline core is critical for modulating Topo-I inhibition. Specifically, these modifications alter the planarity and electron density of the scaffold, which directly enhances π–π stacking interactions required for DNA intercalation and stabilizes the transient Topo-I–DNA cleavable complex.

Çakmak et al. developed highly brominated quinolines and tested their efficacy against rat glioblastoma C6, human cervical cancer HeLa, and human colorectal adenocarcinoma HT29 cell lines [142].

Experimental biological assays combined with molecular dynamics simulations demonstrated that 3,5,6,7-tetrabromo-8-methoxyquinoline (**27a**) and 5,7-dibromo-3,6-dimethoxy-8-hydroxyquinoline (**27b**) (Figure 32) possess notably antiproliferative activity against the tested cancer cell lines (IC_50_ = 12.8–32.3 μM) when compared to the reference drug 5-fluorouracil (IC_50_ = 240.4–258.3 μM), as well as inhibitory effects on the human Topo-I enzyme. Among the compounds investigated, derivative **27b** exhibited the most pronounced biological activity. Importantly, both compounds showed lower cytotoxicity then commonly used chemotherapeutic 5-fluorouracil in LDF assay, highlighting their potential as safer conditions for further anticancer drug development. Alongside their molecular dynamics simulations, the authors performed an in vitro human Topo-I inhibition assay to experimentally establish the molecular target. The biochemical screening confirmed that the highly brominated quinoline derivatives directly interfered with Topo-I activity, thereby validating the target interaction modes predicted by the computational models.

In view of cancer’s multifactorial nature, dual-acting agents are exceptionally effective therapeutic options [143]. One powerful example involves the design of quinoline-bridged hydroxamic acid derivatives by Joshi and coworkers intended to simultaneously inhibit human topoisomerase I/II (Topo-I/II) and histone deacetylases (HDACs) [144]. Among designed quinolines, the lead compound, *N*-hydroxy-6-((2-(4-methoxyphenyl)quinolin-6-yl)oxy)hexanamide (**28**) (Figure 33), demonstrated remarkable in vitro and in vivo efficacy by significantly reducing tumor growth and volume in an A549 lung cancer xenograft model. This potent anticancer profile is driven by multiple mechanisms, most notably through an atypical binding mode to HDAC1, alongside comprehensive molecular docking and drug metabolism and pharmacokinetics (DMPK) validations. As a result of its therapeutic significance, this work and its structural optimization strategies have been highlighted and extensively cited in a recent comprehensive review of promising quinoline derivatives as potential anticancer agents [58].

In 2025, Omidkhah et al. screened a novel series of 2-styrylquinoline derivatives for their antiproliferative potential against breast MCF-7, lung A549, and colon HCT116 malignancy models [145]. The structure–activity relationships indicated that introducing lipophilic groups at the C-6 and C-7 positions of the quinoline framework significantly influences their anticancer efficacy. Notably, most of these synthesized analogs demonstrated superior potency against breast (MCF-7) and lung (A549) carcinoma lines relative to colon cancer cells (HCT116). Among the library, compound **29** (Figure 34)—distinguished by dimethyl substitution at C-6 and C-7—emerged as the most effective candidate, yielding an IC_50_ value of 14.95 μM for MCF-7, IC_50_ = 5.7 μM within the A549 cell line, IC_50_ = 8.6 μM for HCT116, and IC_50_ = 32.96 μM against the normal L929 fibroblast cell line. Consequently, this lead compound was subjected to comprehensive biophysical assays to elucidate its exact DNA-binding properties. The formation of a distinct macromolecular assembly between compound **29** and calf thymus DNA (ctDNA) was initially signaled by an increase in resonance light scattering (RLS) intensity upon titrating the ligand. This association was corroborated by steady-state fluorescence quenching experiments.

Furthermore, assays with acridine orange (AO) and ethidium bromide (EB) confirmed that compound **29** competes for the same binding sites as classical DNA intercalators. Computational docking and molecular dynamics (MD) simulations demonstrated that the flat aromatic core of derivative **29** inserts neatly into the DNA base-pair stack. The system is fundamentally anchored by face-to-face π–π stacking (arene–arene) alignments with adjacent deoxyadenosine (DA *A*605, DA *A*606) and deoxythymidine (DT *B*620) residues (Figure 34). Concurrently, the specific hydrogen bonding channels predicted by the thermodynamics are fully visualized: the heterocyclic quinoline nitrogen serves as a hydrogen bond acceptor for the side chain of DT *B*619, while the terminal protonated amine on the flexible extension bridges with DA *A*605. Finally, the outer periphery of the diethylamino group and the terminal phenyl ring project outward into the solvent-exposed regions. Collectively, these integrated biophysical, spectroscopic, and in silico data confirm that compound **29** binds spontaneously to ctDNA via a mixed intercalation and electrostatic mode.

Seeking to overcome the limitations of current anticancer drugs, Kumar and coworkers developed a series of novel quinoline derivatives, from which (*E*)-6-chloro-*N*’-(4-ethoxybenzylidene)-2-(thiophen-2-yl)quinoline-4-carbohydrazide (**30**) shown in Figure 35 emerged as the lead compound [146]. This quinoline-based compound exhibited the most potent cytotoxicity against both human lung adenocarcinoma (A549) and murine Ehrlich Ascites Carcinoma (EAC) cell lines, with IC_50_ values of 4.26–4.78 µM (MTT assay), and 4.18–4.56 µM (Trypan blue assay), respectively. Molecular analyses revealed that **30** successfully triggers apoptotic pathways and suppresses angiogenesis by modulating key regulatory genes, including *BAX*, *BAD*, p53, and matrix metalloproteinases *MMP-2* and *9*. Phenotypically, its mechanism of action was characterized by marked nuclear damage, chromatin condensation, and disrupted cell membrane integrity. Importantly, these results were confirmed in vivo, demonstrating that treatment with **30** led to marked reductions in solid tumor volume and a notable extension of survival times. Taken together, these data positions this multi-target quinoline-based compound as a promising candidate for anticancer drug development.

Under physiological conditions, the BRCA2 and RAD51 proteins are key components of the homologous recombination (HR) DNA repair system. By using this system, the most dangerous genomic damage can be repaired without error, namely double-strand breaks (DSBs). When DNA damage occurs, BRCA2 physically binds to free RAD51 molecules. BRCA2 precisely transports and recruits RAD51 to the strand-breaking site, enabling it to form an active protein filament on single-stranded DNA. This filament searches the genome for an undamaged template and can initiate repair. Cancer cells with BRCA2 mutations are naturally impaired in HR repair, making them sensitive to PARP inhibitors (synthetic lethality). In many other aggressive cancers, such as pancreatic cancer, cells with a properly functioning BRCA2 gene (HR-proficient) can easily repair their DNA and escape therapy. Artificially blocking the contact between RAD51 and BRCA2 by using a small molecule can chemically turn off the HR system. This artificially places the cancer cell in a DNA repair-deficient state, dramatically sensitizing it to olaparib and leading to its death [147].

Starting from 2-(5-(2-carboxyphenyl)furan-2-yl)-6-methylquinoline-4-carboxylic acid Ferrandi and coworkers developed a novel analogue **31** (Figure 36), which effectively inhibits the RAD51-BRCA2 interaction and impairs homologous recombination [148]. In HR assays, sodium 6-methoxy-2-(5-phenylfuran-2-yl)quinoline-4-carboxylate (**31**) achieved 74% inhibition at 40 μM. The reference inhibitor CAM833 at a concentration of 50 μM displayed inhibition > 90%. Unlike CAM833, which showed only marginal synergy, quinoline derivative **31** in combination with olaparib exerted a significantly better balance of potency and solubility in pancreatic cancer models, representing an optimized RAD51–BRCA2 chemotype. The combination of compound **31** with olaparib in BxPC-3 spheroids demonstrated the induction of apoptosis by simultaneously blocking two DNA repair mechanisms. Sparing normal cells makes compound **31** a promising tool for further development of RAD51-BRCA2 inhibitors.

Molecular docking demonstrated that compound **31** adopts an inverted binding conformation within the RAD51 LFDE pocket compared to 2-(5-(2-carboxyphenyl)furan-2-yl)-6-methylquinoline-4-carboxylic acid. The LFDE pocket is a highly conserved, specialized binding groove located on the surface of the RAD51 protein’s ATPase domain. This flip allows the quinoline ring’s ionised carboxylic group to form hydrogen bonds with Arg250 and Arg247, while its unsubstituted phenyl ring secures π–π interactions with Tyr205 and an additional π-cation interaction with Arg252 (Figure 36).

### 2.7. Histone Deacetylases (HDACs), Cytoskeletal Dynamics and Protein Modifiers

Dysregulated epigenetic pathways and metabolic reprogramming are deeply intertwined hallmarks of cancer that directly drive multidrug resistance (MDR) [149]. While cytoplasmic HDAC6 sustains metabolic homeostasis by regulating the clearance of cytotoxic misfolded proteins via the aggresome pathway, nuclear Class I HDACs (HDAC1, 2, and 3) transcriptionally modulate key metabolic enzymes and suppressors of drug-efflux pumps [150,151]. Furthermore, alterations in microtubule dynamics often serve as a primary escape mechanism for resistant cells against antimitotic therapies. Consequently, targeting these distinct structural and epigenetic components represents a powerful strategy to disrupt tumor bioenergetics and reverse MDR. In this context, recent medicinal chemistry efforts have focused on developing both structural modulators and isoform-selective HDAC inhibitors to overcome recalcitrant tumor phenotypes. To disrupt the structural adaptations that drive chemotherapy resistance, targeting microtubule networks remains a highly effective strategy. Microtubules are dynamic cytoskeletal elements made up of α/β-tubulin heterodimers that are integral to mitotic spindle formation, intracellular transportation, and cell shape maintenance. Drugs that bind to the colchicine site of β-tubulin can disrupt microtubule polymerization, block mitosis, and induce cytotoxicity in cancer cells, bypassing many classic MDR phenotypes.

Using this approach, Kong and coworkers designed a novel series of 6-fluoro-*N*-(4-methoxyphenyl)-*N*-methyl-2-(trifluoromethyl)quinoline-4-amine derivatives containing alkyl chains of various lengths at the 3′-position of the phenyl ring, terminated with polar functional groups such as carboxyl, ester, amide, or hydroxamic acid [152]. These compounds were evaluated against PC3 prostate cancer, LNCaP prostate cancer, and A549 lung cancer cell lines using the MTT assay. Among them, 2-(5-((6-fluoro-2-(trifluoromethyl)quinolin-4-yl)(methyl)amino)-2-methoxyphenoxy)acetamide (**32**) (Figure 37) was found to exhibit the most potent antitumor activity, yielding an IC_50_ value of 1 nM against prostate adenocarcinoma LNCaP cells. Notably, its cytotoxic activity against this cell line was 250-fold greater than that of the reference drug colchicine (IC_50_ = 250 nM), highlighting its capability to overcome potential resistance barriers. Further mechanistic assays demonstrated that derivative **32** could efficiently inhibit microtubule polymerization by binding to the colchicine site and induce cellular apoptosis. These experimental results correlated well with molecular docking simulations, which identified the structural fitting and crucial interactions of **32** within the colchicine-binding pocket of tubulin (Figure 37).

Complementing microtubule disruption, regulating the degradation of misfolded proteins generated during therapeutic and metabolic stress offers another vital avenue to defeat MDR. Malignant cells frequently alter their metabolic networks to survive chemotherapy, utilizing specific histone deacetylase (HDAC) isoforms and cytoskeletal adaptations to tolerate therapeutic stress and evade apoptosis [153]. Histone deacetylase 6 (HDAC6) maintains cellular homeostasis by regulating critical cytoplasmic processes, including microtubule dynamics, protein degradation, and cell motility. Accumulating evidence indicates that its overexpression is implicated in tumorigenesis, driving oncogenic transformation, tumor cell survival, and metastatic progression across various malignancies. Consequently, dysregulated enzyme activity correlates strongly with poor clinical prognosis and diminished survival rates in cancer patients. This establishes HDAC6 as a high-priority therapeutic target for the development of novel anticancer interventions to sensitize resistant tumors [154].

Since histone deacetylase (HDAC)-mediated epigenetic abnormalities are critical drivers of oncogenesis and drug resistance, designing isoform-selective HDAC inhibitors has become a premier approach to enhance therapeutic efficacy while avoiding the systemic toxicities typical of pan-HDAC inhibition. To address this therapeutic target, Peng and collaborators developed the highly potent HDAC6 inhibitor **33** (Figure 38) with enhanced enzymatic potency, yielding an IC_50_ value of 11.57 nM alongside remarkable isoform selectivity [155].

It was revealed that **33** exerts broad-spectrum antiproliferative efficacy across an extensive panel of human malignancies, including HeLa, A549, HCT116, MDA-MB-231, MDA-MB-468, Jurkat, RPMI-8226, and Molt-4 cell lines. Western blot analysis verified the intracellular selectivity of **33**, evidenced by the robust hyperacetylation of α-tubulin with no visible cross-reactivity toward nuclear histone H3. Flow cytometry verified that the hybrid induces cell cycle arrest specifically at the G2/M phase, while simultaneously triggering programmed cell death (apoptosis) and impairing angiogenic signaling. Crucially, this therapeutic profile translated into robust in vivo antitumor efficacy, as **33** treatment significantly suppressed tumor progression in patient-derived xenograft (PDX) mouse models. Collectively, these findings strongly support the preclinical development of **33** as a multi-targeted anticancer candidate capable of disrupting metabolic networks and structural defenses.

While cytoplasmic HDAC6 inhibition targets proteostatic stress, modulating nuclear Class I HDACs offers a parallel approach to disrupt tumor cell transcriptomes, shut down metabolic adaptations, and silence drug-efflux mechanisms. Within this framework, Yu’s group designed a novel series of *N*-arylamide-quinoline derivatives as selective HDAC inhibitors [156]. Among the synthesized library, compound **34** (Figure 39) exerted selective suppression against HDAC1, 2, 3, and 10, remaining entirely inactive against the HDAC4–9 panel. This distinctive preference for nuclear Class I isoforms was further rationalized and confirmed through molecular docking models.

In vitro biological screening revealed that **34** possesses strong antiproliferative properties against multiple cancer types, including HL-60, CCRF-CEM, and HepG2 lines. Crucially, the quinoline-based compound **34** displayed negligible cytotoxicity toward normal hepatocytes, resulting in a significantly enhanced selectivity index. Further evaluation demonstrated that derivative **34** triggers a dose-dependent hyperacetylation of histone H3, downregulates phosphorylated Rb expression, and activates the caspase-8 pathway. Together, these molecular events culminate in G0/G1 phase cell cycle arrest and the induction of extrinsic apoptosis, bypassing the G2/M checks of conventional tubulin agents. In animal models, compound **34** efficiently suppressed tumor growth without causing noticeable systemic toxicity via both oral gavage and intraperitoneal injections, yielding a tumor growth inhibition (TGI) rate of 58.4% at a dose of 20 mg/kg. Collectively, these data establish the *N*-arylamide-quinoline derivative **34** as a compelling, selective nuclear HDAC inhibitor candidate with excellent biological properties and a clearly defined mode of action against resistant tumor lineages.

Computational docking of the lead candidate **34** into the active sites of HDAC1 (PDB ID: 5ICN) supported the observed biological data (Figure 39). The resulting binding poses showed that the carbonyl oxygen (C=O) of compound **34** coordinates directly with the catalytic Zn^2+^ ion, while its amide nitrogen (-CONH-) participates in a critical hydrogen bond with Gly149. The aniline ring of the quinoline derivative **34** establishes a π-sulfur interaction with Cys151 and forms conventional hydrogen bonds with His141 and His178. Within the rigid linker, the benzene ring engages in dual π–π stacking configurations with Phe205 and Phe150. Furthermore, the quinoline core anchors itself securely within the hydrophobic pocket via a network of π-sulfur, π-σ, π-alkyl, and π–π stacking interactions involving Cys100, Asp99, and Phe205. Notably, the overall docking topology of the clinically approved selective inhibitor chidamide closely mirrors the binding topology of quinoline **34**, reinforcing its potential as a highly specific clinical candidate.

Reprogrammed cancer cell proliferation involves requiring high levels of protein synthesis, including concurrent folding and processing. *N*-terminal methionine aminopeptidases (MetAPs) constitute a universally conserved family of metalloenzymes (metal-dependent enzymes) whose primary function is to cleave the initiating methionine (iMet) from newly synthesized peptide chains. This proteolytic cleavage is a fundamental and essential step in the maturation of proteins that regulate further posttranslational modifications crucial to their stability. In humans, this enzymatic mechanism is driven by two isoforms MetAP1 and MetAP2. Due to its essential role in endothelial cell proliferation and tumor angiogenesis, MetAP2 has attracted considerable interest as a valuable oncological target. There is also extensive evidence suggesting that MetAP2 is involved in accelerating cancer cell survival and resistance to chemotherapy, as demonstrated by the aberrant overexpression of MetAP2 in many solid tumors. It has been discovered that targeted inhibition of MetAP2 disrupts protein processing as well as dynamically activating the integrated stress response (ISR). As a result, tumor cells are deprived of amino acids. Finally, activation of the proapoptotic transcription factor ATF4 via non-canonical signaling mediated by mTORC1 leads to programmed cell death [157,158,159].

In 2026, Williams et al. investigated MetAP2 as a potential anticancer target after discovering that it is increased in cancer cells [160]. MetAP2 inhibition and cancer cell proliferation assays proved that the *O*-silylated nitroxoline derivatives—silyl(oxy)-5-nitroquinolines **35a**, **35b**, and **35c** (Figure 40) retained MetAP2 inhibitory activity and had broadly comparable antiproliferative potency to the parent nitroxoline, 8-nitroquinolin-5-ol, with the triisopropylsilyl derivative **35b** chosen as the lead candidate. Compounds **35a**, **35b**, and **35c** exhibited average MetAP2 inhibition values of 4.11 µM, 2.86 µM, and 3.43 µM, respectively.

Interestingly, Williams et al. [160] discovered that such compounds activate the integrated stress response, as demonstrated by ATF4 expression, and do so in a way that is associated with a non-canonical mTORC1 route. These studies have revealed a way to potentially prevent the pharmacokinetic problems of nitroxoline and demonstrate downstream stress signaling involving mTORC1 and ATF4. Future studies should focus on compound **35b** and similar silylic compounds to demonstrate cancer cell selectivity. Target-engagement studies should validate the MetAP2–mTORC1–ATF4 anticancer mechanism.

### 2.8. Tumor Microenvironment and Efflux-Mediated Resistance

To survive therapeutic stress, advanced tumors rely on nitric oxide synthase (NOS), an enzyme that produces nitric oxide (NO) to regulate tumor vascular tone, manage hypoxia adaptation, and drive immune suppression [161]. Concurrently, tumors protect themselves using NAD(P)H quinone dehydrogenase 1 (NQO1); although this antioxidant enzyme is cytoprotective, it can be exploited as a ‘trojan horse’ to bioactivate quinone-based prodrugs into lethal cytotoxic agents. NQO1 plays essential roles both in protecting normal cells from toxic effects of agents and oxidants and in promoting progression and development of chemoresistance in cancer cells [162]. Finally, to prevent the intracellular accumulation of these and other toxic threats, cancer cells overexpress the P-glycoprotein efflux pump (P-gp/ABCB1)—an ATP-binding cassette transporter that actively pumps chemotherapeutic agents out of the cell [163]. Understanding and targeting these interconnected mechanisms of detoxification, prodrug bioactivation, and active extrusion is essential to disrupt tumor fitness and overcome multidrug resistance in recalcitrant tumors.

Gliomas represent the largest group of primary brain tumors, originating from the supportive glial cells of the central nervous system (CNS) [164]. They account for roughly 30% of all cranial tumors and approximately 80% of malignant central nervous system malignancies. Tumor survival and angiogenesis are heavily dependent on chronic inflammation. By modulating the immune response, glioma cells construct an immunosuppressive microenvironment that directly facilitates tumor growth.

Under the influence of cytokines or microbial products, gliomas overexpress inducible nitric oxide synthase (iNOS), an enzyme that synthesizes nitric oxide (NO). As a versatile free radical, NO possesses contrasting pathological effects.

As part of our innate immunity, NO assists in clearance of pathogens, but excessive amounts can cause tissue injury and chronic inflammation. In gliomas, this dichotomy is particularly important: low NO concentrations can induce apoptosis while inflammatory microenvironments can increase tumor survival and mediate resistance to chemo- and radiotherapy. iNOS plays a significant role in oncogenesis, which depends heavily on the local cellular context and absolutely NO concentration, making targeted inhibition of iNOS a viable therapeutic option for metastatic gliomas [165,166].

In 2025, utilizing pharmacophore hybridization, Gallorini et al. discovered novel quinoline-based dual nNOS/iNOS inhibitor **36** (Figure 41) against glioma progression [167].

Compound **36** was evaluated as a selective neuronal nitric oxide synthase (nNOS) inhibitor using a fluorimetric L-citrulline assay, sparing enzyme located in the endothelial cells, which line the inside of blood vessels—endothelial nitric oxide synthase (eNOS) to maintain vascular homeostasis. Quinoline **36** demonstrated high potency (iNOS, IC_50_ = 2.9 μM and nNOS, IC_50_ = 0.1 μM in contrast to eNOS IC_50_ > 100 μM) and reasonable metabolic stability—73% remaining after 60 min in liver microsomes. This compound **36** exerted selective, time- and dose-dependent cytotoxicity in cancerous brain C6 glioma cells compared to normal brain astrocytes CTX TNA2, with viability dropping below 40% at 75 μM within 24 h. Enhanced anticancer potency of compound **36** may be linked to the dual inhibition of nNOS and iNOS, causing higher LDH release in glioma cells. Surprisingly, combining *N*-(2-(4-methylquinolin-2-yl)phenyl)acetimidamide (**36**) with reference temozolomide (TMZ)—a standard drug for aggressive brain tumors—resulted in lower efficacy than compound **36** alone. These findings suggest that designed quinoline-based derivative **36** may serve as a more effective standalone monotherapeutic agent against glioma cells.

NAD(P)H:quinone oxidoreductase 1 (NQO1) is frequently overexpressed in cancer cells. This enzyme catalyzes the reduction of quinolones, which may contribute to the generation of reactive oxygen species (ROS), oxidative DNA damage, and consequently, cell death. In a related study, Sokal et al. synthesized hybrids of 1,4-naphthoquinone with 8-hydroxyquinoline moiety **37a**, **37b**, and **37c** (Figure 42) and evaluated their biological potential by designation of enzymatic conversion rate of the NQO1 protein and in vitro against cancer cell lines with overexpression of gene encoding the NQO1 protein [168].

The analysis of the structure–activity relationship revealed that the biological activity is dependent on the type of substituents at the C2′ position of the quinoline ring, in the order as follows: methyl group **37b** > morpholine group **37c** > hydrogen atom **37a**. Accordingly, 2-(5-hydroxy-2-methylquinolin-8-yl)naphthalene-1,4-dione **37b** containing methyl group at the C2′ position was indicated as the NQO1 substitute with the highest efficiency, which was better than the reference substance β-lapachone (β-Lap) (1026 µmol NADPH/µmol NQO1/min vs. 985 µmol NADPH/µmol NQO1/min). This compound also exhibited the highest cytotoxicity against tested cancer cell lines including human lung carcinoma (non-small cell lung adenocarcinoma) A549, human breast adenocarcinoma MCF-7, human amelanotic melanoma that lacks the melanin pigment C-32, and human metastatic melanoma Colo-829 with IC_50_ values in the range of 0.88–19.56 µM, showing more efficiency than the reference drug cisplatin (IC_50_ = 2.92–20.91 µM). The experimental results were supported by docking simulations at the active site of the NQO1 protein.

Ligands **37a** and **37b** established key hydrogen bonds with tyrosine (Tyr128) and histidine (His161 and His194) residues. Specifically, the carbonyl groups at the C1 and C4 positions of the 1,4-naphthoquinone scaffold interacted with Tyr128 and His161, respectively (Figure 42). Additionally, the hydroxyl group of the quinoline core formed a weak hydrogen bond with histidine (His194). Within both ligand–protein complexes, the 1,4-naphthoquinone moiety engaged in hydrophobic interactions with tryptophan (Trp105), phenylalanine (Phe178), and the FAD cofactor. For the ligand **37c** complex, supplementary hydrophobic contacts were detected between the quinoline ring and two amino acid residues: glycine (Gly149) and phenylalanine (Phe232). In contrast, compound **37c**, which possesses a morpholine group, adopted a distinct orientation within the enzymatic binding pocket. For this ligand, only the carbonyl group at the C4 position of the 1,4-naphthoquinone core formed a hydrogen bond with tyrosine (Tyr126). Meanwhile, glycine (Gly193) and histidine (His194) stabilized the complex via hydrogen bonding with the morpholine moiety. Notably, the hydroxyl group of **37c** did not participate in any hydrogen bond formation.

Takács et al. investigated a series of hybrids as multifunctional anticancer agents capable of inducing tumor-cell death and overcoming P-gp-mediated multidrug resistance [169]. A synthesis of the quinine–chalcone compounds was performed to investigate their effects on A2058 melanoma, EBC-1 lung squamous cell carcinoma, and MCF-7 breast cancer cells. The tested compounds exhibited anticancer activity against at least one cancer cell line, with **38a** and **38b** (Figure 43) emerging as drug candidates. Compound **38a** showed the highest activity against EBC-1 cells (IC_50_ = 0.88 µM), whereas **38b** demonstrated the most balanced anticancer properties across all three cancer cell lines (IC_50_ = 1.60–2.17 µM). In EBC-1 cells, both compounds induced a concentration-dependent activation of the initiator caspase-8 and executioner caspases-3/7, ultimately resulting in pronounced apoptotic cell death. At a concentration of 25 µM, treatment with **38a** and **38b** enhanced the proportion of late-apoptotic and dead cells by 88.3% and 96.8%, respectively, and increased intracellular hydrogen peroxide levels. On the other hand, at lower concentration, they could induce apoptosis without increasing oxidative stress that may cause severe side effects. More importantly, the two hybrids were able to boost calcein retention inside cells by inhibiting P-gp activity without significantly lowering P-gp expression levels. These findings indicate that **38a** and **38b** limit P-gp function and induce caspase-dependent apoptosis, suggesting that these compounds may have potential as therapeutic agents for the treatment of drug-resistant cancers.

### 2.9. Apoptosis and ROS-Mediated Cell Death Pathways

By regulating mitochondrial outer membrane permeabilization (MOMP), BCL-2 (B-cell lymphoma 2) family proteins determine whether cytochrome C is released into the cytoplasm. Based on their function, these proteins are divided into three subgroups: multidomain proapoptotic proteins (e.g., BAX, BAK), BH3-only proapoptotic proteins (e.g., BAD, BID), and antiapoptotic proteins (e.g., BCL-2 and BCL-XL). Overexpression of BCL-2 promotes increased cancer cell invasiveness and tumor progression, promotes angiogenesis, and significantly influences cell response to chemotherapy, often leading to the development of drug resistance [170,171,172]. Contributing to this line of research [173], in 2025, Piškor and coworkers synthesized a series of quinoline derivatives, among which they identified a lead compound, (*Z*)-*N*’-hydroxy-4-(5-((quinolin-8-ylamino)methyl)isoxazol-3-yl)benzimidamide (**39**) (Figure 44). In antiproliferative assays against eight human cancer cell lines and noncancerous peripheral blood mononuclear cells (PBMCs), quinoline–amidoxime **39** demonstrated exceptional potency. Compound **39** displayed remarkable cytotoxicity, with IC_50_ values ranging from 2.1 µM to 4.7 µM against hematologic cancer cells (Z-138, DND-41, HL-60) and pancreatic adenocarcinoma (Capan-1), achieving excellent selectivity indices (SI) of up to 48. Furthermore, permeability and metabolic stability studies revealed that compound **39** possesses high membrane permeability alongside moderate clearance rates.

To elucidate its molecular mechanism of action, molecular docking studies were performed and validated by the redocking of Bcl-2 inhibitor—J1Q. The computational data confirmed that **39** effectively binds to the Bcl-2 protein, forming highly stable and energetically favorable complexes (Δ*G*_bind_ = −84.98 kcal/mol). Thermodynamic analysis indicated that the stabilization was predominantly driven by lipophilic energies and van der Waals interactions, with additional favorable contributions from coulombic energies. Notably, quinoline-based lead compound **39** maintained critical interactions with key residues, including π–π stacking with Phe153, hydrophobic interactions with Met115, and hydrogen bonding with Gln114 and Val133. Taken together, these results strongly support compound **39** as a candidate for further development and structural optimization as a selective, Bcl-2-targeted anticancer agent.

A caspase is a family of proteases that participate in several instances of programmed cell death. In apoptosis, intracellular contents are degraded without membrane rupture, preventing the triggering of an immune response. The most widespread apoptotic pathways are caspase dependent. Generally, caspase-3 is the most prevalent executor of caspase, but some cells display, although some cells also express caspase-7. Caspase-3 is frequently activated as a result of activating upstream caspase-8 and/or caspase-9 isoforms. Extrinsic apoptosis is primarily caused by caspase-8, while intrinsic apoptosis is mainly caused by caspase-9 [174].

Aligning with the well-established field of caspase biology, Bouria et al. designed novel hybrids composed of quinoline ring and thiazolo[3,2-*a*]benzimidazole-3(2*H*)one skeleton as caspase activators [175]. First, the compounds were characterized and evaluated for their cytotoxic effects on human gastric (AGS) and lung (A549) cancer cell lines. Among the series, 2-(2-chloro-5,8-dimethoxyquinolin-3-yl)benzo[4,5]imidazo[2,1-*b*]thiazol-3(2*H*)-one (**40a**) and 2-(2,5,8-trimethoxyquinolin-3-yl)benzo[4,5]imidazo[2,1-*b*]thiazol-3(2*H*)-one (**40b**) (Figure 45) showed the most prominent antiproliferative profiles, which prompted further investigation into cell death mechanisms.

Neither of the tested hybrids induced a release of lactate dehydrogenase (LDH), indicating that cell death may occur via an apoptotic pathway. Furthermore, enzyme activation assays showed that both derivatives selectively activated caspase-3 and caspase-8 while exerting no effect on caspase-9, suggesting that the extrinsic pathway may be involved. This apoptotic mechanism was supported by molecular docking simulations at the caspase-3 active site. The tested hybrids **40a** and **40b** displayed binding modes closely resembling those of the clinically approved anticancer drug topotecan (ΔG = −7.1 kcal/mol and −6.7 kcal/mol vs. −7.4 kcal/mol). A structure–activity relationship (SAR) analysis demonstrated that the superior molecular complexity and volume of compounds **40a** and **40b**—distinguished by the presence of two OCH_3_ groups on the quinoline core—were crucial for their high potency. Finally, these lead compounds demonstrated excellent drug-likeness parameters, highlighting their significant potential for future development as novel caspase activators. Detailed binding profiles of molecules **40a** and **40b** within caspase-3 (Figure 45) indicate that quinoline derivative **40a** displays multi-faceted interactions, featuring carbon-hydrogen bonds with Ser120, electrostatic stabilization with Arg207, and various alkyl or π-alkyl hydrophobic contacts with Cys163, Trp206, and His121. Similarly, analog **40b** interacts through a hydrogen bond with Ser209 complemented by distinct π–π interactions with Tyr204 and Trp206 (Figure 45). The binding architecture of topotecan is governed by hydrogen bonding with Phe250 and Asn208, together with a single π-alkyl interaction with Trp206.

Safavi et al. developed a series of quinolinvinyl-phenoxy-1,2,3-triazole-acetamide hybrids (Figure 46) as potential breast anticancer agents and evaluated their cytotoxicity against MCF-7 and triple-negative MDA-MB-231 breast cancer cells [176].

The structure–activity analysis revealed that the substitutions at the X and Y positions significantly influenced the cytotoxicity of the tested compounds against used cancer cell lines. The highest anticancer potency was observed for (*E*)-*N*-(4-chlorobenzyl)-2-(4-((4-(2-(quinolin-2-yl)vinyl)phenoxy)methyl)-1*H*-1,2,3-triazol-1-yl)acetamide (**41**: X = H, Y = 4-Cl) which exhibited IC_50_ values of 16.84 µM against MCF-7 cells, and 21.78 µM against MDA-MB-231 cells. Biochemical assays indicated that compound **41** induced apoptosis in MDA-MB-231 cells via caspase-3 activation. Moreover, the anticancer activity of **41** was confirmed using a three-dimensional spheroid model, in which an IC_50_ value of 25.67 µM was determined, further demonstrating its antiproliferative efficacy.

In 2026, Atahan and coworkers evaluated quinoline-based sulfonamide chalcone derivatives for antiproliferative, antimigratory, and apoptotic effects [177]. The synthesized compounds were evaluated against three human cancer cell lines: HCT116 (colorectal cancer), HepG2 (hepatoblastoma), and AGS (gastric adenocarcinoma) using the WST-1 assay. Cisplatin was utilized as the standard reference drug. Most of the tested derivatives exhibited moderate cytotoxicity, with IC_50_ values ranging between 18 and 39 μM. Among the synthesized series, compounds **42a** and **42b** (Figure 47) emerged as the most potent lead candidates, displaying significantly enhanced cytotoxicity across all tested cell lines. Notably, both lead compounds demonstrated a pronounced time-dependent efficacy. After 48 h of incubation, the IC_50_ values for **42a** decreased to 1.98 μM (HCT116), 9.82 μM (HepG2), and 7.01 μM (AGS). A similar time-dependent trend was observed for **42b**, which yielded IC_50_ values of 2.67 μM (HCT116), 11.13 μM (HepG2), and 8.96 μM (AGS). Remarkably, **42a** outperformed cisplatin in HCT116 cells by approximately six-fold (1.98 μM vs. 11.61 μM) and in AGS cells by over 2.5-fold (7.01 μM vs. 18.57 μM). It was found that the colorectal cancer line (HCT116) was the most sensitive, prompting its selection for further anti-migration and apoptosis investigations. Moreover, to determine the tumor-selective profile of the lead compounds, their cytotoxicity was evaluated against non-tumor human umbilical vein endothelial cells (HUVECs). At a concentration of 7.5 μM (48 h), both **42a** and **42b** reduced HUVEC viability by only 15–20%, whereas the same concentration induced a 60–65% reduction in HCT116 cell viability. To elucidate the mechanism underlying the cytotoxic effects of the lead compounds, caspase activity assays were performed in HCT116 cells. Both **42a** and **42b** significantly induced apoptosis. Specifically, treatment with **42a** at 7.5 μM resulted in approximately 3.5-, 2.9-, and 4.1-fold increase in caspase-3 activity, caspase-8, and caspase-9 activities, respectively, compared with untreated controls. Similarly, **42b** treatment induced a 2.5- to 3.0-fold increase in the activities of these caspases. The simultaneous activation of caspase-8 and caspase-9 suggests that both the extrinsic (death receptor-mediated) and intrinsic (mitochondrial-mediated) apoptotic pathway are involved, ultimately, contributing to caspase-3 activation and apoptotic cancer cell death.

To gain insights into the molecular mechanisms driving the observed apoptotic responses, molecular docking simulations were performed within the active site of human Bcl-2. Remarkably, several derivatives exhibited binding affinities comparable to or even exceeding those of the FDA-approved inhibitor venetoclax (−9.26 kcal/mol) and the control inhibitor (−11.70 kcal/mol), evaluated under identical redocking parameters. Concurrently, the highly active derivatives **42b** and **42a** demonstrated favorable binding profiles within the human Bcl-2 pocket. Their superior biological efficacy may be driven by targeted non-covalent interactions. These compounds establish hydrogen and halogen contact with arginine and tyrosine. Specifically, derivative **42b** establish critical hydrogen bonds with key residues—namely Arg66, Tyr67, and Arg105—at precise spatial distances of 2.97 Å, 2.08–3.05 Å, and 2.09 Å, respectively. Moreover, this compound forms π–π and π-alkyl interactions with Ala57, Val107, Ala108, and Leu96, complemented by extensive van der Waals, π-σ and halogen contacts with surrounding amino acid residues, including Phe63, Met74, and Glu111 (Figure 47). These insights highlight that effective Bcl-2 inhibition relies on a fine balance between overall binding affinity and well-positioned, specific molecular interactions.

## 3. Metal Complexes with Quinoline-Based Compounds

Finally, due to the presence of an aromatic nitrogen atom with a lone pair of electrons, the quinoline skeleton may serve as a neutral monodentate ligand for the formation of metal-coordinated compounds. A particular advantage of coordination compounds over their parent ligands may be their higher biological potency. Therefore, metal complexes may be especially effective in anticancer treatment, making them attractive as potential antitumor agents [178,179,180]. As anticancer agents, they modulate redox states, generate reactive oxygen species, and interfere with cellular signaling pathways. It is likely that continuous research into organometallic frameworks will lead to the development of precision oncology and the overcoming of drug resistance in the future [181,182,183]. The primary goal in the design of metal complexes is to find an alternative to common metal-based anticancer drugs such as cisplatin, against which cancer cells quickly develop resistance.

Calcium ions represent an attractive option for the development of novel anticancer heavy metals such as platinum, ruthenium, or iridium. These properties may facilitate the design of therapeutic strategies associated with reduced systemic toxicity. As calcium acts as a ubiquitous intracellular second messenger and plays a fundamental role in maintaining cellular homeostasis, disruption of Ca^2+^ signaling and homeostasis may provide an alternative strategy for inducing cancer deaths. In this context, the development of calcium-based complexes capable of intracellular delivery and controlled release of Ca^2+^ represents a particularly promising approach. A rapid increase in intracellular Ca^2+^ concentration can disturb calcium homeostasis and promote excessive Ca^2+^ accumulation within mitochondria, resulting in impairment of mitochondrial function through several mechanisms, such as enhanced generation of reactive oxygen species (ROS), increased oxidative stress, and disruption of mitochondrial membrane potential which impairs the vital functions of organelles [184,185]. However, research into calcium-based anticancer coordination complexes remains limited.

In 2026, Huang and coworkers designed and synthesized six binuclear calcium(II)-based complexes with the general formula [Ca_2_(*μ*_2_-O)_2_(QMx)_4_(QHy)_2_], incorporating halogenated 8-hydroxyquinolines (QMx) including 5,7-dibromo-8-hydroxyquinoline, 5,7-dichloro-8-quinolinol, 5,7-diiodo-8-hydroxyquinoline, and clioquinol, together with auxiliary bathophenanthroline and 1,10-phenanthroline (QHy) **43a**–**43f** (Figure 48) [186]. Cytotoxicity studies reveal that all Ca-complexes exhibit significant activity against cisplatin-resistant ovarian cancer cells cis-SK3 (SK-OV-3/DDP) and human-sensitive ovarian cancer cells SK-OV-3 used as a baseline comparison for cancer toxicity. To assess toxicity to healthy tissue and calculate the selectivity index, normal, non-tumorigenic human liver cells HL-7702 were used.

Notably, all complexes exhibited higher antiproliferative activity against cis-SK3 cells than the corresponding free ligands. In particular, Ca(II)-chelates: **43a** and **43b** containing 5,7-dibromo-8-hydroxyquinoline or 5,7-dichloro-8-quinolinol, and bathophenanthroline displayed the highest cytotoxic potency against cis-SK3, reaching IC_50_ values of 3.59 μM and 2.73 μM, respectively. Among the tested complexes, **43b** exhibited the most pronounced anticancer activity, with an IC_50_ value of 2.73 μM which was approximately 18-fold lower than that of cis-DDP (IC_50_ > 50 μM); **43b** also showed greater cytotoxic activity than **43a**. Furthermore, both **43a** and **43b** were shown to induce apoptosis as a primary mechanism of cell death in cis-SK3 cancer cells reaching 45.77% and 49.65%, respectively, compared with untreated controls. The compound **43b** was found to be more effective than the complex **43a**. The enhanced activity may be associated with the presence of 5,7-dichloro-8-quinolinol and bathophenanthroline as ligands.

Cytotoxic effects were accompanied by an increase in intracellular Ca^2+^ levels of up to 60–65%, suggesting that the disruption of calcium homeostasis may contribute to their pro-apoptotic activity. Treatment of cis-SK3 cells with **43a** and **43b** at their respective IC_50_ concentrations also increased ROS levels by 51% and 56%, respectively. Consistent with these findings, **43a** and **43b** were shown to induce mitophagy in cis-SK3 cells. Treatment significantly increased the expression of proteins such as LC3B II/I, Beclin1, PINK1, and Parkin, while decreasing the levels of the mitophagy-related biomarkers P62 and FUNDC1. Collectively, these results suggest that mitochondrial damage, oxidative stress, disruption of Ca^2+^ homeostasis and mitophagy activation are closely associated with the anticancer effects of **43a** and **43b**. Importantly, the coordination compounds **43a** and **43b** displayed cancer-selective cytotoxicity, while showing minimal toxicity toward human normal liver cell line HL-7702 (IC_50_ > 50 μM). Their selectivity index factor (SIF > 6.2) was substantially higher than that of *cis*-DDP (SIF < 0.3), suggesting a potentially improved therapeutic window.

It is noteworthy that metals such as nickel may also be valuable for the development of anticancer drugs [187,188,189,190]. In 2025, Naskar and coworkers designed a novel amino-azo–quinoline ligand (HL) **44** which was utilized to synthesize two mononuclear complexes of type [Ni(HL)(OAc)] **45a** and [Ni(HL)Cl] **45b** (Figure 49) [191].

It has been demonstrated that the free ligand 2-(quinolin-8-yldiazenyl)aniline (**44**) and particularly its Ni(II)-complex [Ni(HL)Cl] **45a** exhibit promising anticancer activity against human cervical cancer (Hela) cells. In the MTT assay, ligand **44** and complex **45a** induce approximately 65% cell death at a concentration of 10 µM. Both ligand **44** and complex **45a** also inhibited Hela migration, reducing the migration rate to 4.363 µm/h and 2.155 µm/h, respectively, compared with 5.148 µm/h in the control. Moreover, complex **45a** enhanced the intracellular ROS generation in the HeLa cells, suggesting that the anticancer effects of the investigated compounds may involve a ROS-dependent mechanism. Furthermore, the docking results revealed a strong binding affinity of Ni(II) complex **45a** toward DNA compared with the free ligand **44**. The square-planar geometry of complex **45a**, together with the presence of a labile chloride ligand, may facilitate its interaction with DNA and contribute to its enhanced DNA-binding properties.

A remarkable example of novel zinc(II) complex based on a quinolone-thiazolyl-hydrazone scaffold has been presented by Maciejewska and collaborators [192]. Octahedral complex **46** [Zn(HL)_2_](NO_3_)_2_ (Figure 50) was synthesized starting from *N*,*N*,*N*-tridentate ligand: 2-(2-(quinolin-8-ylmethylene)hydrazinyl)-4-(*p*-tolyl)thiazole and zinc(II) nitrate in methanol and further evaluated against human cancer cell lines: lung adenocarcinoma A-549 and colorectal carcinoma HCT-116.

The in vitro results revealed that zinc-complex exhibits selective activity specifically sparing non-tumorigenic human embryonic kidney HEK-293 cells. Furthermore, the cycle distribution and DNA synthesis in HCT-116 cells were assessed using flow cytometry. It was found that upon treatment with complex **46**, a marked and concentration-dependent accumulation of cells in the S phase was observed, accompanied by a corresponding decrease in the G0/G1 population. This suggests that complex interferes with DNA replication and induces S-phase arrest. After complex treatment, the proportion of S-phase cells increased from approximately 25% to nearly 46%. It was demonstrated that the compound produced an increase of over 60% in the total apoptotic fraction at a concentration of 25 μM, which suggests that apoptosis is a major mode of cell death. Zinc(II) complex **46** triggers significant DNA damage as indicated by increased expression of the phosphorylated form of histone H2AX (p-γH2AX). In addition, mitochondrial depolarization, caspase-3/7 activation, and phosphatidylserine externalization were seen, confirming that intrinsic apoptosis occurred. The complex **46** exerted tumor-suppressive activity in the chick chorioallantoic membrane CAM assay without overt toxicity. Moreover, it was found that the zinc complex had improved efficacy and selectivity over the parent free ligand which highlights its potential as a promising lead compound for further preclinical development.

Among the quinoline-derived ligands, 8-hydroxyquinoline (8-HQ) has attracted a great deal of interest due to its potent metal recognition properties and coordination properties [193]. The antiproliferative properties of these metal-based compounds have been well documented [194]. Currently, such potential antitumor agents incorporating 8-HQ mainly include complexes with platinum [195,196,197], lanthanum(III) [198,199,200], cobalt(II/III), nickel(II), copper(II), and zinc(II) [201,202,203,204,205], along with rhodium(III) [206], iridium(III) [207,208], and ruthenium (II/III) [209,210,211,212].

A significant contribution to this line of research was made by Khalifa and colleagues, who evaluated the anticancer efficacy of the synthesized bis(quinolin-8-olato)bis(triphenylphosphine)ruthenium(II) Ru(quin)_2_ **47** (Figure 51) starting from tris(triphenylphosphine)ruthenium(II) dichloride and quinolin-8-ol [213]. In 2025, this ruthenium(II) complex **47** was tested against ER-positive derived from infiltrating ductal carcinoma (T47D) and triple-negative (MDA-MB-231) breast cancer cell lines [214].

After 72 h of exposure, **47** induced dose-dependent cytotoxicity in T47D and MDA-MB-231 breast cancer cells, with IC_50_ values of 48.3 μM, and 45.5 μM, respectively. Treatment also promoted apoptosis, as evidenced by increased histone release and elevated caspase-3 activity. Moreover, **47** upregulated the pro-apoptotic *BAX* gene, while downregulating Aurora kinase B (*AURKB*), a key regulator of mitosis and cell survival. Collectively, these findings indicate that **47** exerts pro-apoptotic activity in both ER+ and TNBC cells.

Sivolap et al. [215] reported potent anticancer activity of the Ag(I) complex **48** [AgNO_3_L]_2_ shown in Figure 52 against several cancer cell lines. The complex exhibited IC_50_ values ranging from 6.39 µM to 8.25 µM against HeLa, A549, and MCF-7 cells, while substantially higher cytotoxic activity was observed against SH-SY5Y neuroblastoma cells (IC_50_ = 0.75 µM) [215]. The complex was more active than cisplatin in all tested cell lines, showing approximately 19-fold higher activity against SH-SY5Y cells. In contrast, the free ligand was inactive up to 100 µM, highlighting the importance of Ag(I) coordination for cytotoxic activity. However, **48** also displayed considerable toxicity toward non-cancerous HEK-293 cells (IC_50_ = 5.28 µM), resulting in limited selectivity toward investigated cancer cell lines. Notably, a selectivity index of 7.04 for SH-SY5Y cells indicated preferential activity against neuroblastoma cells. These findings identify **48** as a promising lead compound for further development.

In a recent study, Zhao et al. synthesized three stable hafnium(IV) complexes, namely **49a**, **49b**, and **49c** (Figure 53), derived from 8-hydroxyquinoline (HL_1_), 2-methyl-8-hydroxyquinoline (HL_2_), and 5-chloro-8-hydroxyquinoline (HL_3_) and evaluated for their cytotoxic effects on HepG2 liver, HeLa S3 cervical, and PC9 lung cancer cells after 48 h of exposure [216].

The unsubstituted complex **49a** [Hf^IV^(L_1_)_4_] (R^1^ = R^2^ = H) was the most potent with IC_50_ values of 0.84 μM, 5.12 μM, and 2.61 μM against HepG2, HeLa S3, and PC9 cells, respectively. Notably, its activity against HepG2 cells was approximately 16-fold higher than that of cisplatin (IC_50_ = 13.82 μM), while it was also more active against PC9 cells and showed comparable activity against HeLa S3 cells. The 5-chloro derivative **49c** [Hf^IV^(L_3_)_4_] (R^1^ = H, R^2^ = Cl) retained substantial cytotoxicity (IC_50_ = 1.90–8.89 μM), although chlorine introduction did not improve activity relative to **49a** (R^1^ = R^2^ = H). In contrast, the 2-methyl derivative **49b** [Hf^IV^(L_2_)_4_] (R^1^ = CH_3_, R^2^ = H) was active only against PC9 cells (IC_50_ of 5.57 μM), suggesting that the steric hindrance introduced by the methyl group at a position C2 of quinoline may impair activity. Importantly, none of the complexes showed quantificable IC_50_ values in non-tumorigenic murine AML12 cells indicating cancer cell selectivity. Mechanistic studies of the most active complex **49a** [Hf^IV^(L_1_)_4_] (R^1^ = R^2^ = H) in the HepG2 cells revealed G2/M cell cycle arrest and induction of predominantly early apoptosis, accompanied by increased caspase-3 and Bax, and decreased Bcl-2 expression.

It is worth noting that this hafnium complex offers a distinct advantage by selectively inducing early apoptosis, compared to the previously studied zirconium complex with greater cytotoxicity, cellular uptake, ROS generation, mitochondrial impairment, and antioxidant enzyme inhibition. The authors claimed that present hafnium complex **49a** shows great promise as potent liver cancer therapeutics due to their high efficacy and favorable selectivity profiles.

Head and neck squamous cell carcinoma (HNSCC) is commonly associated with tobacco use, alcohol consumption, and human papillomavirus infection, while chronic inflammation contributes to its development and progression through activation of the IκB Kinase beta/Nuclear Factor kappa B—IKKβ/NF-κB pathway [217,218,219,220]. The activated p65 subunit of NF-κB stimulates the expression of inflammatory cytokines such as IL-6 and IL-8, which promote cancer cell proliferation, angiogenesis, epithelial–mesenchymal transition, migration, metastasis, and treatment resistance [219,221,222,223,224,225,226]. Simultaneous inhibition of NF-κB signaling and its downstream cytokines may therefore provide an effective therapeutic strategy for HNSCC.

In the investigated Ru(II)-complex **50** (Figure 54) by Vasela et al., the 8-hydroxyquinoline moiety provides a strong metal-binding site, while the chalcone and 3,4,5-trimethoxyphenyl groups contribute potential anticancer and anti-inflammatory properties [227]. Their incorporation into a *p*-cymene–Ru(II) complex produced a multifunctional structure synthesized to affect both cancer cells and inflammation-associated signaling pathways.

In addition to its short-term cytotoxicity, complex **50** markedly inhibited clonogenic growth and migration of HNSCC cells. Colony-forming efficiency was reduced to 15–24%, while CAL-27 cells showed only 5% wound closure after 55 h compared with 83% in untreated controls. These effects were associated with inhibition of the IKKβ/NF-κB pathway. Complex **50** suppressed NF-κB activation by approximately 70% at 20 µM and strongly impaired p65 binding to its DNA response sequence, accompanied by reduced IL-6 and IL-8 levels. Microscale thermophoresis indicated direct interactions with IL-6 and IL-8 (K*_D_* = 24.7 μM and 11.4 μM, respectively), while molecular docking supported favorable binding to IL-6, NF-κB, and IL-8. Overall, complex **50** appears to exert anticancer effects by simultaneous suppressing HNSCC cell growth and migration and modulating pro-inflammatory NF-κB/IL-6/IL-8 signaling. However, further target engagement, mechanistic as well as in vivo studies are required to validate its molecular targets and therapeutic potential.

## 4. Conclusions

Quinoline-containing derivatives and their metal complexes represent a versatile structural platform in modern anticancer drug design, bridging complex cellular mechanisms from cell-surface receptors to epigenetic modulation. However, moving beyond a general summary of their multi-targeting capabilities requires an integrated critique of their current experimental standing, structural advantages, and translational limitations based on the thematic core of this review. While the quinoline moiety demonstrates a broad spectrum of biological activities, the strength of experimental evidence varies significantly across the reviewed mechanisms.

Strong experimental validation exists for quinoline-based compounds in intracellular signaling cascades, cell cycle regulation and mitotic arrest, and DNA-targeting agents. For these pathways, cellular assays are consistently backed by enzymatic data and high-resolution structural studies. In contrast, the roles of quinolines in DNA methylation and chromatin architecture, as well as cytoskeletal dynamics and protein modifiers, often rely on phenotypic cellular observations rather than direct biophysical binding assays. For these targets, comprehensive structural biology and direct binding parameters remain limited and require more rigorous confirmation.

From a structural perspective, specific quinoline-based chemotypes appear to play a significant role in anticancer efficacy. Substituted 4-aminoquinoline and 8-hydroxyquinoline scaffolds remain exceptional due to their dual-action potential. They act as chelating ligands and pharmacophores for intracellular signaling cascades. Stable quinoline–metal complexes, particularly those with Ru and Hf, consistently display high antiproliferative efficacy, enhanced induction of apoptosis, and ROS-mediated cell death pathways. Merging the quinoline ring with specific structural motifs that selectively alter the tumor microenvironment and efflux-mediated resistance constitutes the most viable strategy for creating next-generation chemotherapeutics.

Despite the rich chemistry and structural diversity of synthetic quinoline-based compounds, several critical gaps may delay their translation into medical treatments. In a few cases, the exact molecular targets that trigger apoptosis or mitotic arrest in complex phenotypic screens remain insufficiently defined. Moreover, a lack of comprehensive in vivo pharmacokinetic and pharmacodynamic data limits progress. Consequently, many highly potent in vitro candidates are not routinely progressed to animal models. Systematic assessments of toxicity and tissue-specific accumulation remain rarely mentioned in recent studies. To overcome these barriers, future efforts must integrate advanced computational modeling, quantitative structure–activity relationship (QSAR) approaches, and targeted drug formulation strategies to optimize bioavailability, reduce toxicity, and enhance target selectivity.

This review summarizes structurally diverse synthetic derivatives reported over the past two years, highlighting their in vitro anticancer potential and effects on selected molecular pathways. Consequently, this work does not provide a deep evaluation of the long-term clinical safety of the highlighted quinolines and their metal complexes. It is important to emphasize that quinolines are still a privileged structural element in modern drug design, despite these ongoing challenges. A review of some valuable and promising quinoline-based compounds and their metal complexes is presented in Table 1 and Table 2.

## Figures and Tables

**Figure 1:**
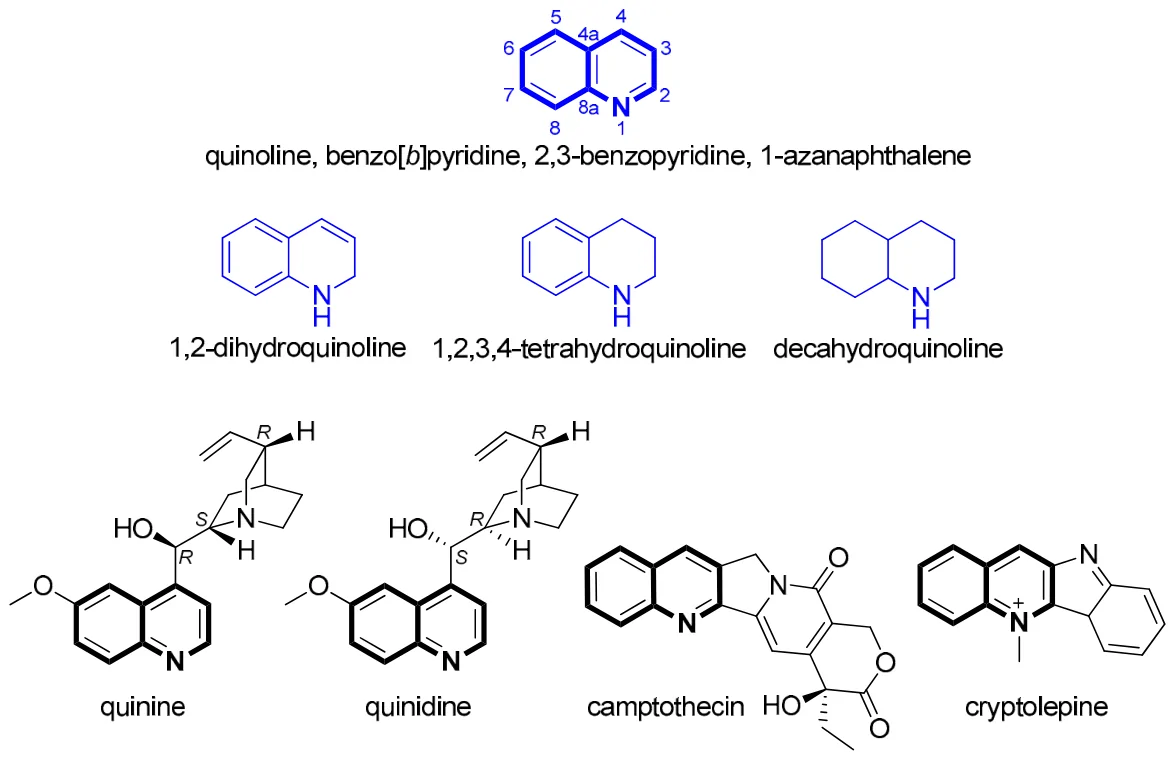
Chemical structures of quinoline (benzo[*b*]pyridine, 2,3-benzopyridine, 1-azanaphthalene), its reduced forms (1,2-dihydroquinoline, 1,2,3,4-tetrahydroquinoline, and decahydroquinoline) and natural alkaloids—quinine, quinidine, camptothecin, and cryptolepine.

**Figure 2:**
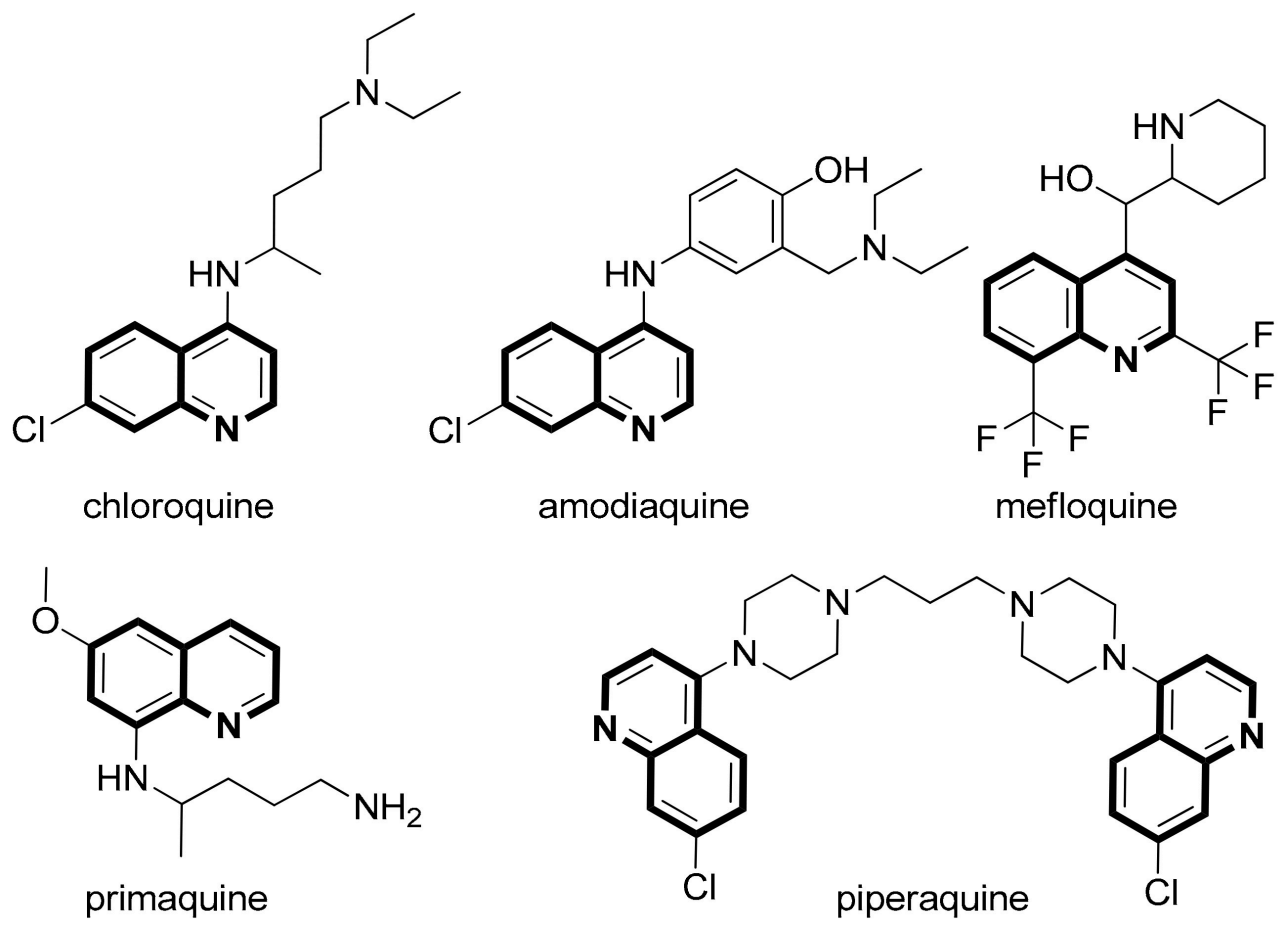
Structures of representative quinoline-based antimalarial drugs. Chloroquine (4-aminoquinoline class, FDA approved in 1949); amodiaquine (4-aminoquinoline class, introduced/approved in 1950); mefloquine (quinoline-methanol class, FDA approved in 1989); primaquine (8-aminoquinoline class, FDA approved in 1952); and piperaquine (bisquinoline class, synthesized in the 1960s, EMA approved in combination therapies in 2011).

**Figure 3:**
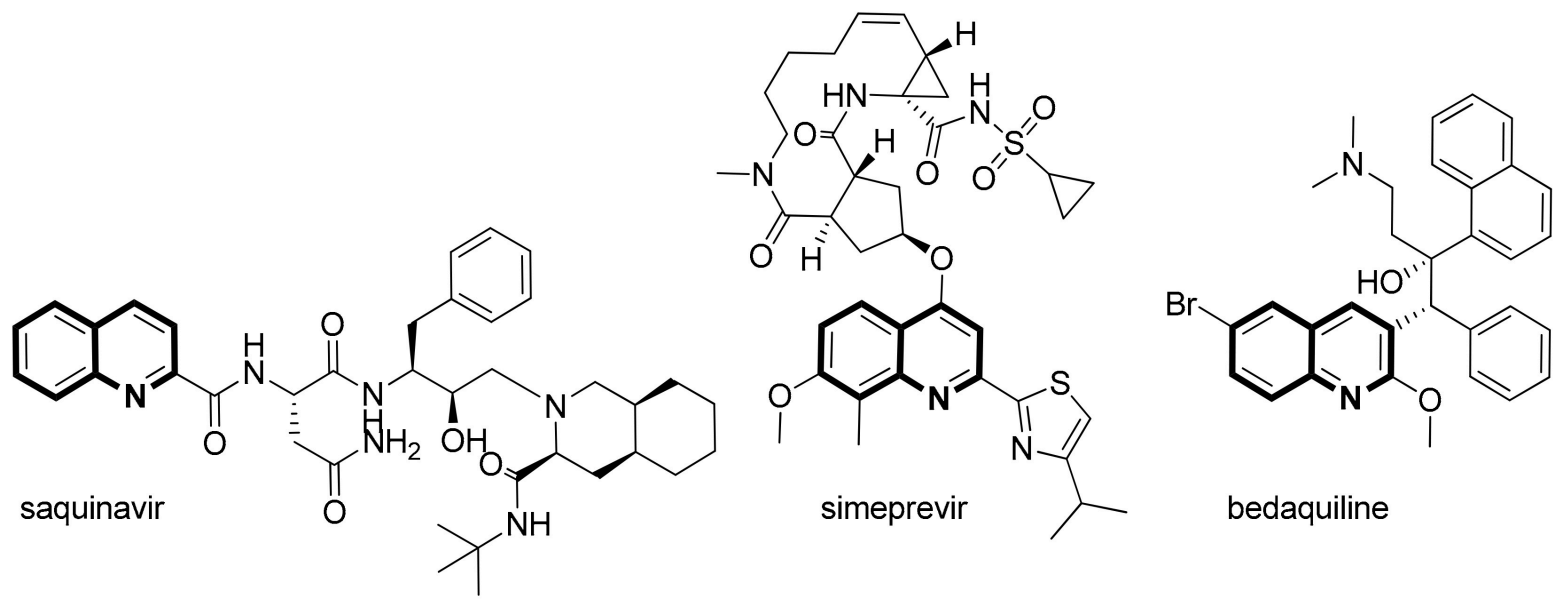
Structures of approved quinoline-containing antiviral and antibacterial drugs. Saquinavir (HIV protease inhibitor class, FDA approved in 1995); simeprevir (hepatitis C virus NS3/4A protease inhibitor class, FDA approved in 2013); and bedaquiline (diarylquinoline antituberculosis class, FDA approved in 2012).

**Figure 4:**
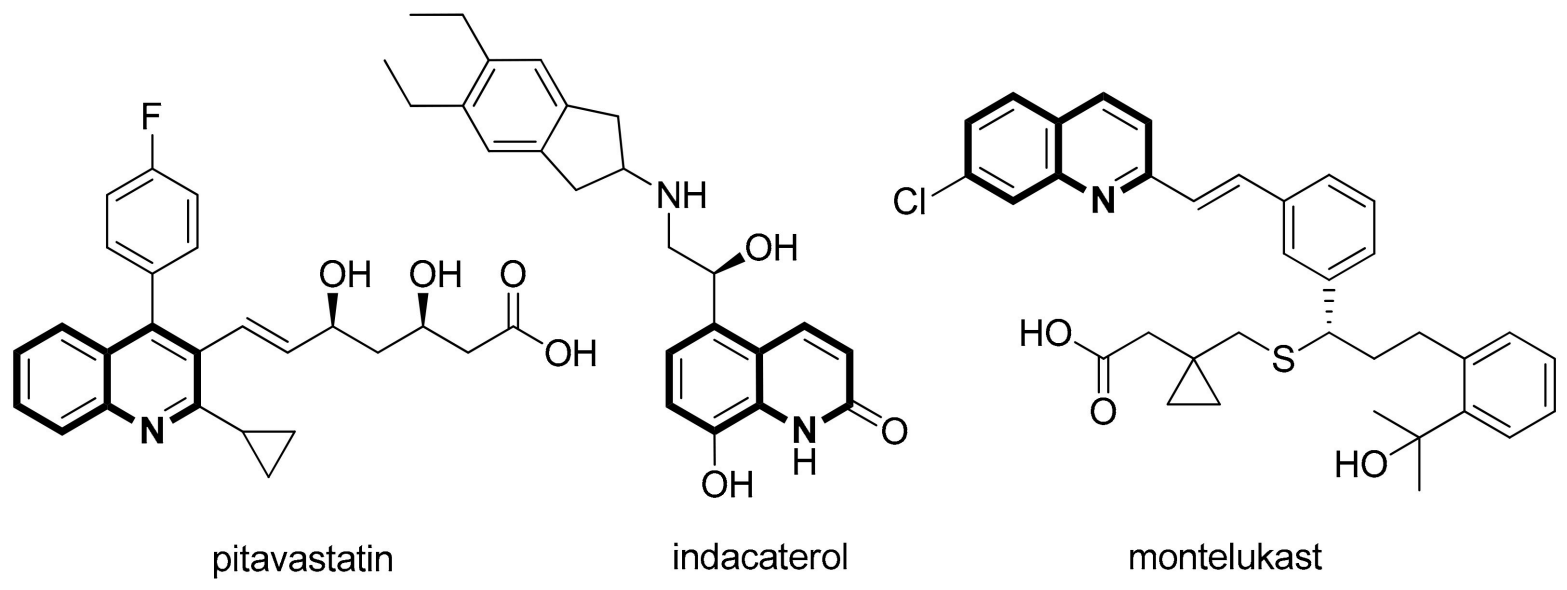
Structures of approved quinoline-containing cardiovascular and respiratory drugs. Pitavastatin (HMG-CoA reductase inhibitor or statin class, FDA approved in 2009); indacaterol (ultra-long-acting β_2_-adrenergic agonist class, FDA approved in 2011); and montelukast (leukotriene receptor antagonist class, FDA approved in 1998).

**Figure 5:**
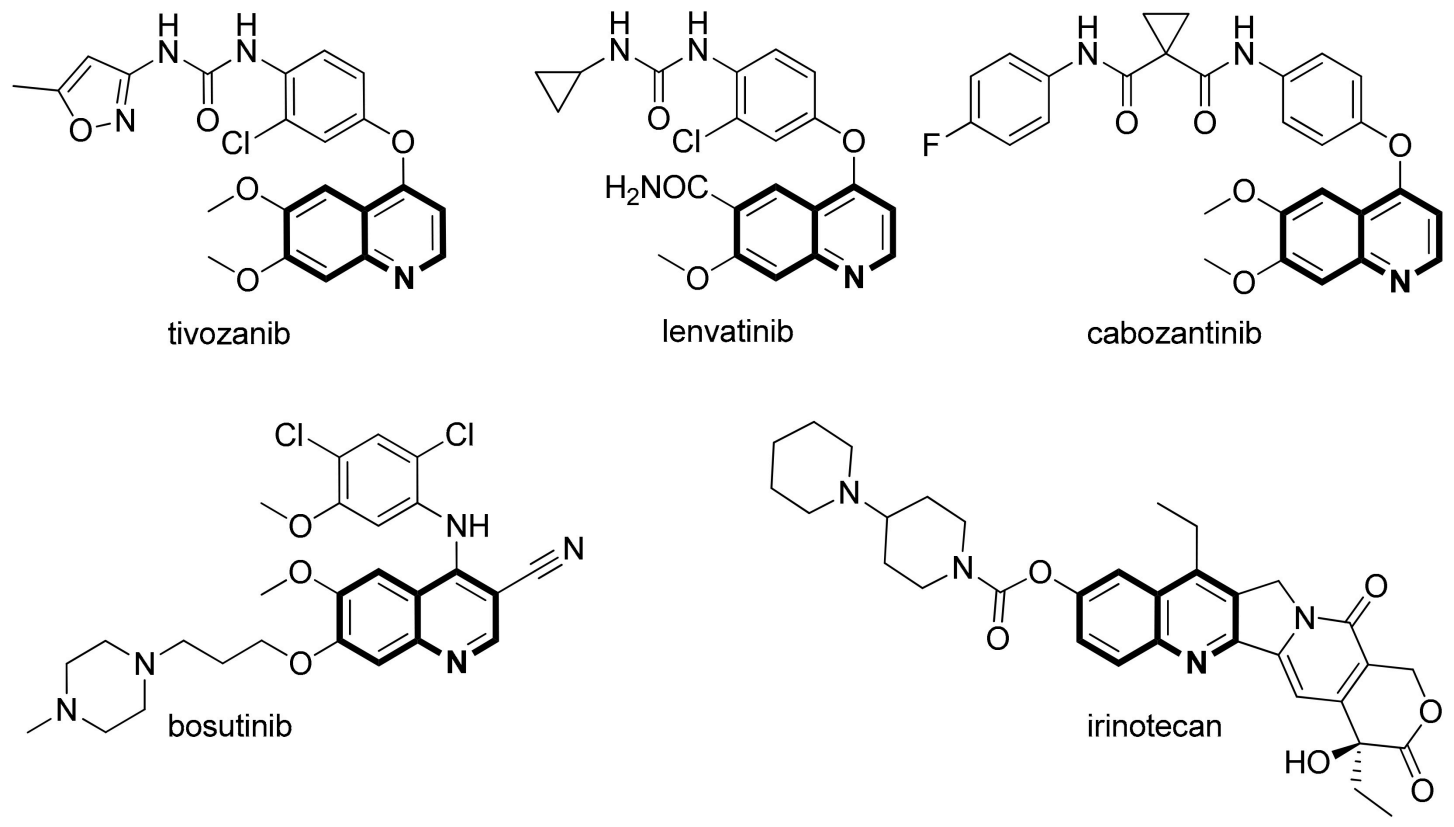
Structures of approved quinoline-based anticancer drugs. Tivozanib (multi-targeted receptor tyrosine kinase inhibitor focusing on VEGFR-1/2/3, FDA approved in 2021); lenvatinib (multiple receptor tyrosine kinase inhibitor targeting VEGFR-1/2/3, FGFR-1/4, PDGFR-α, KIT, and RET, FDA approved in 2015); cabozantinib (tyrosine kinase inhibitor targeting MET, VEGFR-2, and AXL, FDA approved in 2012); bosutinib (dual Src/Abl tyrosine kinase inhibitor, FDA approved in 2012); and irinotecan (topoisomerase I inhibitor class, a semisynthetic derivative of the quinoline alkaloid camptothecin, FDA approved in 1996).

**Figure 6:**
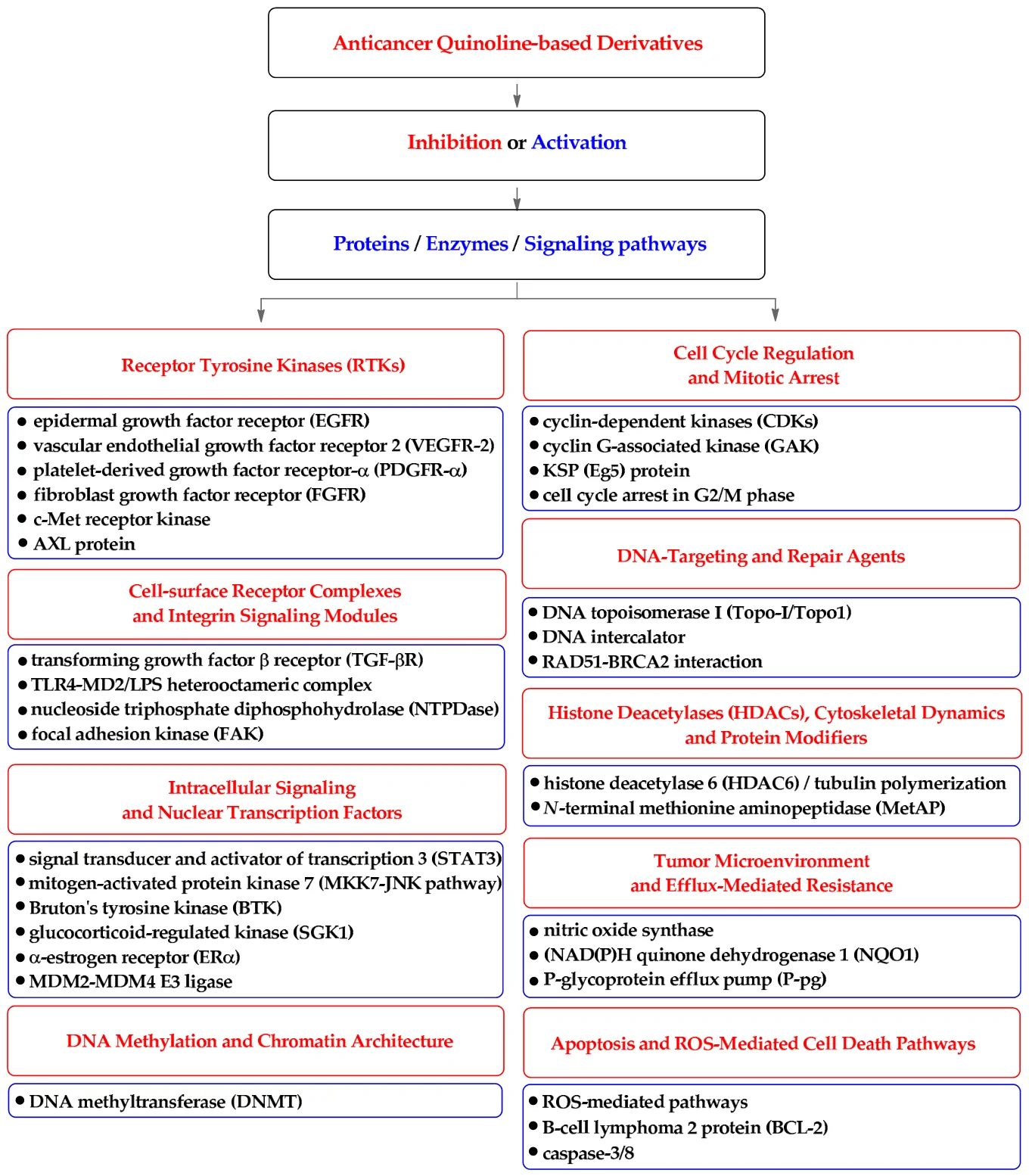
Targets of the anticancer synthetic quinoline-based compounds.

**Figure 7:**
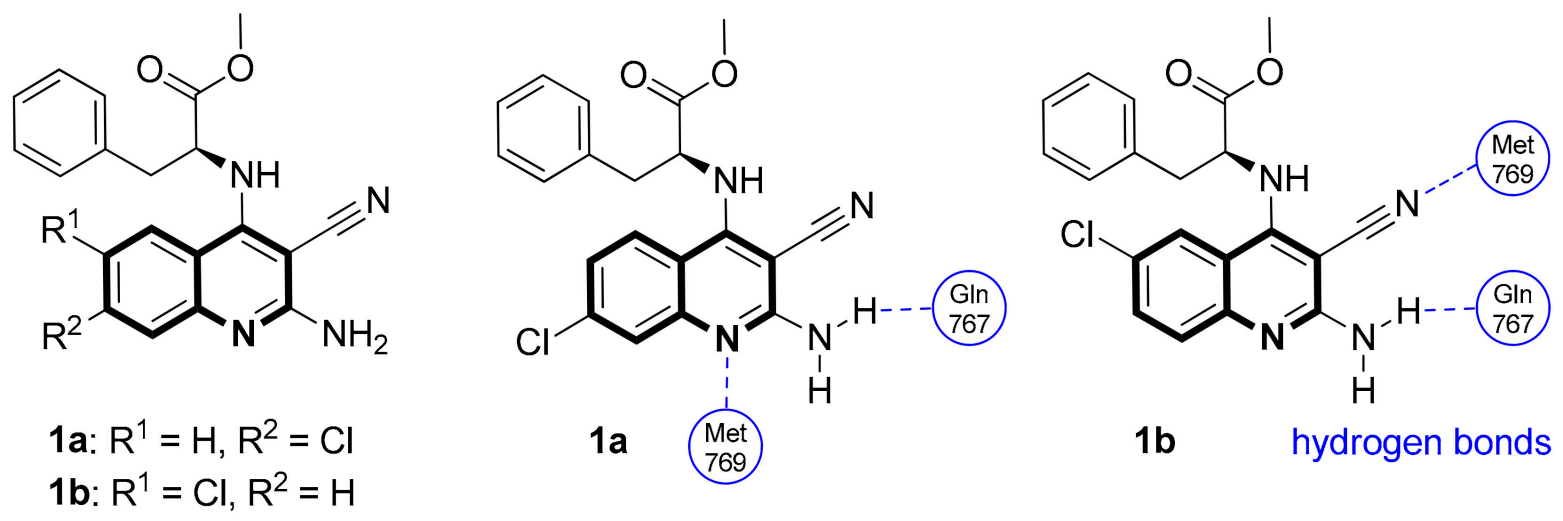
Quinoline derivatives **1a** and **1b**, and their interaction in the EGFR active site.

**Figure 8:**
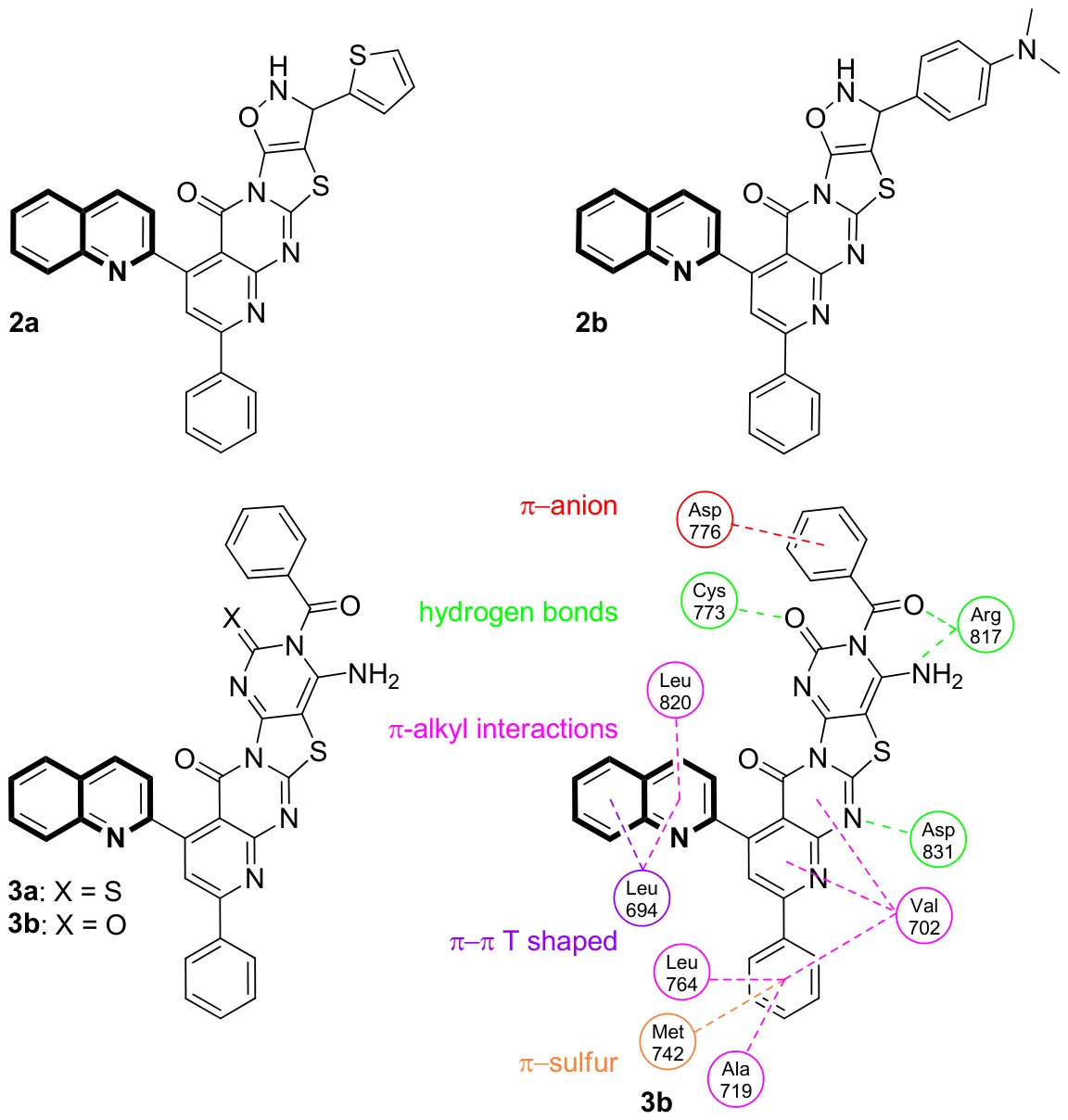
Quinoline derivatives **2a**, **2b**, **3a**, and **3b** with anticancer properties, and molecular interaction of compound **3b** with EGFR.

**Figure 9:**
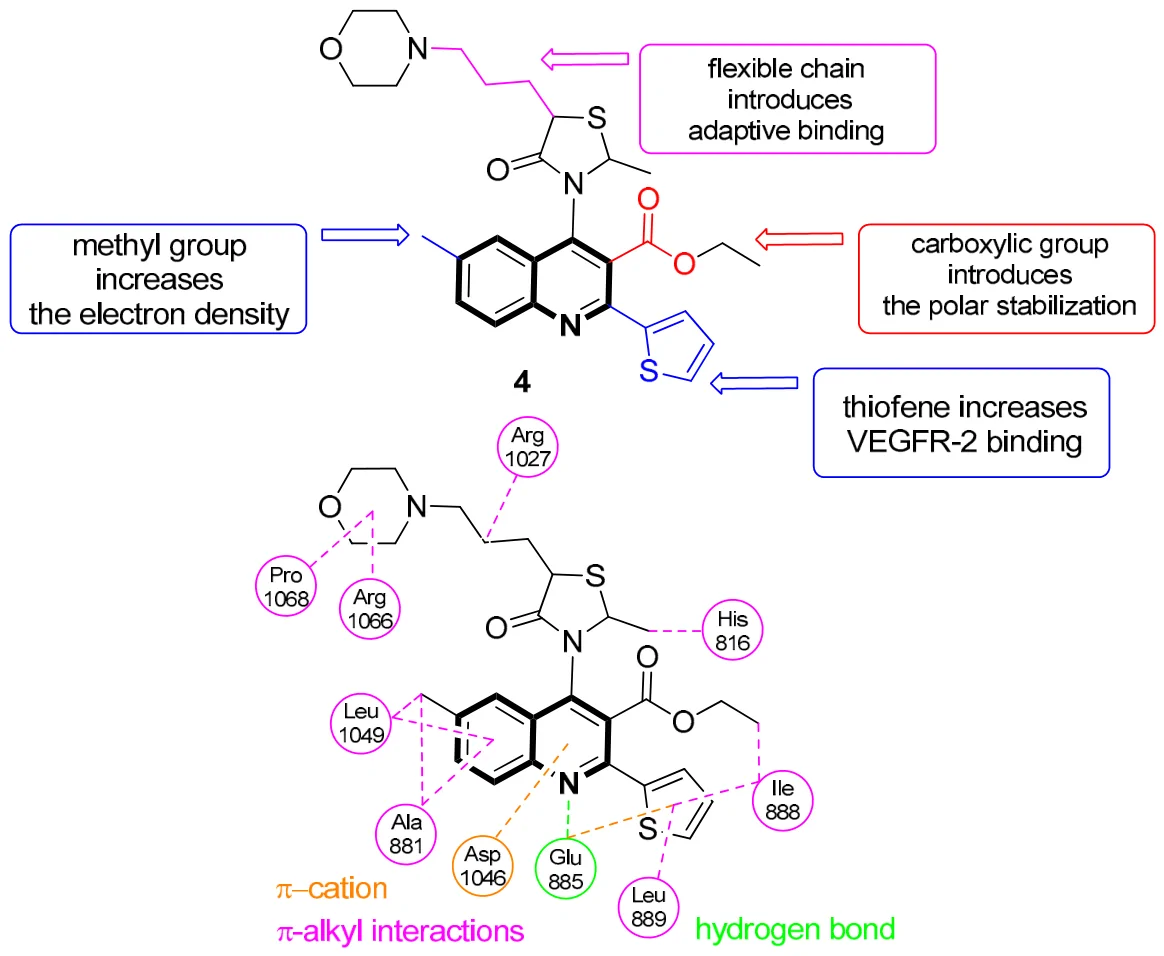
Quinoline **4** and its interaction with VEGFR-2 ATP-binding cavity.

**Figure 10:**
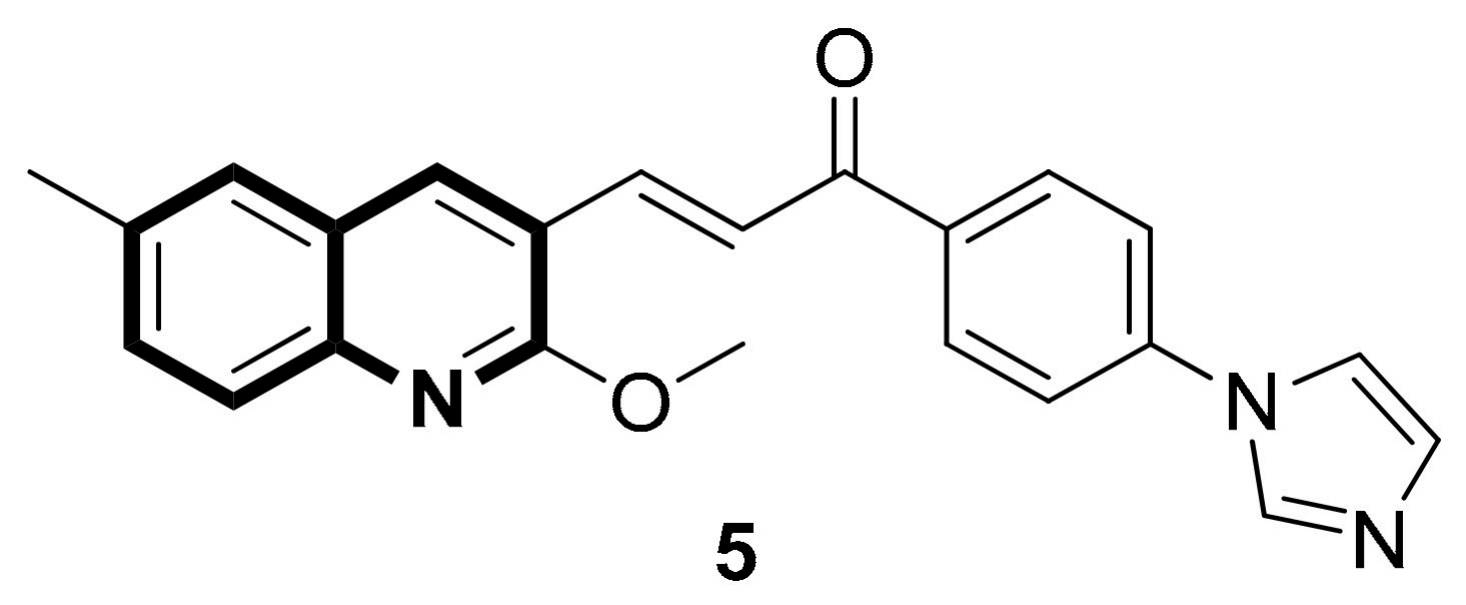
Quinoline–chalcone hybrid **5**.

**Figure 11:**
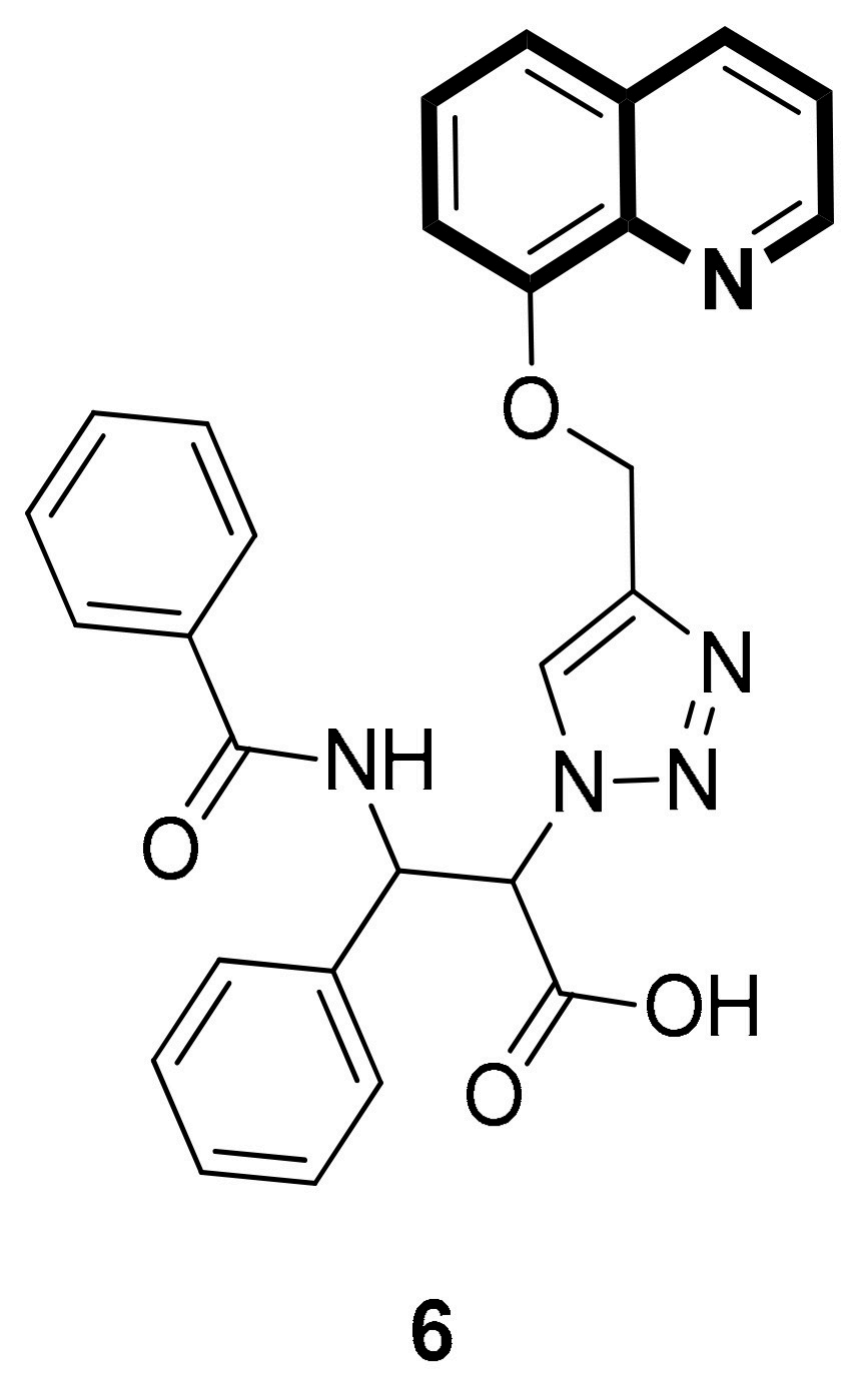
Antiangiogenic isoserine derivative composed of 1,2,3-triazole and quinoline **6**.

**Figure 12:**
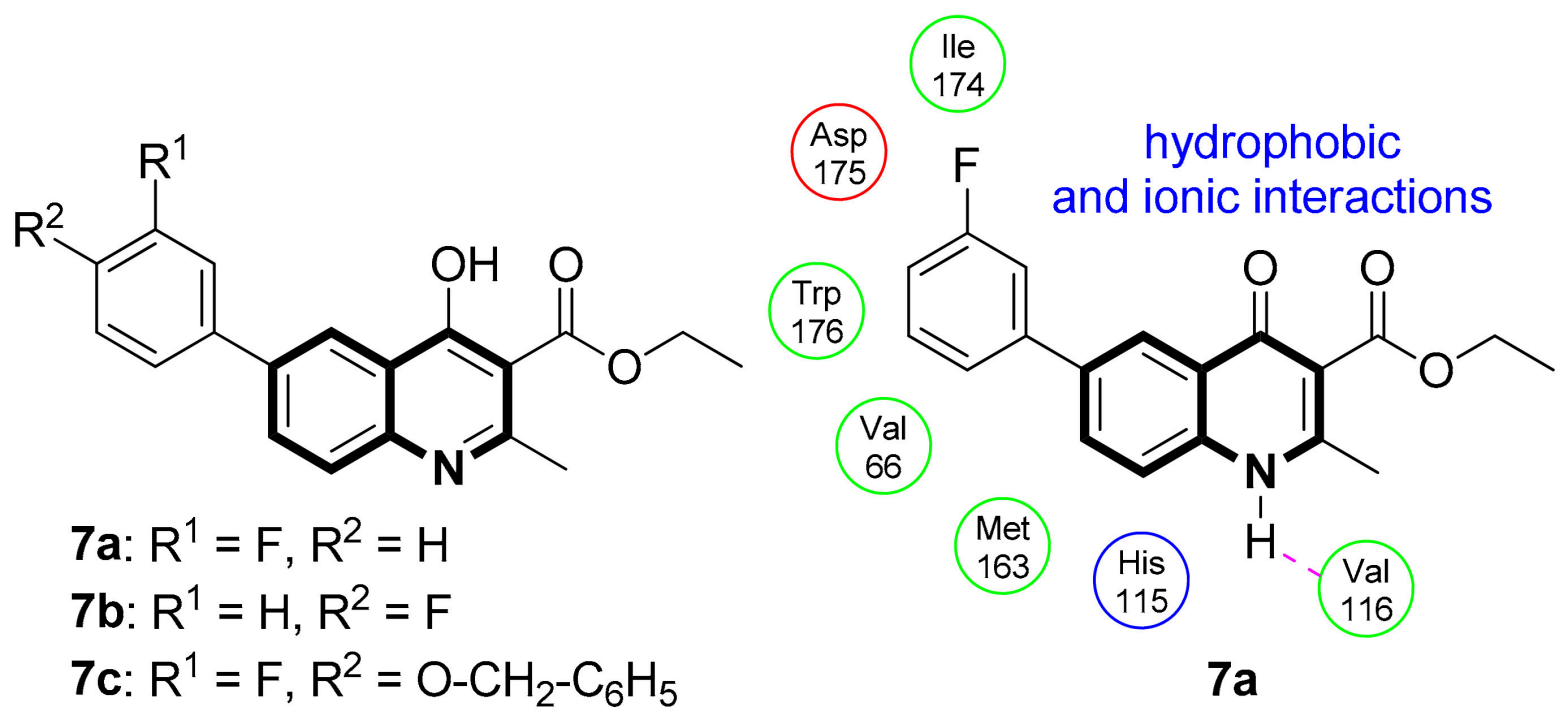
6-Arylquinoline derivatives **7a**, **7b**, and **7c**. Interaction of ethyl 6-(3-fluorophenyl)-4-hydroxy-2-methylquinoline-3-carboxylate (**7a**) with Casein kinase 2 (CK2).

**Figure 13:**
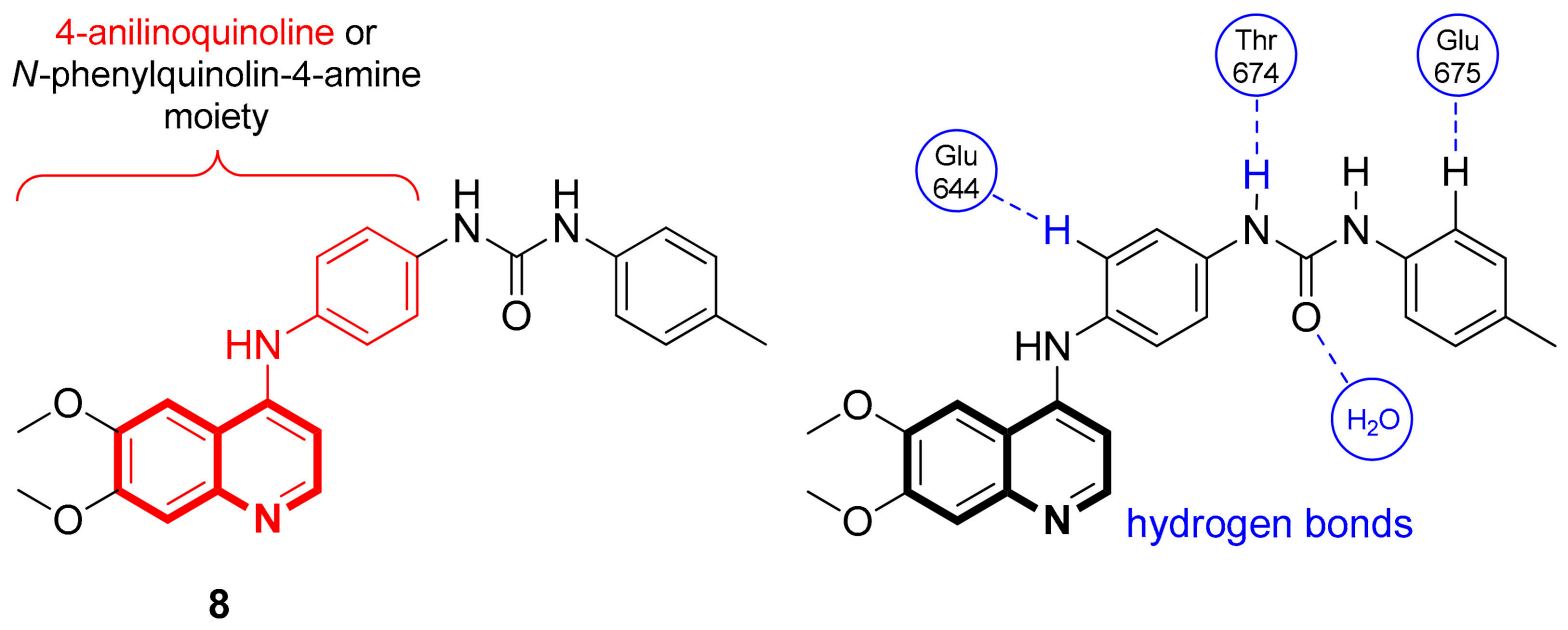
Quinoline-based hybrid **8** and its 2D-molecular docking results to PDGFR-α.

**Figure 14:**
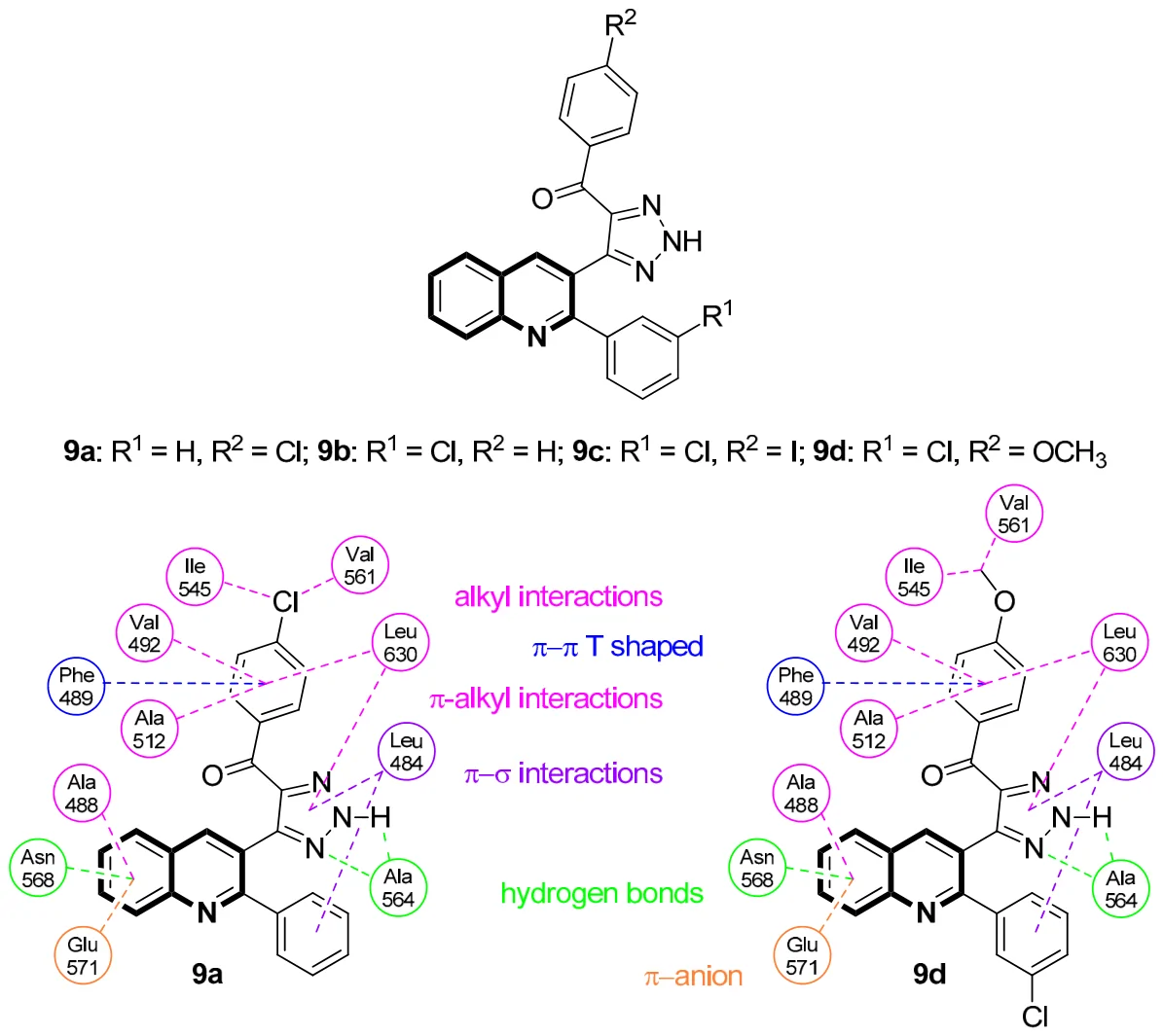
Quinoline-based 1,2,3-triazoles **9a**, **9b**, **9c**, and **9d** designed by Bhukya and coworkers, and two-dimensional (2D) binding interactions of compounds **9a** and **9d** against fibroblast growth factor receptor 1 (FGFR1).

**Figure 15:**
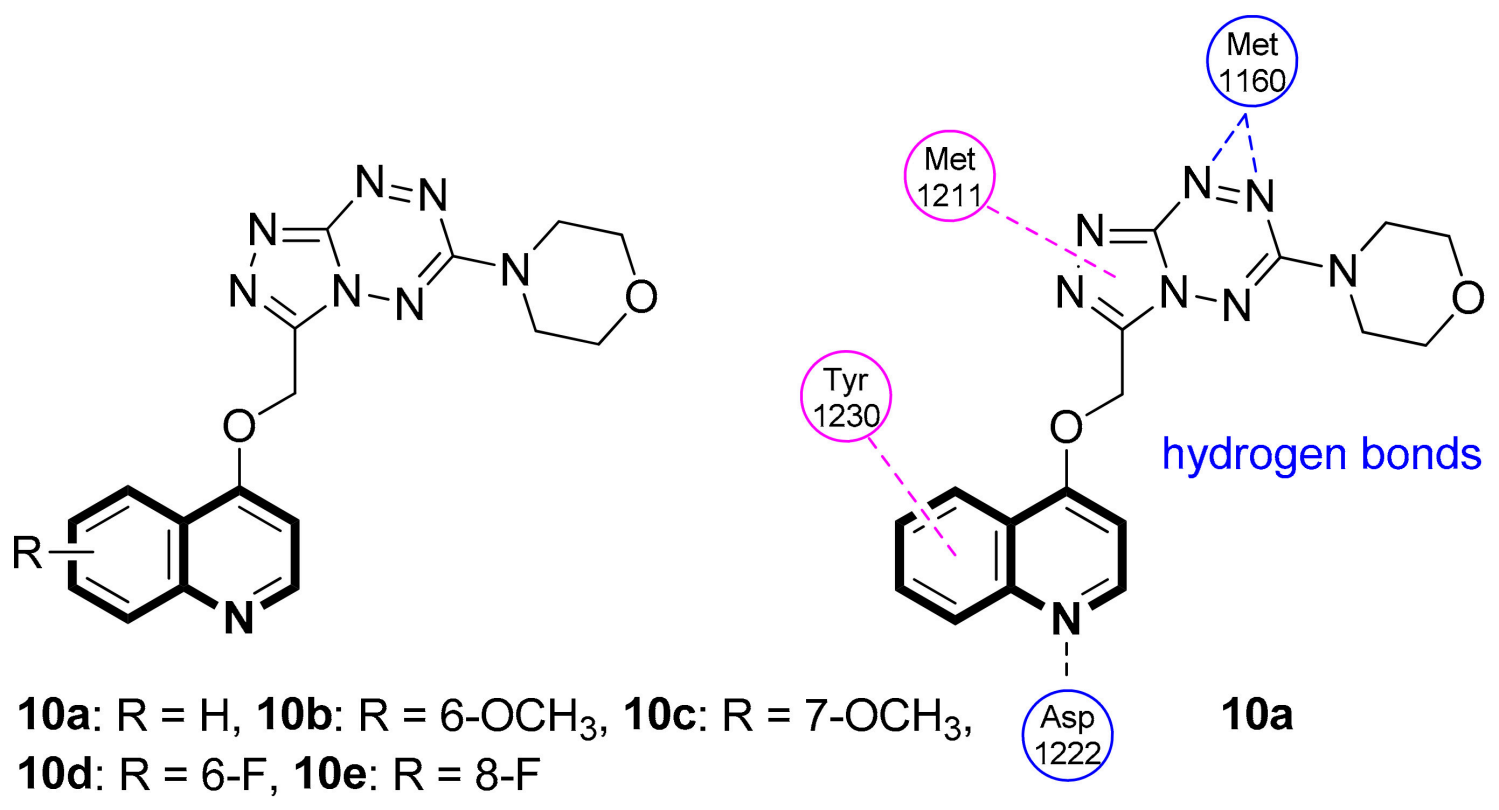
Triazolo–tetrazine derivatives containing quinoline **10a**–**e** and compound **10a** docked into c-Met (entry 4DEH in the Protein Data Bank).

**Figure 16:**
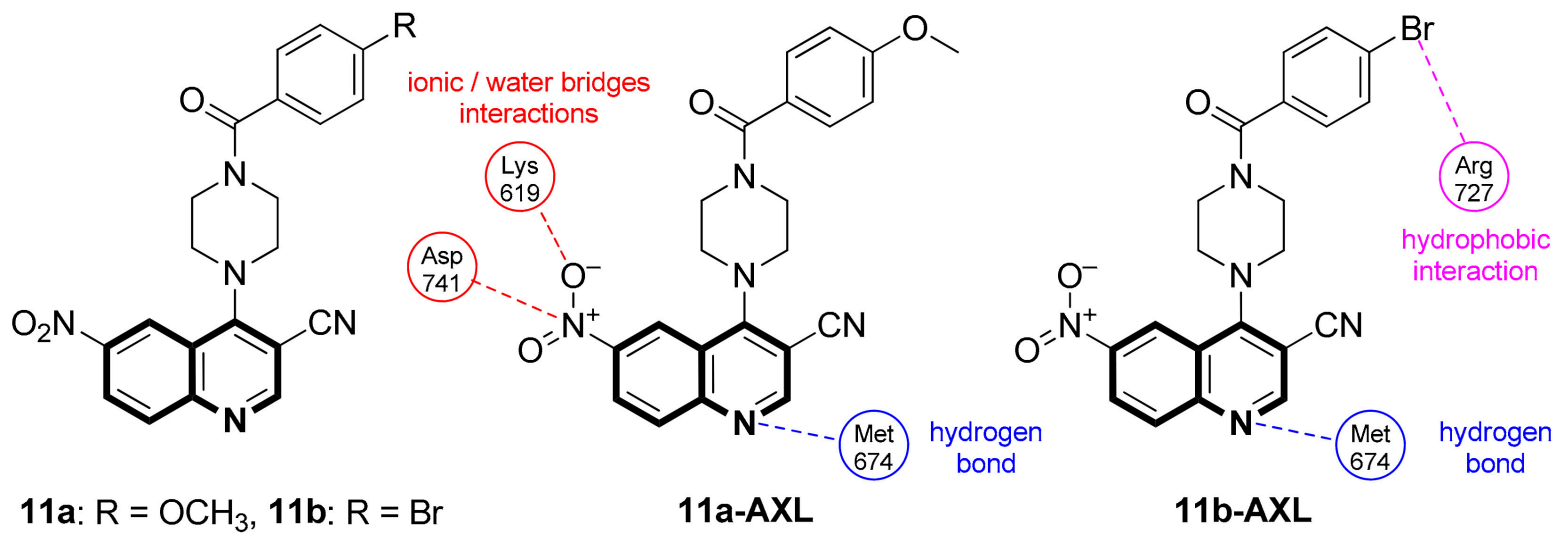
Designed by La Monica et al. [100] 4-(4-benzoylpiperazin-1-yl)-6-nitroquinoline-3-carbonitrile quinoline-hydrazinylpyrimidine derivatives **11a** and **11b** as anticancer agents, and ligand–protein interaction analysis for the complexes of **11a** and **11b** with oncogenic protein AXL.

**Figure 17:**
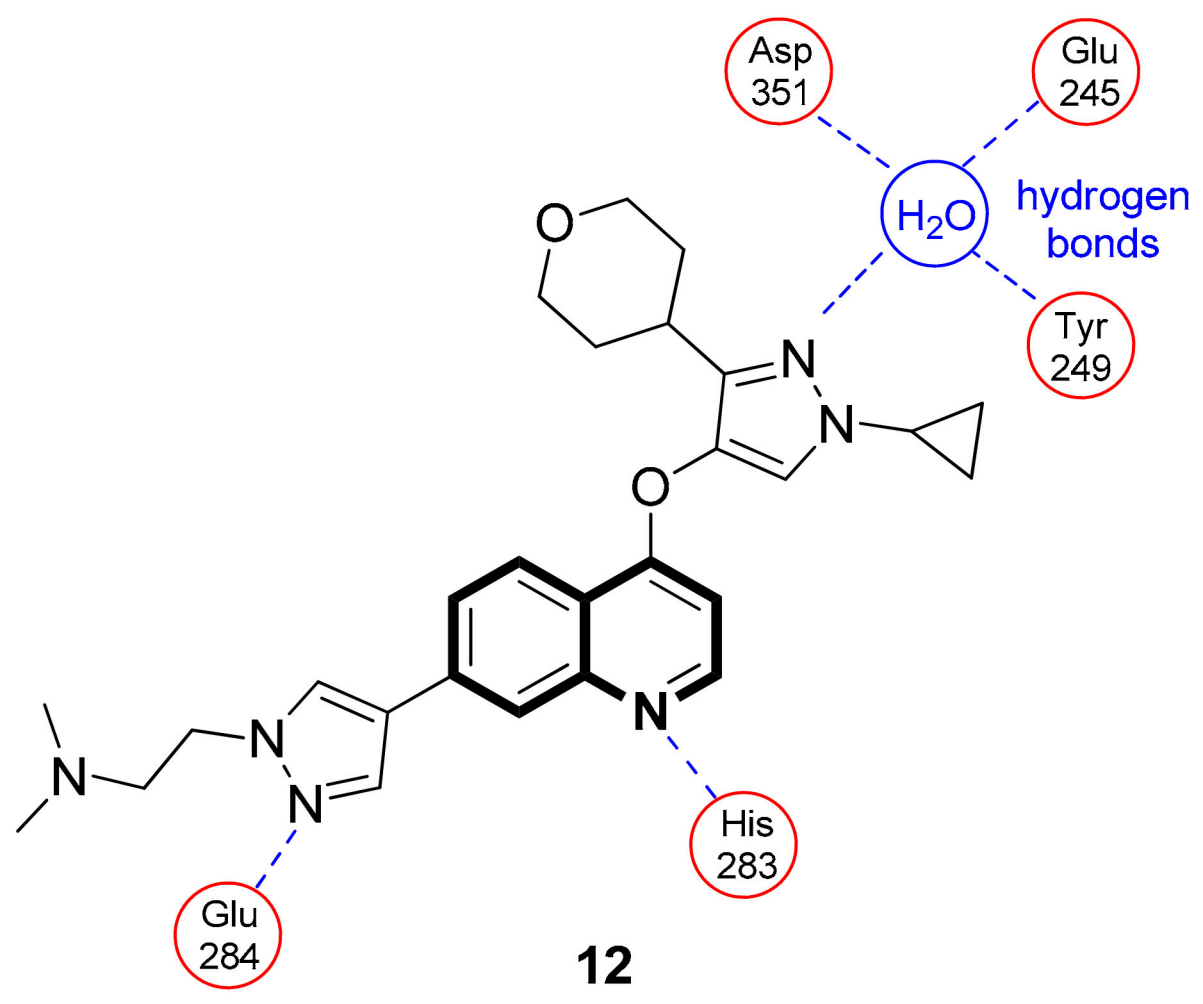
Designed by Liu et al. [102] quinoline-based TGFβR1 inhibitor **12**.

**Figure 18:**
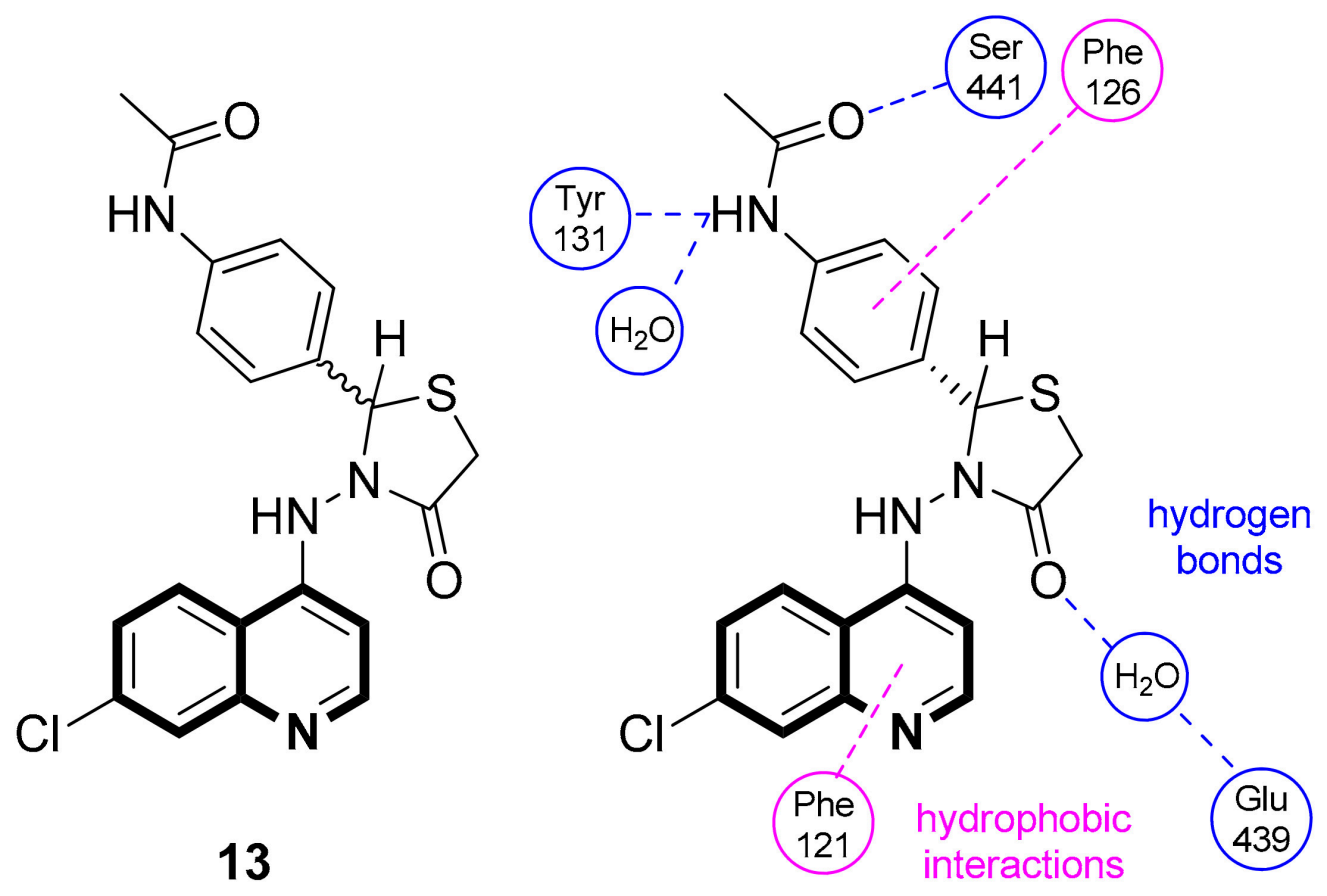
Quinoline derivative **13** and essential interactions of TLR4-**13** complex.

**Figure 19:**
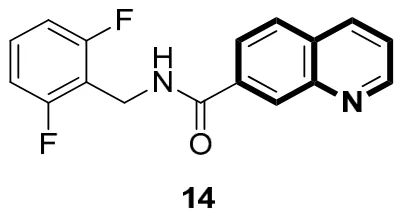
Designed by Ishaq et al. quinoline-6-carboxamide derivative **14** with ectonucleotidase-inhibitory and antiproliferative activity.

**Figure 20:**
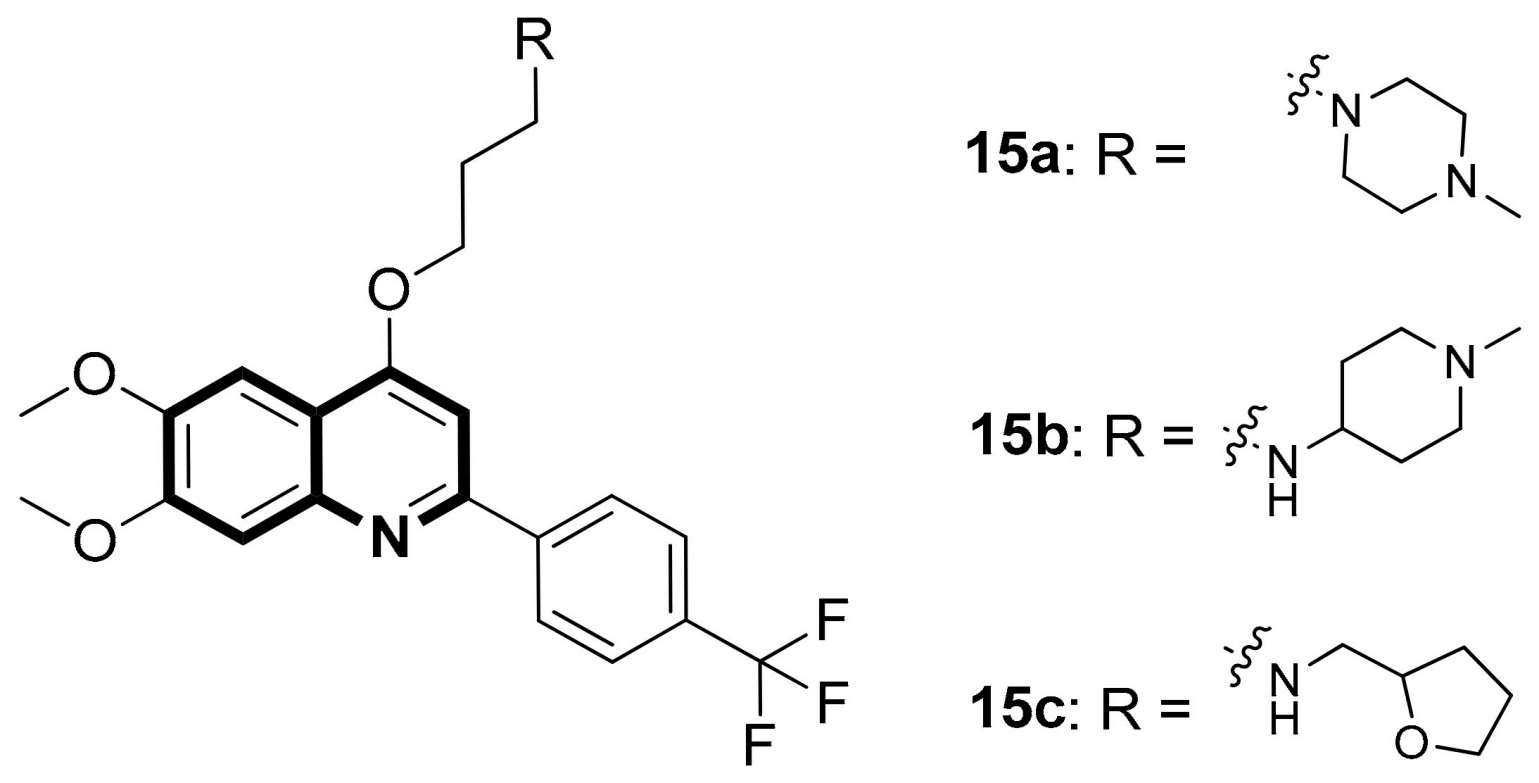
Arylquinoline-based EGFR and FAK kinase inhibitors **15a**, **15b**, and **15c**.

**Figure 21:**
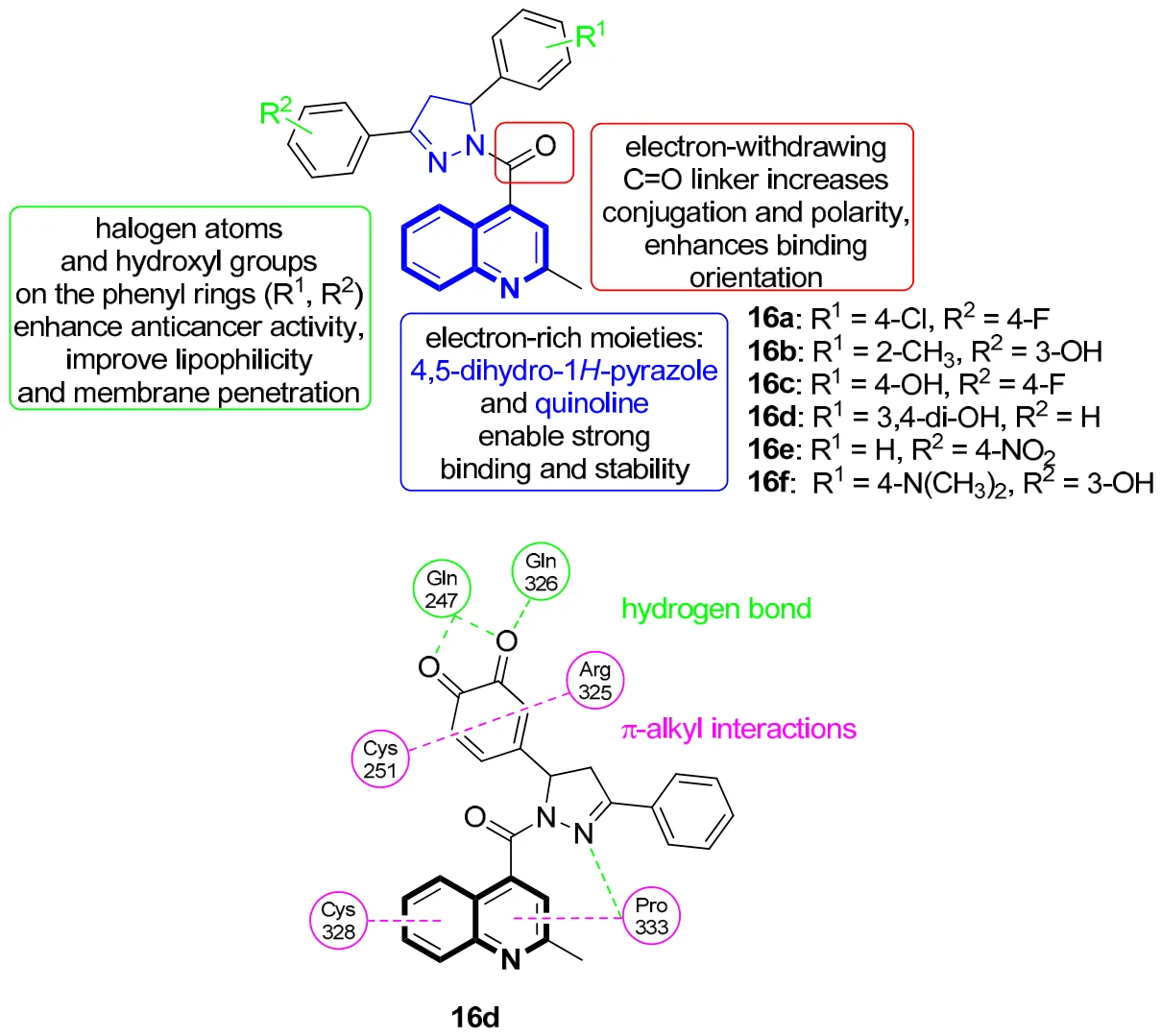
Putative dual EGFR–STAT3 inhibitors **16a**, **16b**, **16c**, **16d**, **16e**, and **16f**. Two-dimensional interaction of compound **16d** with the STAT3 protein.

**Figure 22:**
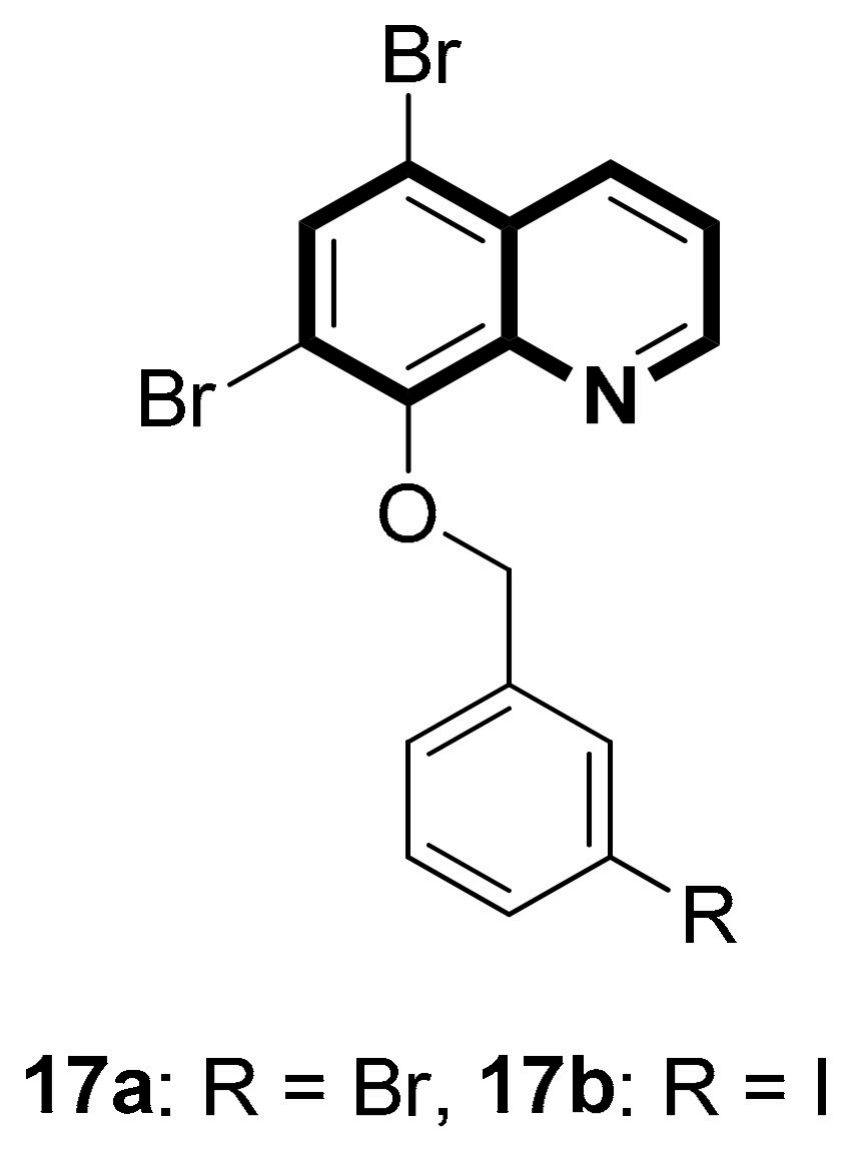
Novel 8-(benzyloxy)-5,7-dibromo-2-methylquinolines **17a** and **17b** as potent anticancer agents against triple-negative breast cancer cells (TNBC).

**Figure 23:**
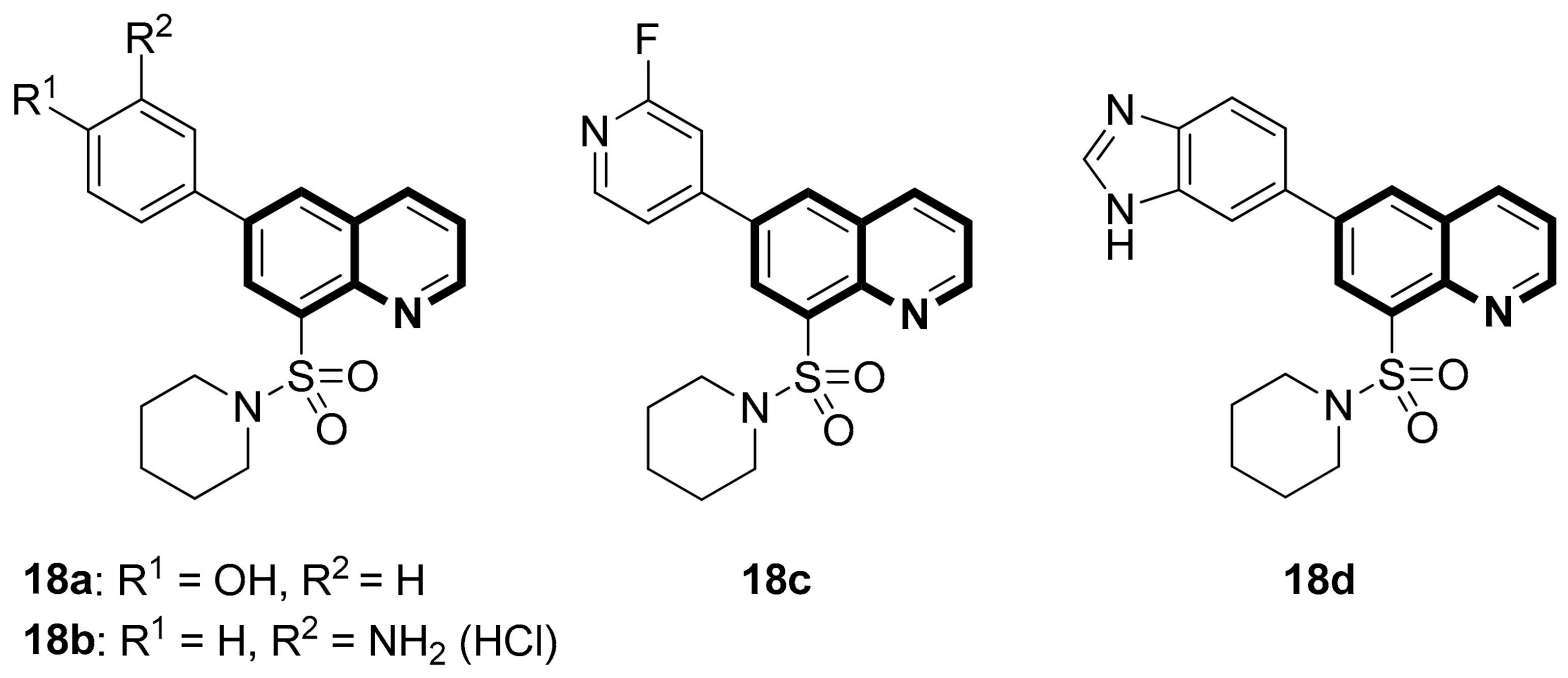
Quinoline–sulfonamide hybrids **18a**, **18b**, **18c**, and **18d** as potential ITK and BTK inhibitors.

**Figure 24:**
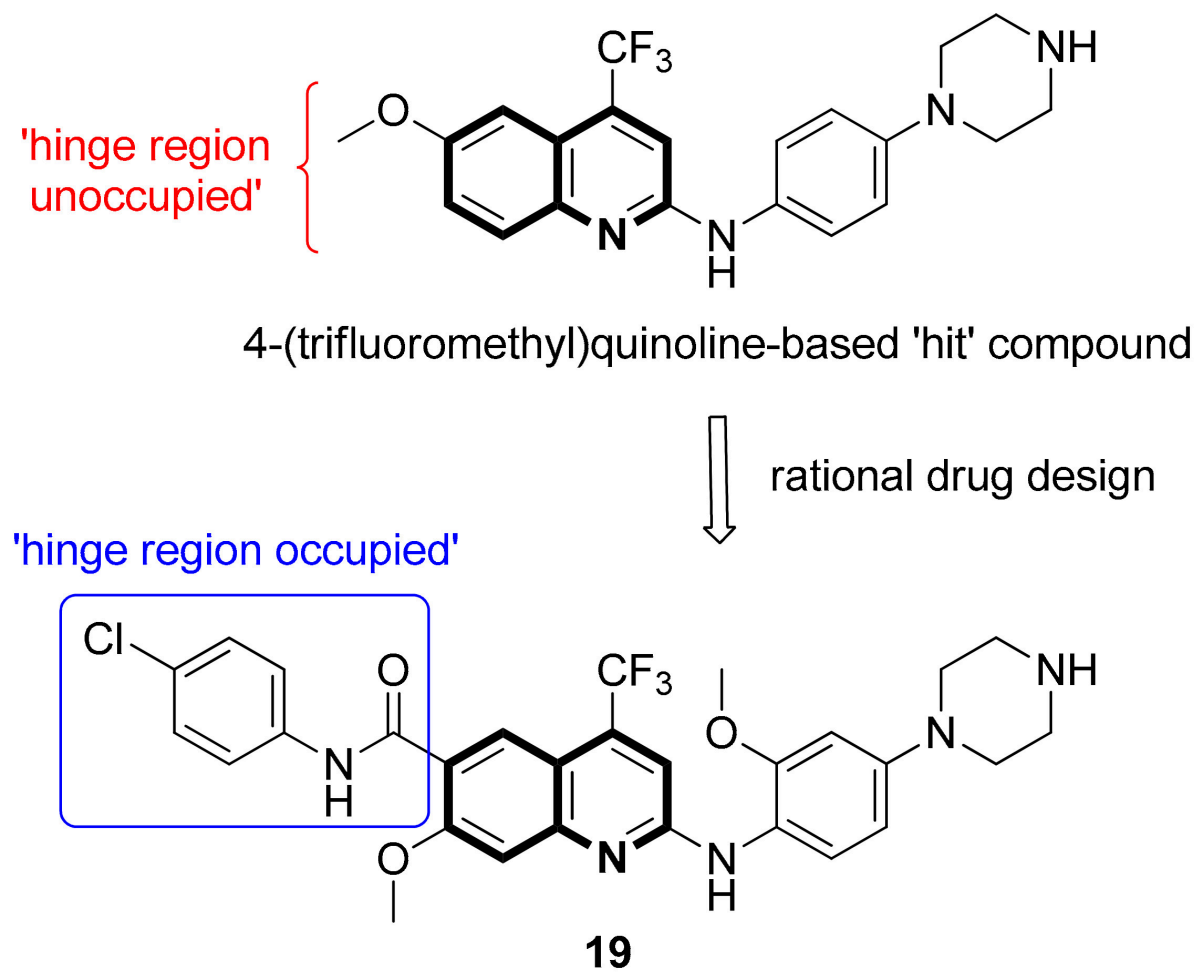
Designed *N*-(4-chlorophenyl)-7-methoxy-2-((2-methoxy-4-(piperazin-1-yl)phenyl)amino)-4-(trifluoromethyl)quinoline-6-carboxamide **19** with anti-prostate cancer activity.

**Figure 25:**
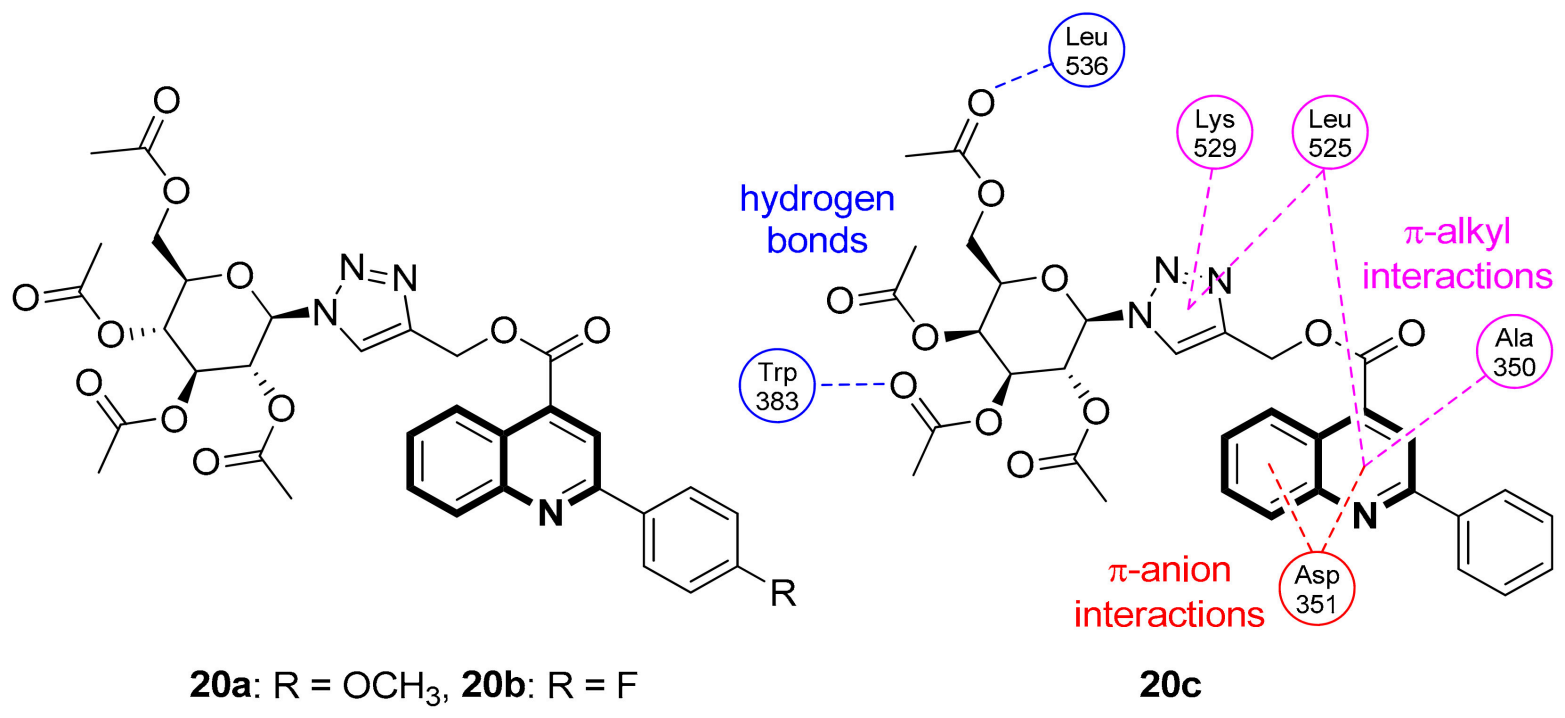
Phenyl-substituted quinoline-1,2,3-triazole glucosyl-conjugates **20a**, **20b**, and 2D bindings mode of galactosyl-conjugate **20c** within the active site of ERα.

**Figure 26:**
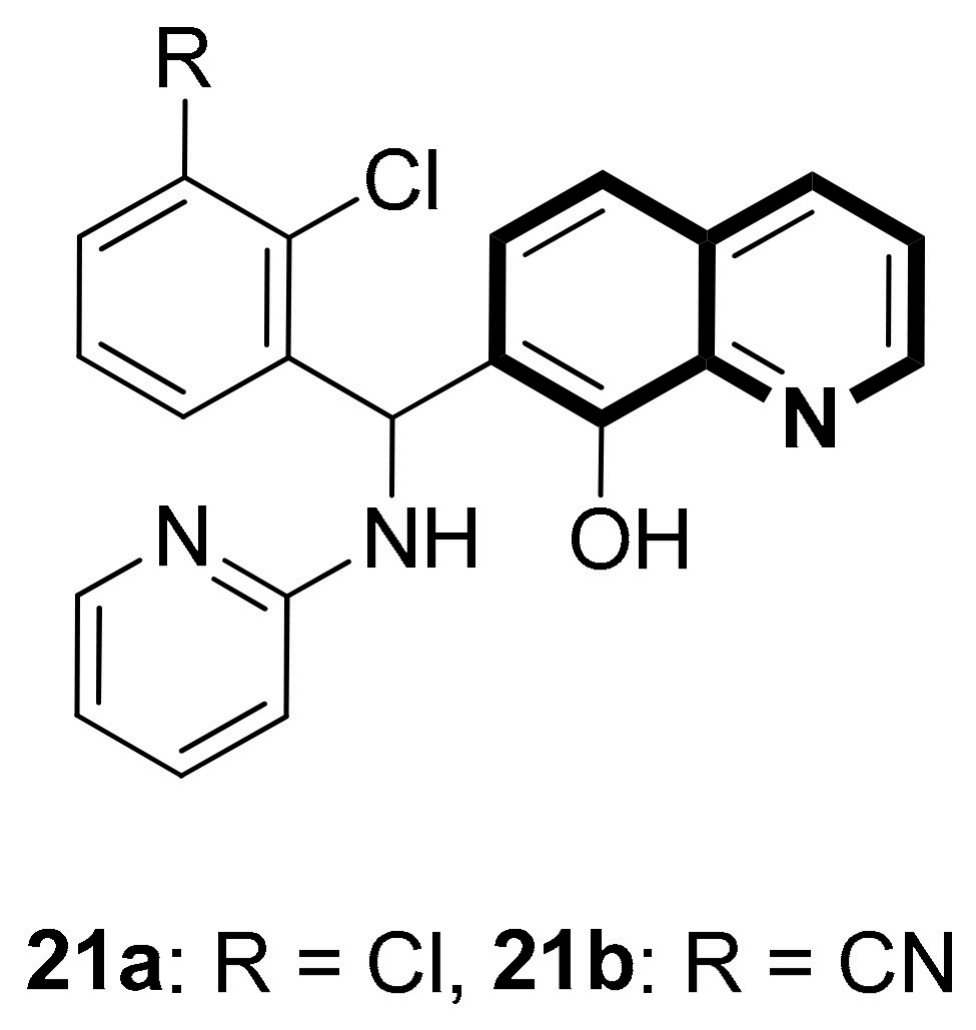
Quinolinol derivatives **21a** and **21b** which promote the proteasomal degradation of the oncogenic MDM2-MDM4 complex.

**Figure 27:**
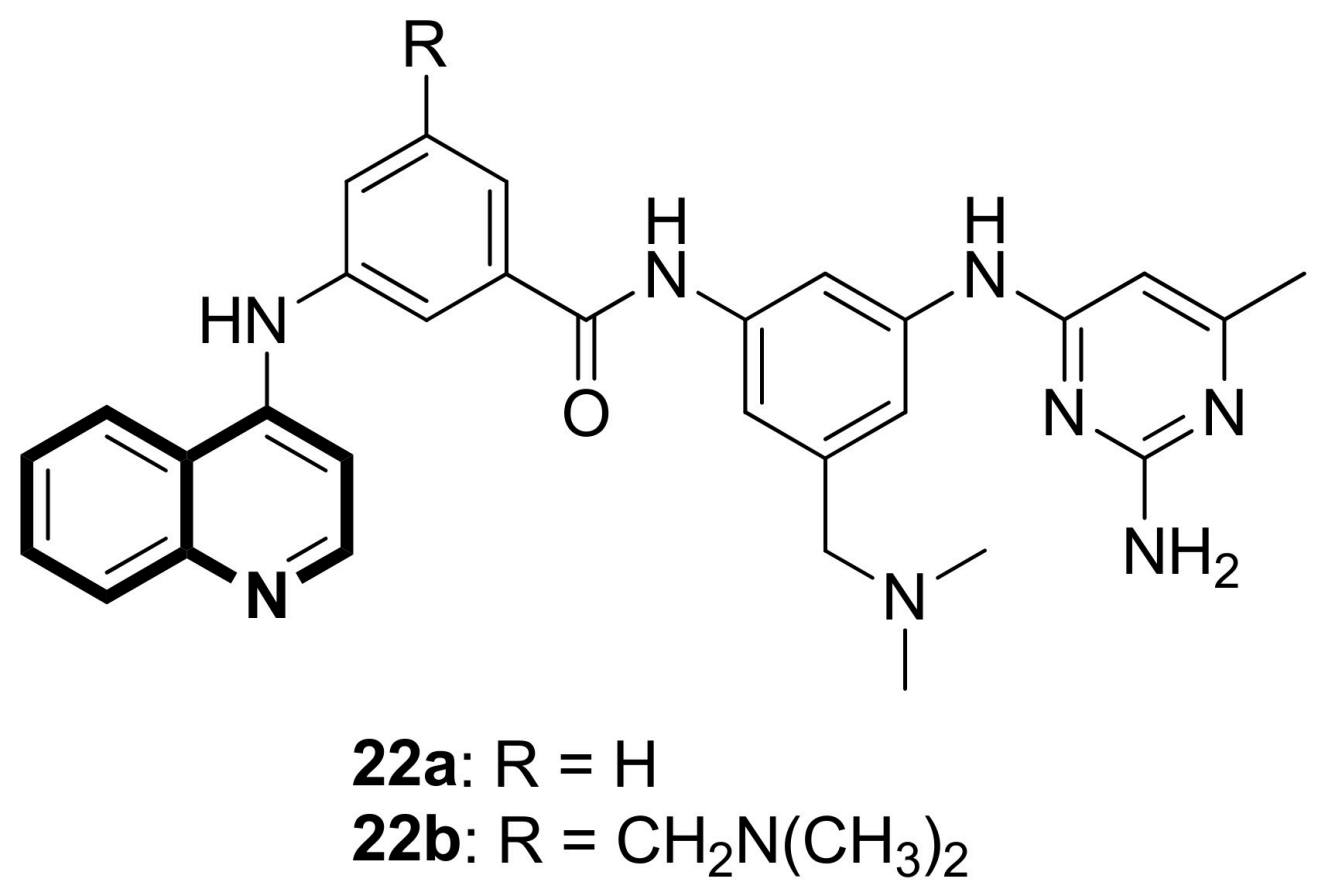
Quinoline derivatives bearing basic chains **22a** and **22b** as DNMT inhibitors.

**Figure 28:**
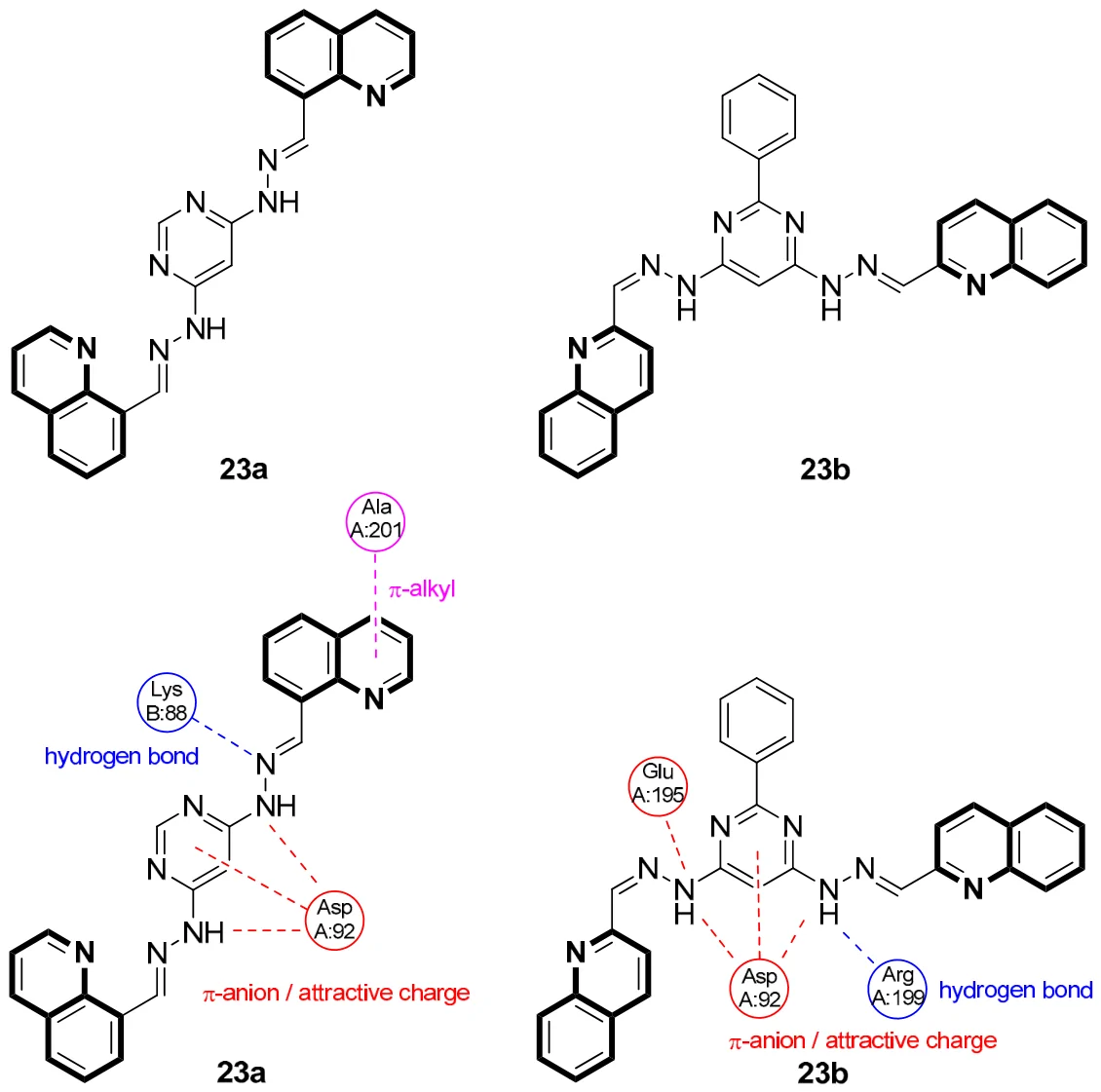
Designed quinoline-hydrazinylpyrimidine derivatives **23a**, **23b** as promising anticancer agents, and molecular interactions—hydrogen bonding, π–π interactions and electrostatic attraction between hydrazones **23a**, **23b** and CDK2.

**Figure 29:**
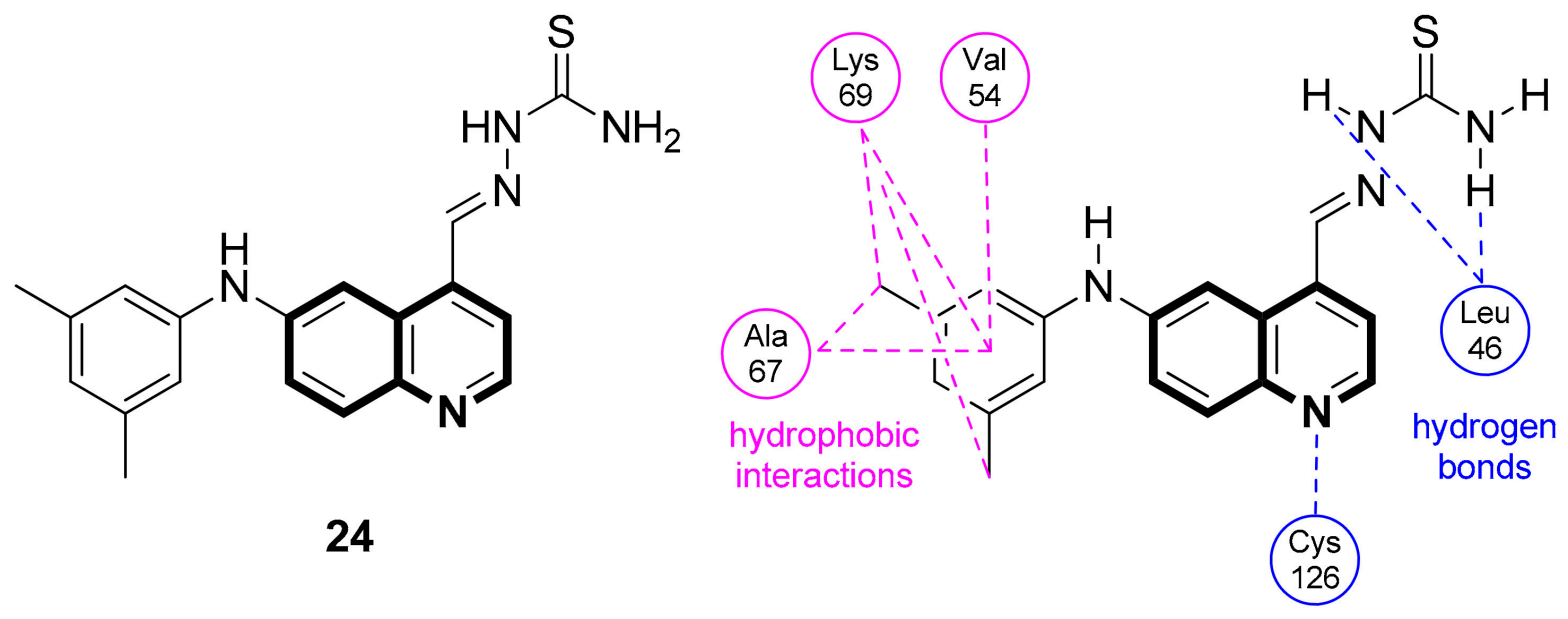
Identified 2-((6-((3,5-dimethylphenyl)amino)quinolin-4-yl)methylene)hydrazinecarbothioamide (**24**) with anticancer activity and its docking results.

**Figure 30:**
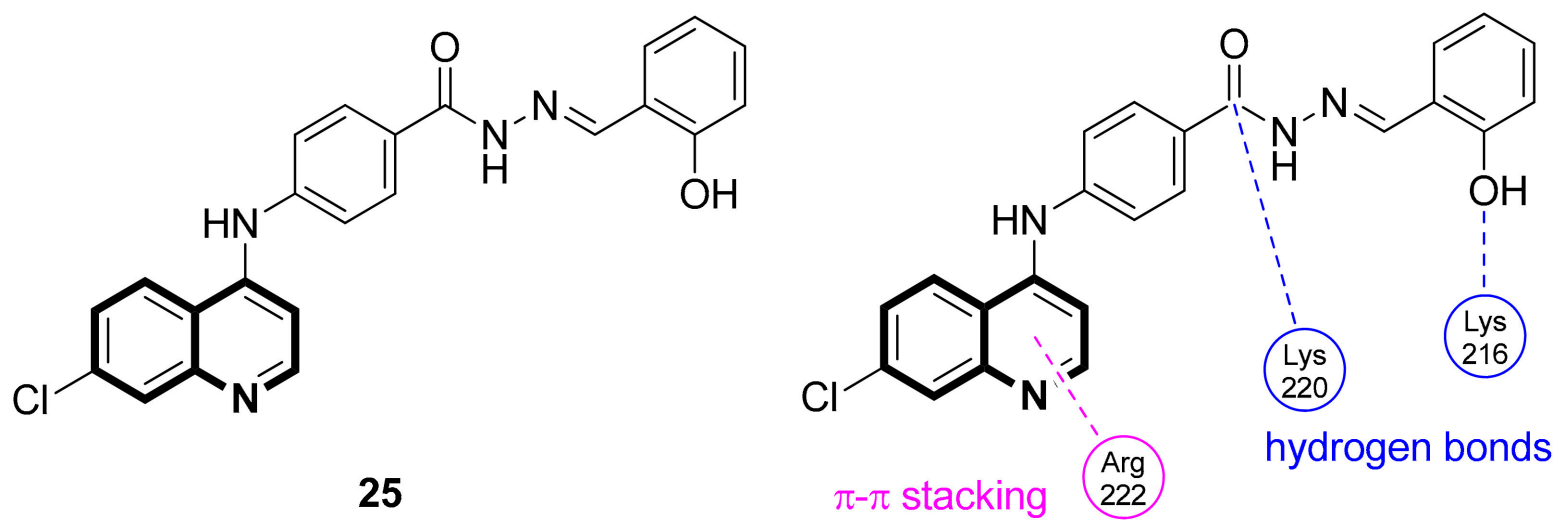
Designed potential KSP (Eg5) protein inhibitor **25**.

**Figure 31:**
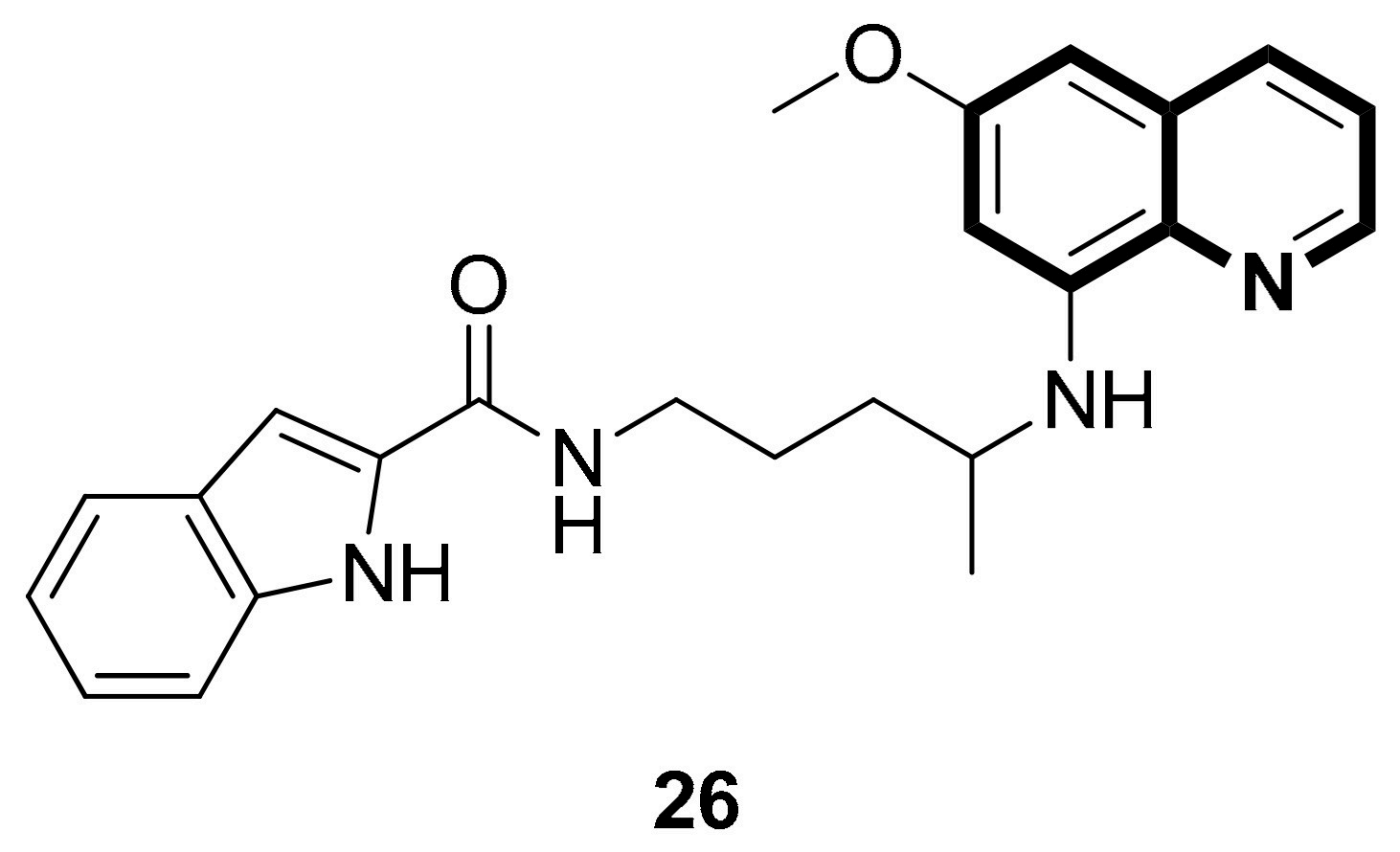
*N*-(4-((6-methoxyquinolin-8-yl)amino)pentyl)-1*H*-indole-2-carboxamide (**26**) with anticancer potency.

**Figure 32:**
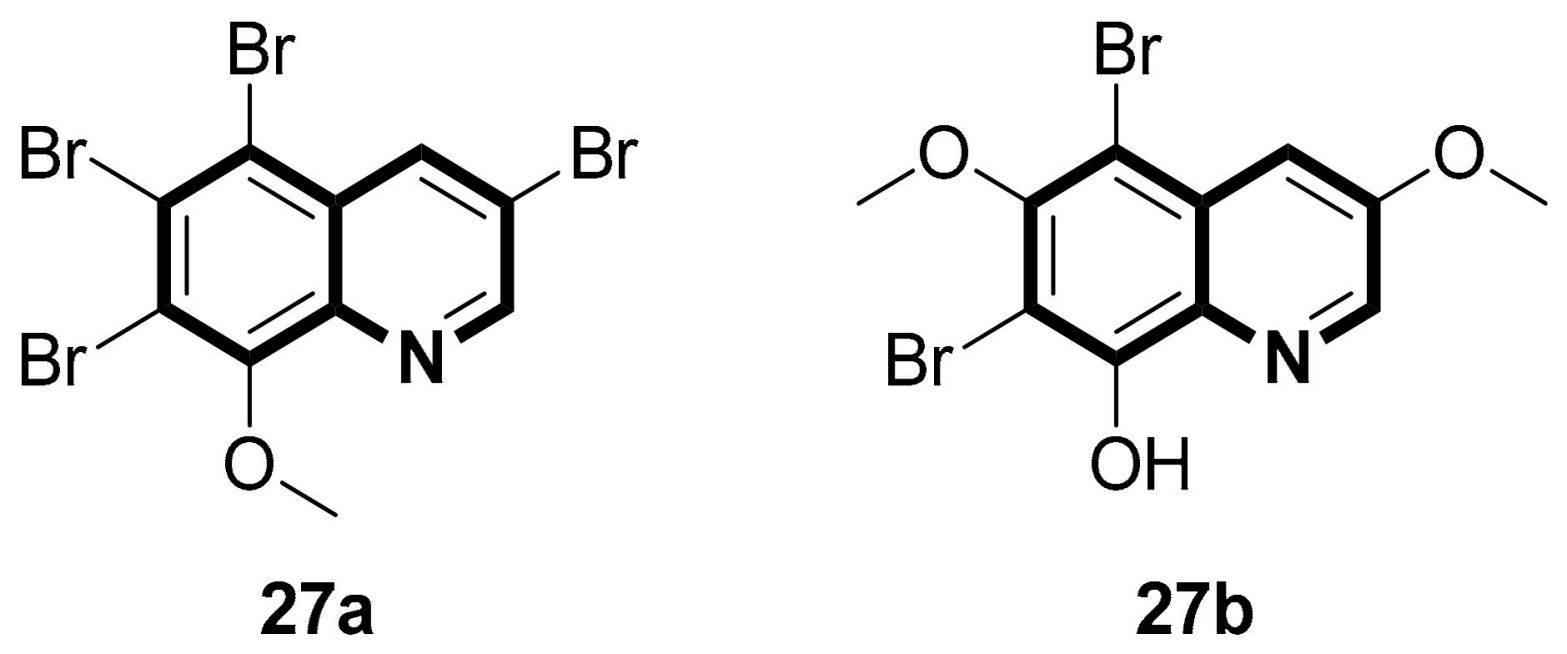
Brominated quinolines **27a** and **27b** with antiproliferative activity.

**Figure 33:**
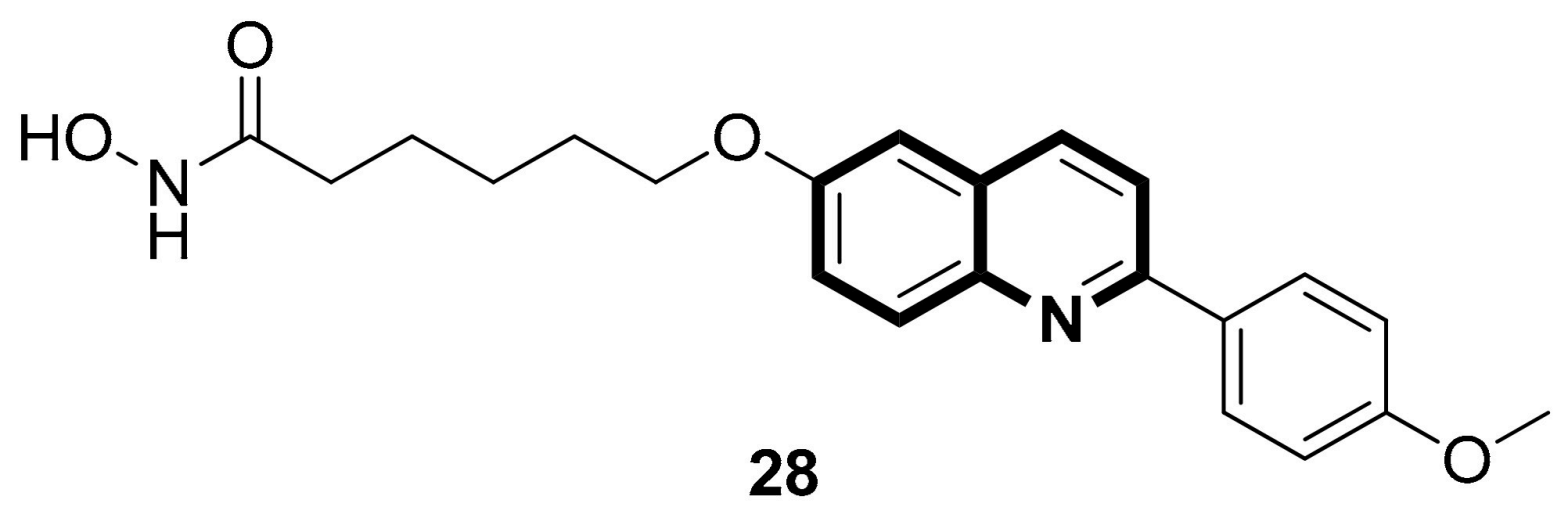
Promising quinoline-based derivative **28** exerting anticancer activity.

**Figure 34:**
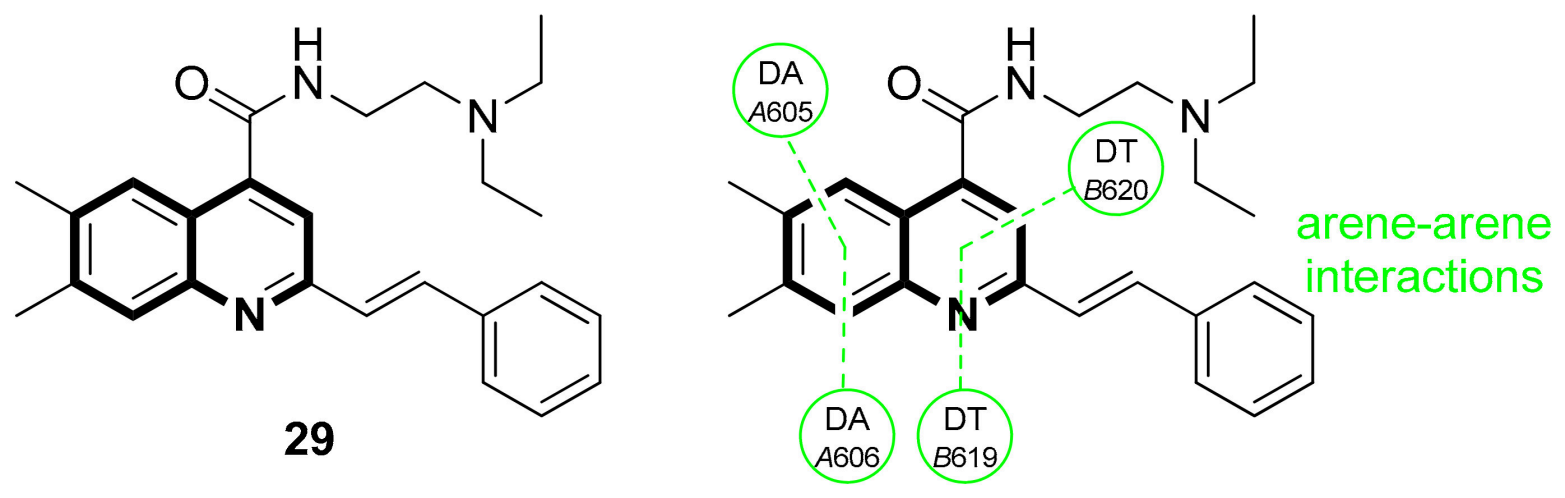
2-Styrylquinoline derivative **29** with anticancer properties and its 2D molecular binding mode within the intercalation site of ctDNA.

**Figure 35:**
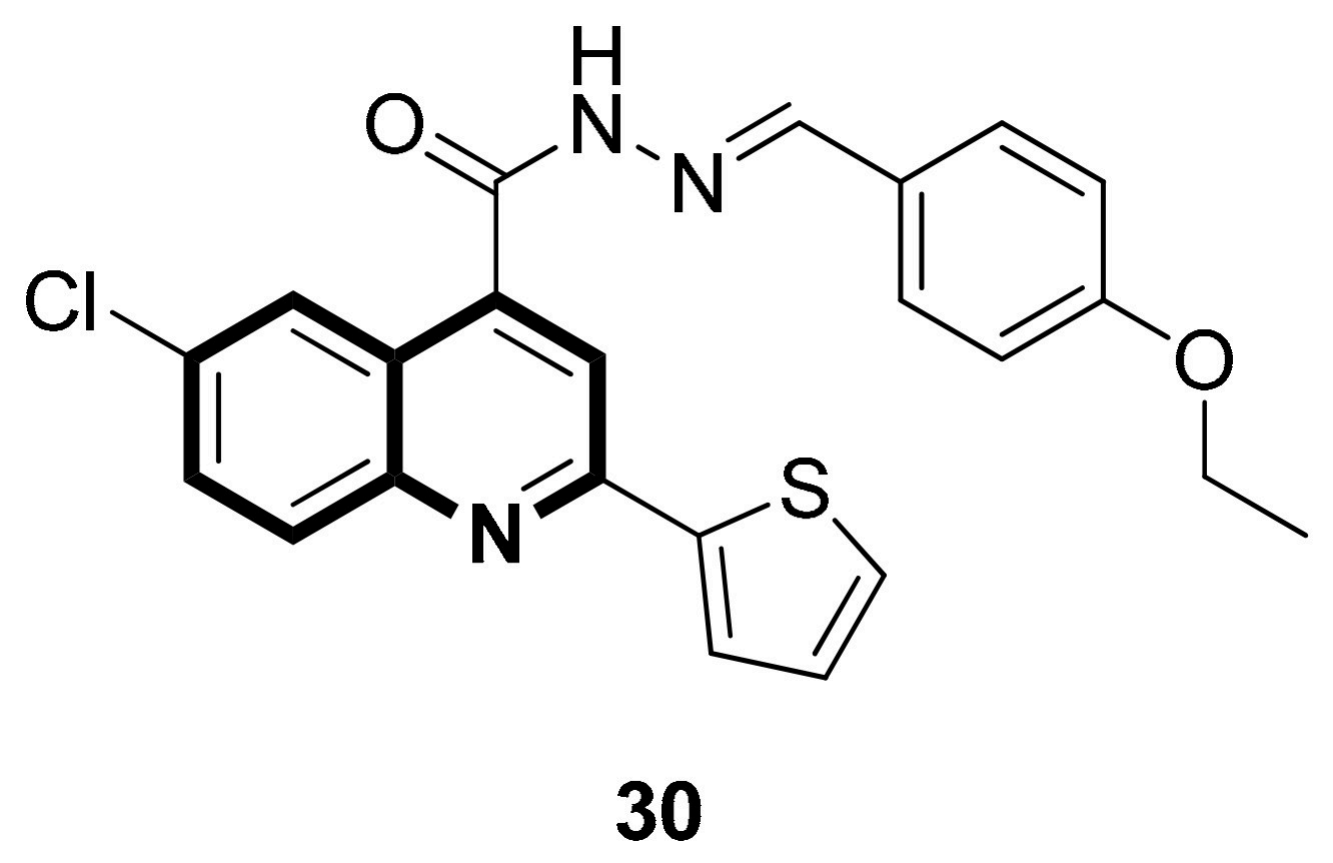
Promising quinoline derivative **30** exerting anticancer activity.

**Figure 36:**
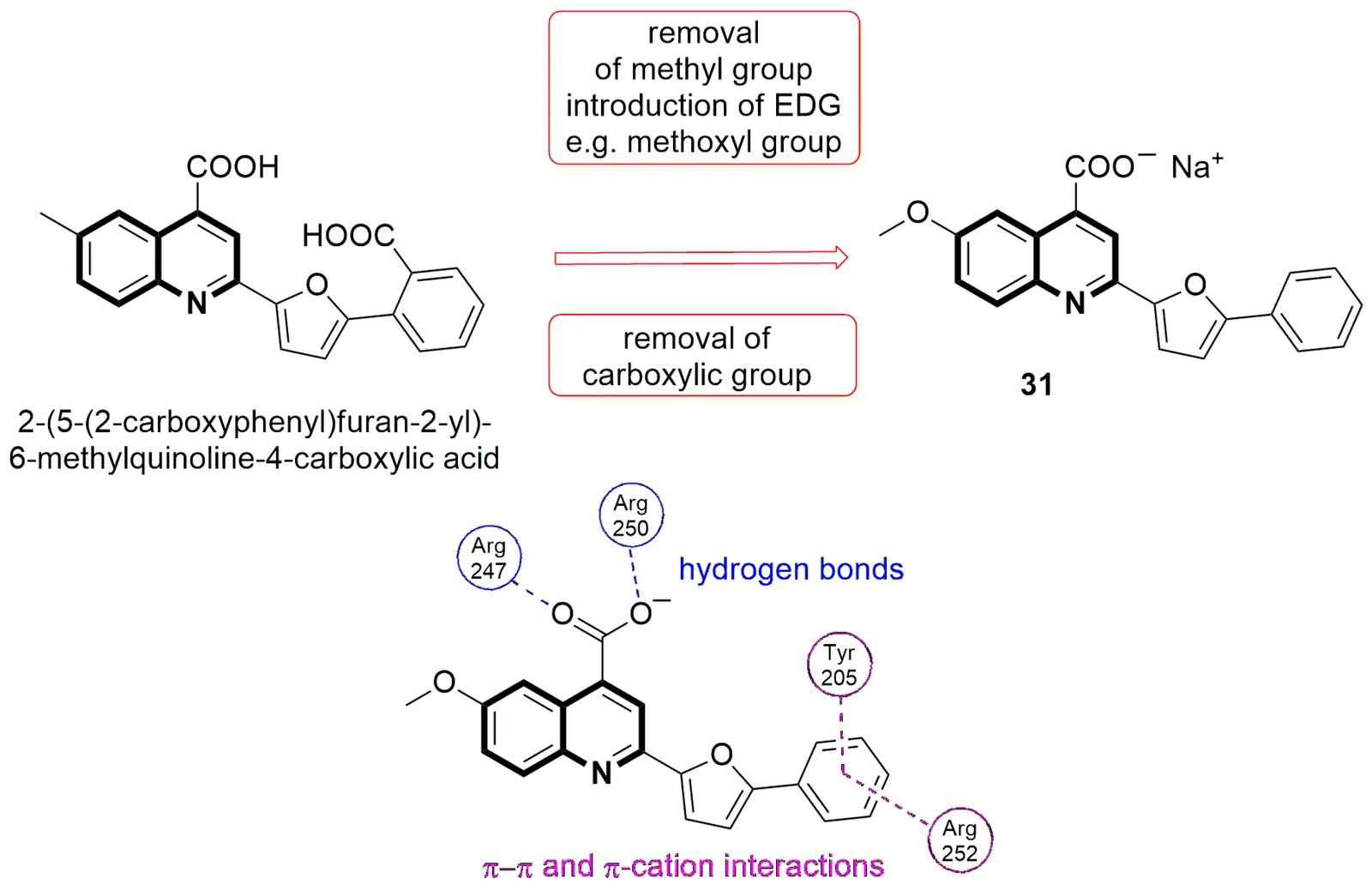
Quinoline derivative **31** which effectively inhibits the RAD51-BRCA2 interaction and molecular docking results for compound **31** into the LFDE binding site of the RAD51 crystal structure.

**Figure 37:**
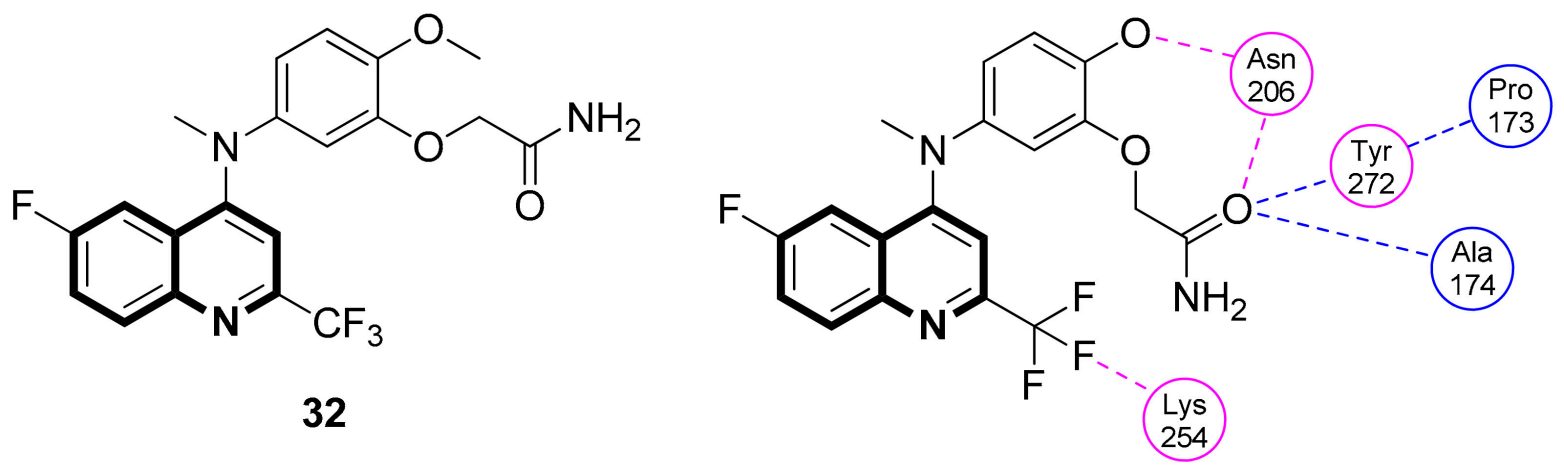
Anticancer 6-fluoro-2-(trifluoromethyl)quinoline derivative **32** and its 2D docking results to the colchicine binding site of tubulin (PDB: 5LYJ).

**Figure 38:**
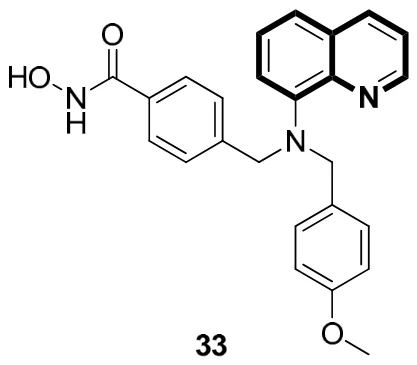
Selective HDAC6 inhibitor **33**.

**Figure 39:**
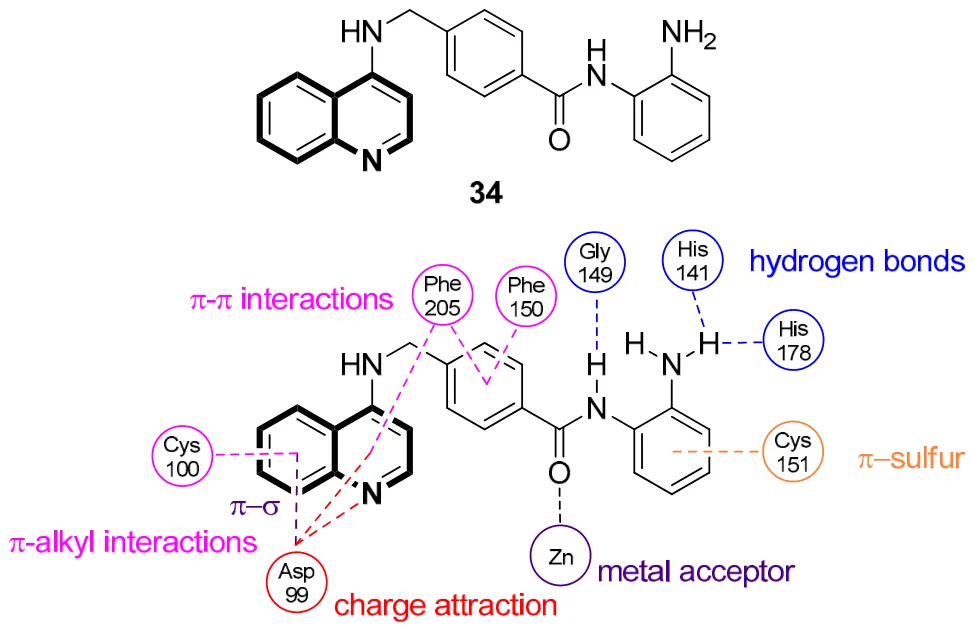
Designed by Yu’s group *N*-arylamide-quinoline-based HDAC inhibitor **34** and its 2D binding mode with HDAC1.

**Figure 40:**
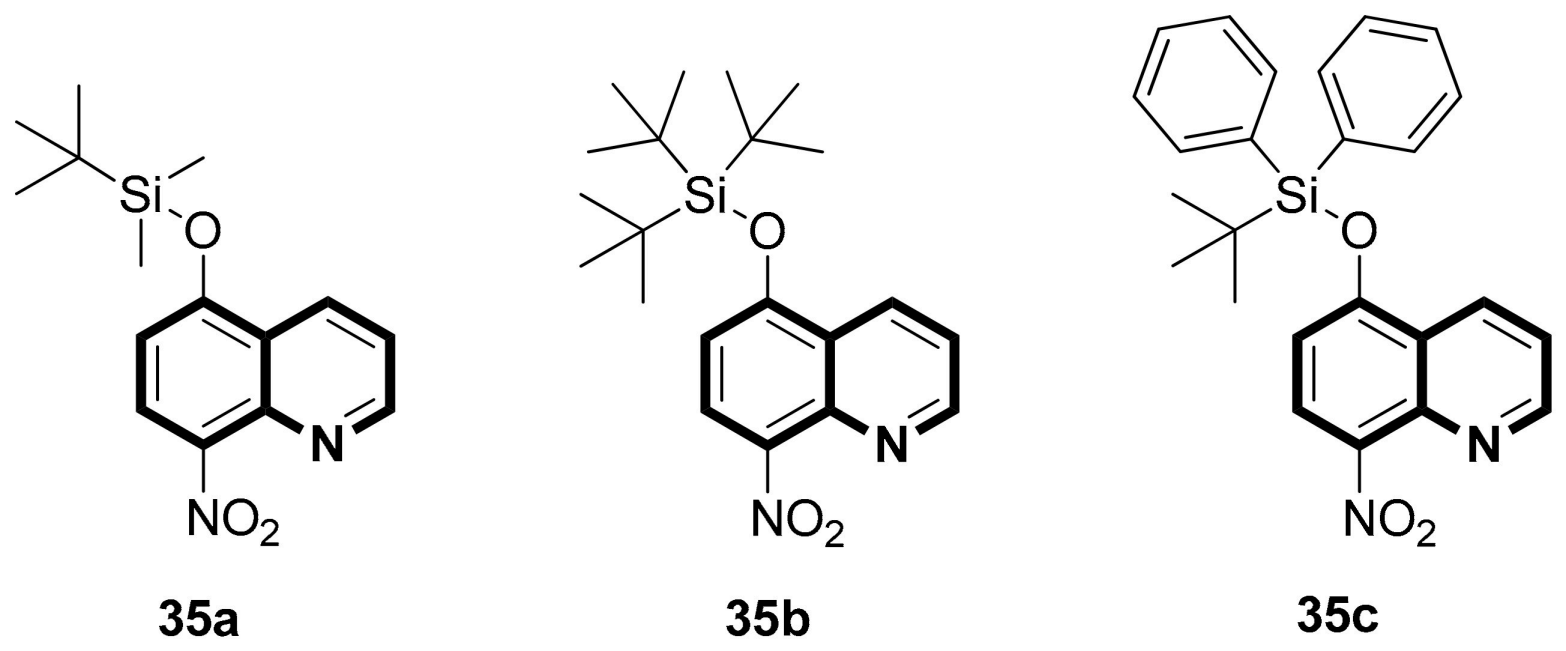
Silyl(oxy)-5-nitroquinolines **35a**, **35b**, and **35c** with MetAP2 inhibitory activity.

**Figure 41:**
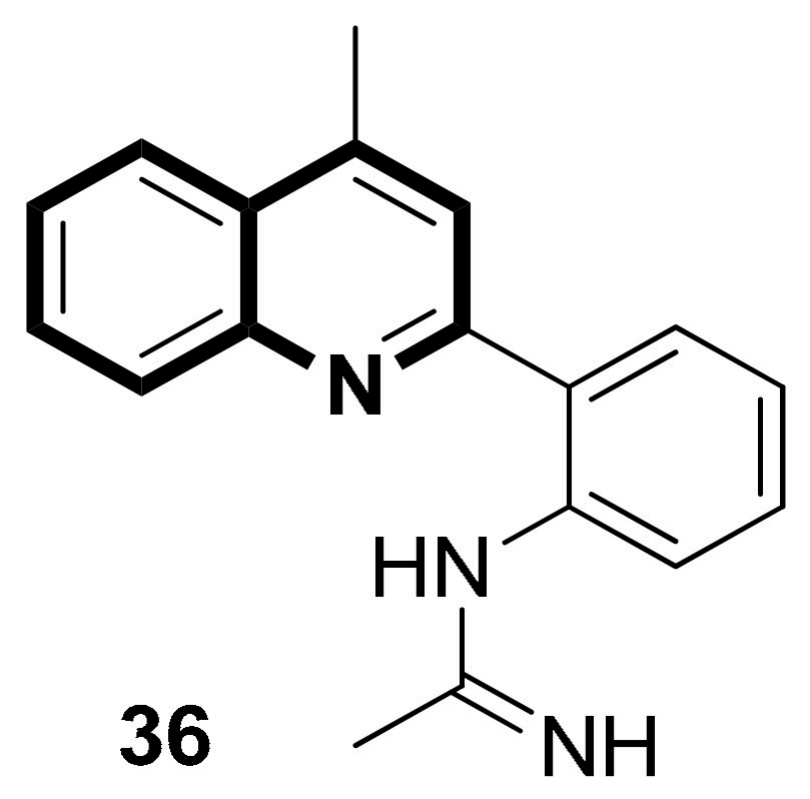
Designed by Gallorini et al. [167] *N*-(2-(4-methylquinolin-2-yl)phenyl)acetimidamide (**36**)—dual nitric oxide synthase nNOS/iNOS inhibitor **36** against glioma progression.

**Figure 42:**
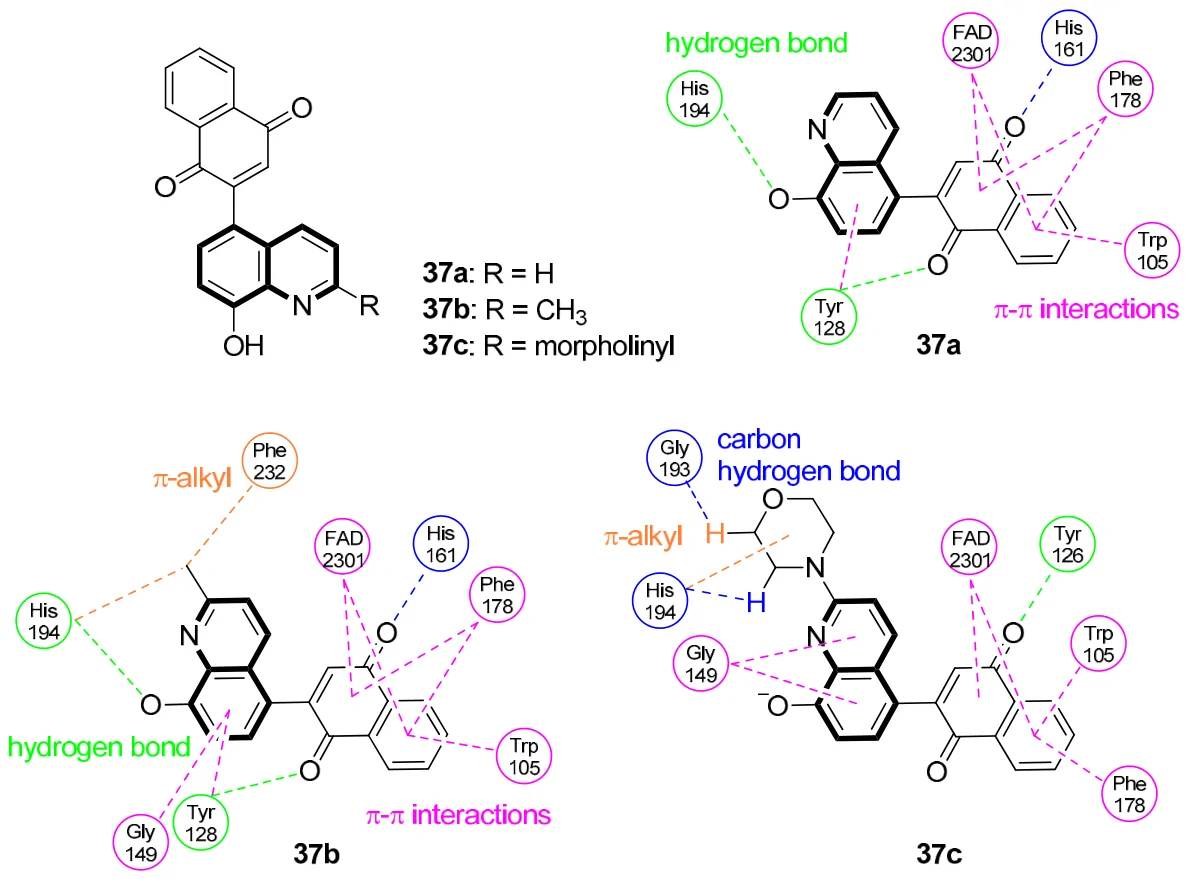
Designed by Sokal and coworkers, hybrids of 1,4-naphthoquinone with 8-hydroxyquinoline moiety **37a**, **37b**, and **37c**. Docking poses of the NQO1 protein complexed with compounds **37a**, **37b**, and **37c**.

**Figure 43:**
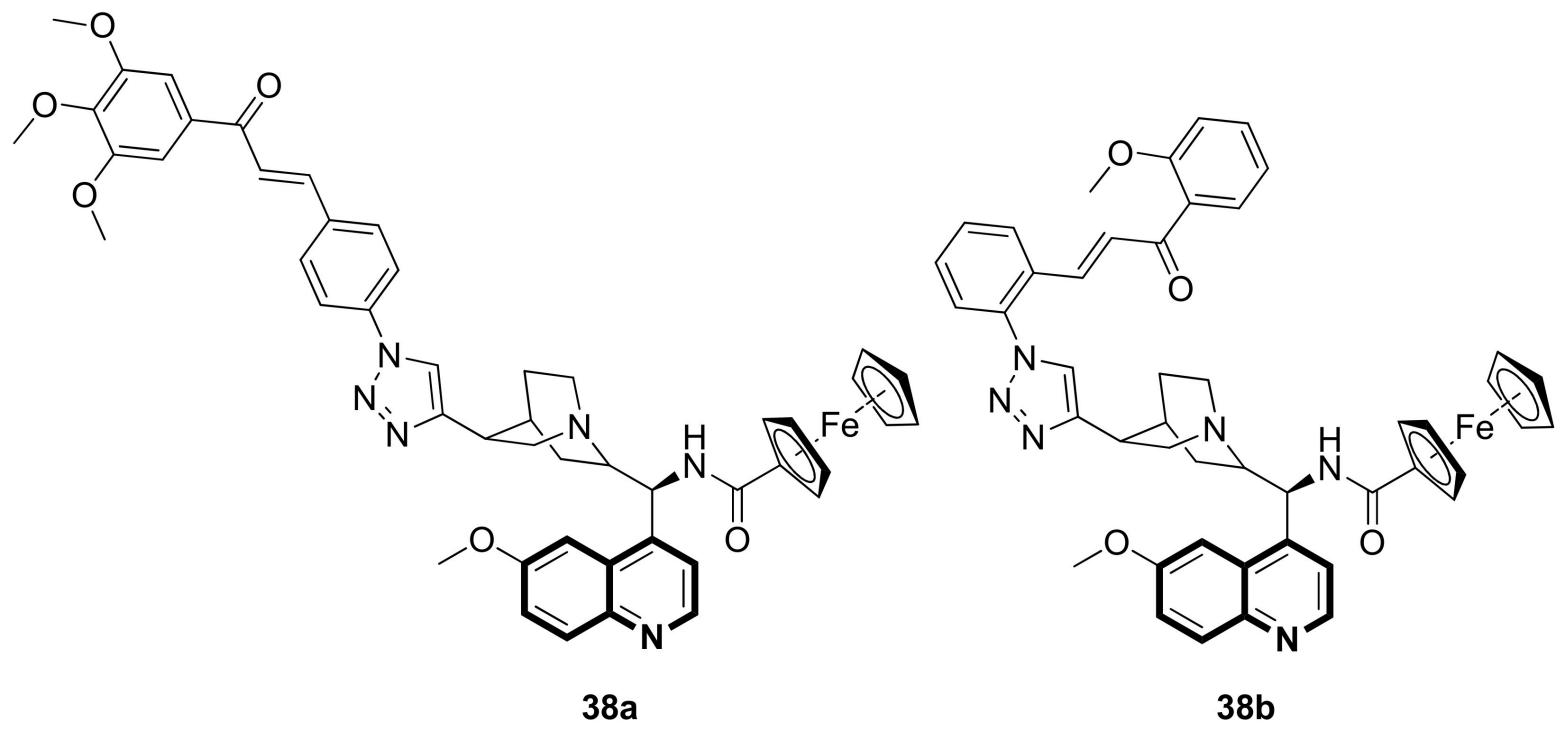
Quinoline derivatives **38a** and **38b** inducing caspase-dependent apoptosis.

**Figure 44:**
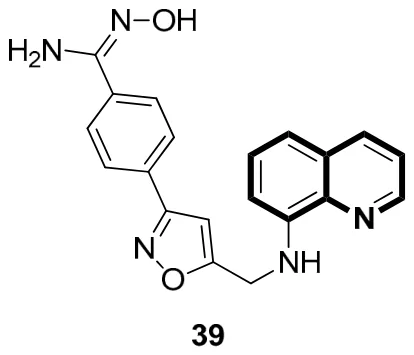
Structure of quinoline derivative **39**, a potential Bcl-2 protein inhibitor.

**Figure 45:**
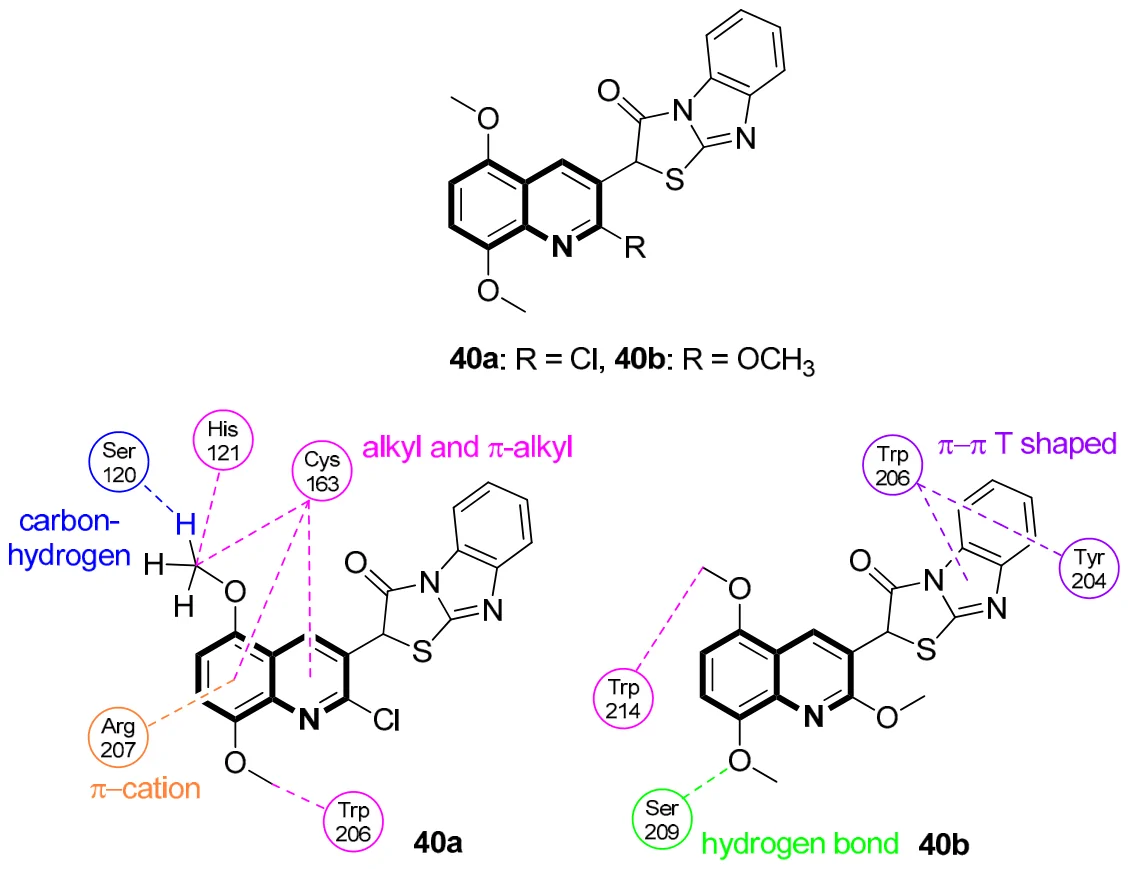
Quinoline and thiazolo[3,2-*a*]benzimidazole-3(2*H*)one hybrids **40a** and **40b** with caspase-activating activity, and their binding profiles within caspase-3.

**Figure 46:**
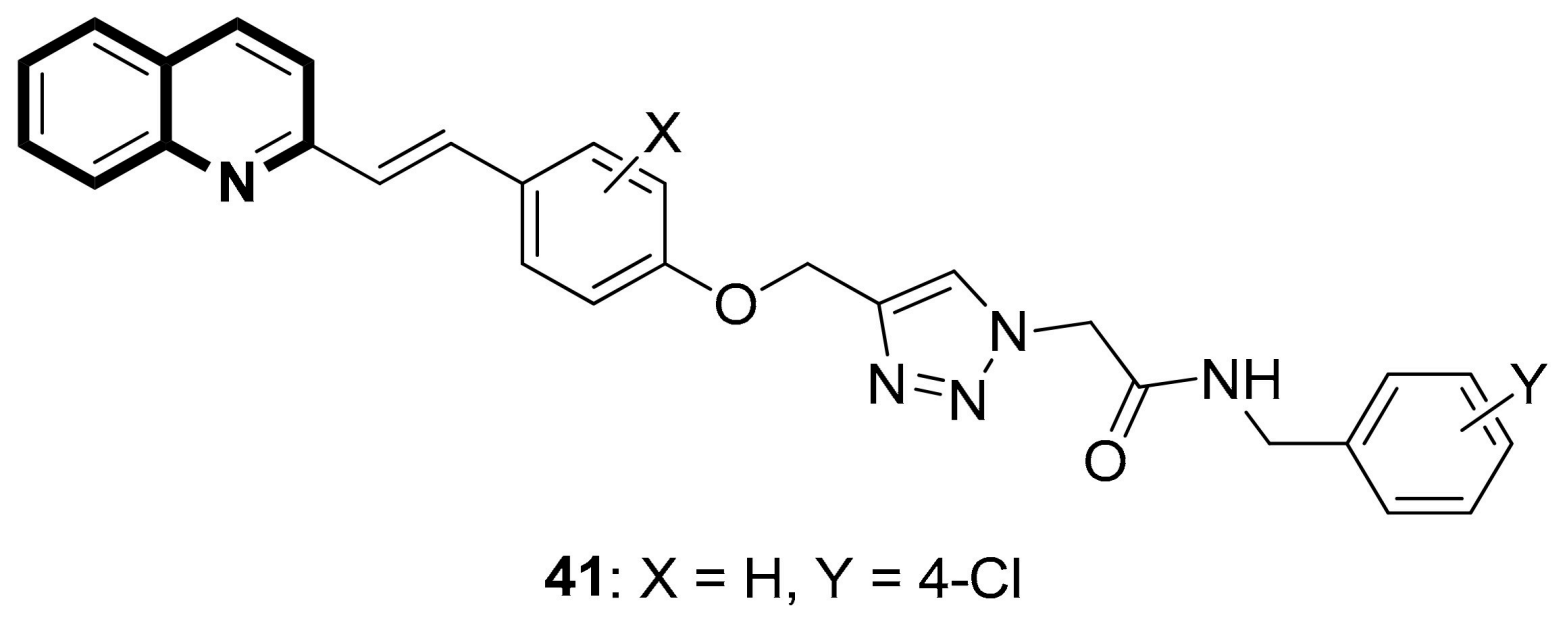
Quinoline-based chalcone **41** with cytotoxicity against MCF-7 and MDA-MB-231 cells.

**Figure 47:**
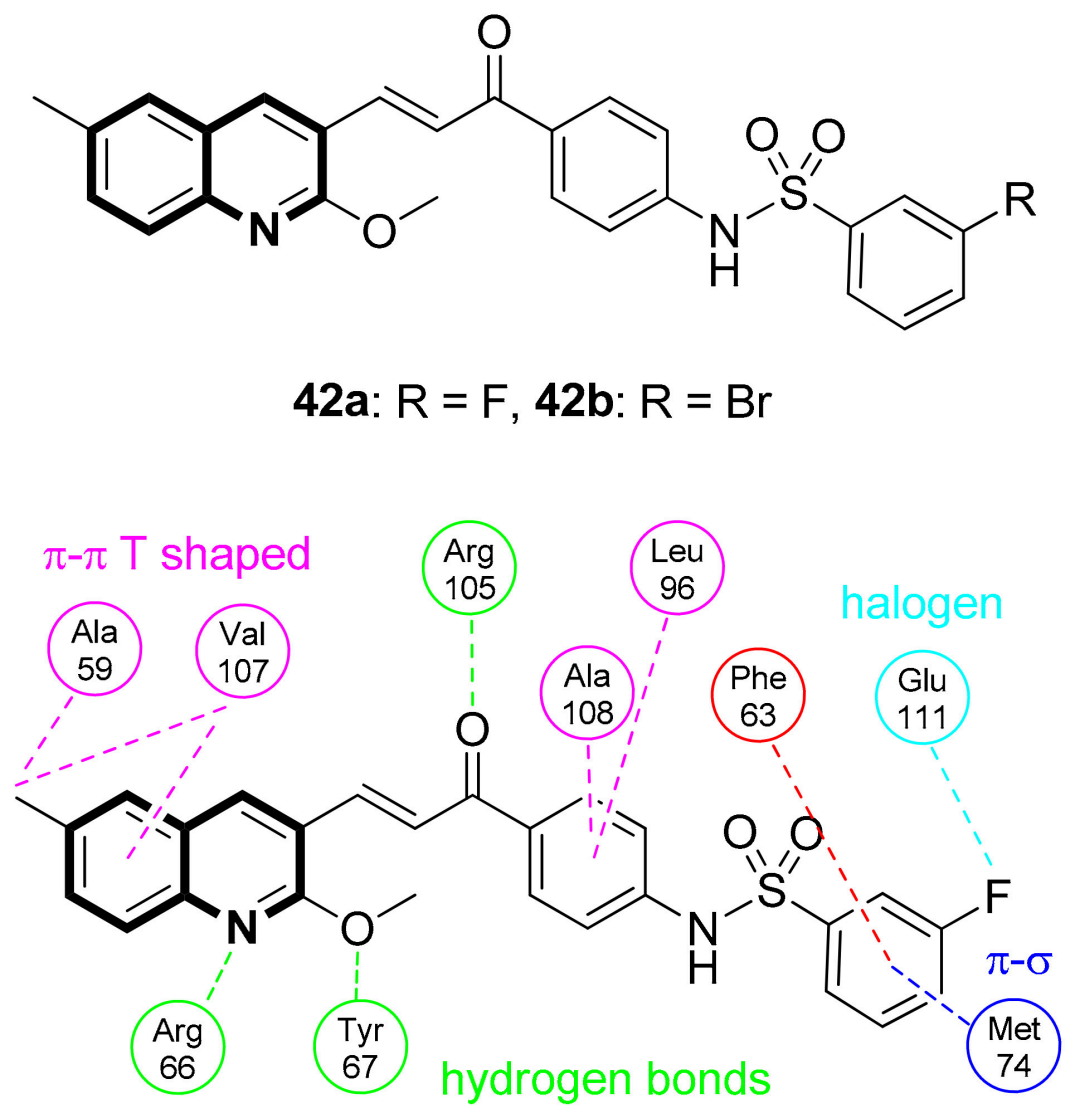
Quinoline-based Bcl-2 inhibitors/caspase activators **42a** and **42b**, and selected docking representation of **42b** with the human Bcl-2 pocket.

**Figure 48:**
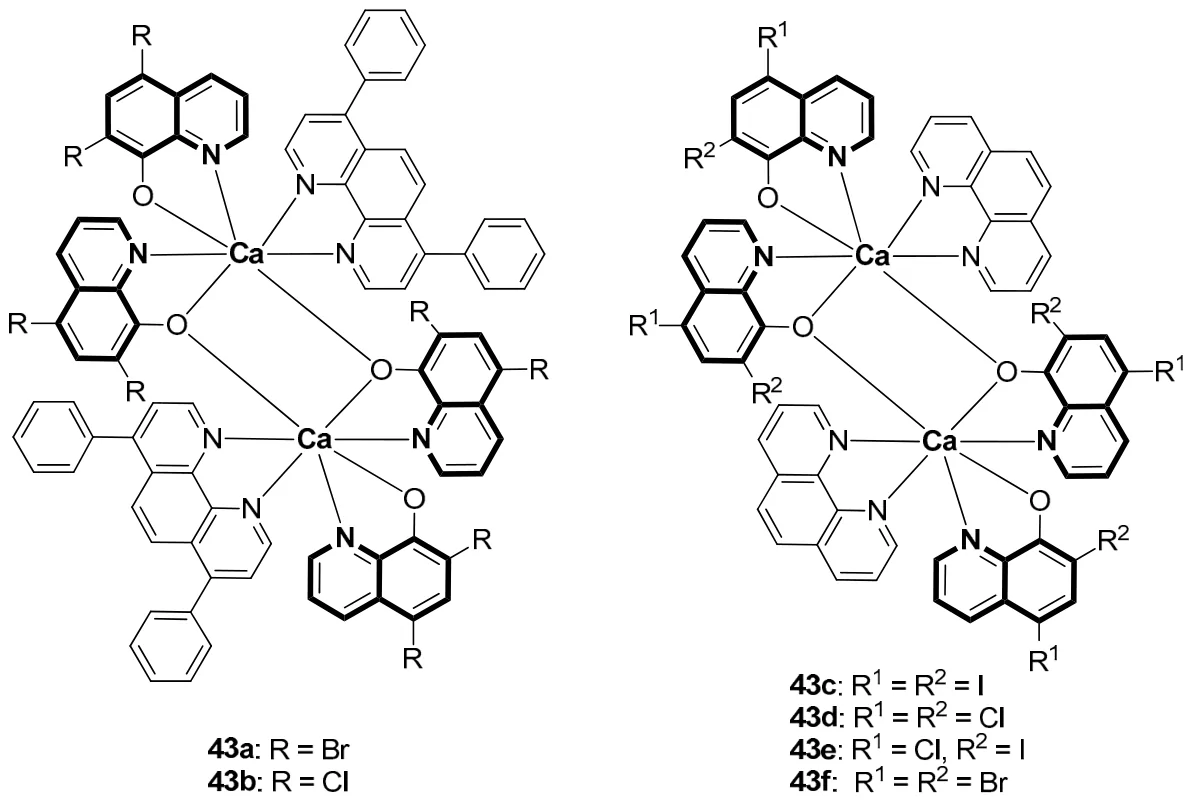
Cytotoxic calcium complexes **43a**–**43f** containing four different halogenated 8-hydroxyquinolines and 1,10-phenanthroline derivatives as auxiliary ligands.

**Figure 49:**
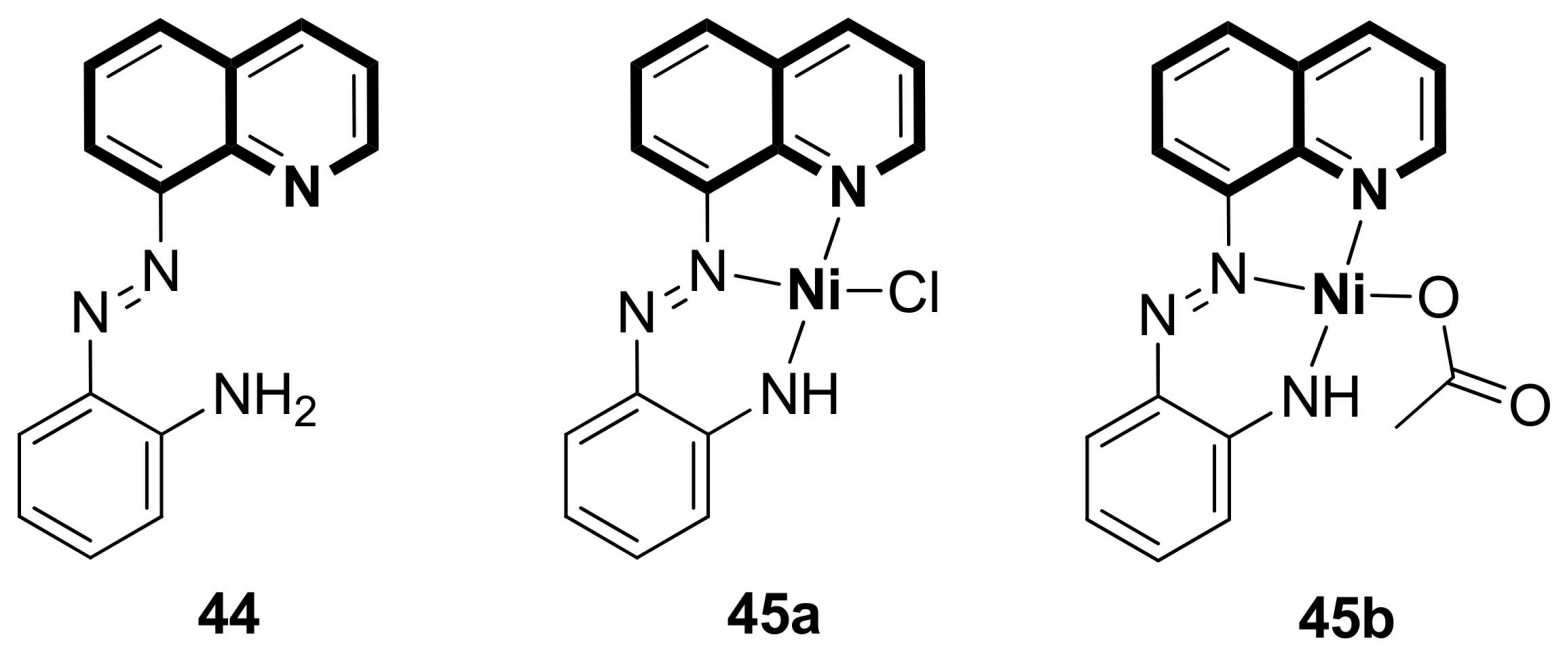
Anticancer amino-azo-quinoline ligand **44** and nickel(II) amino-azo-quinoline-based complexes **45a** and **45b**.

**Figure 50:**
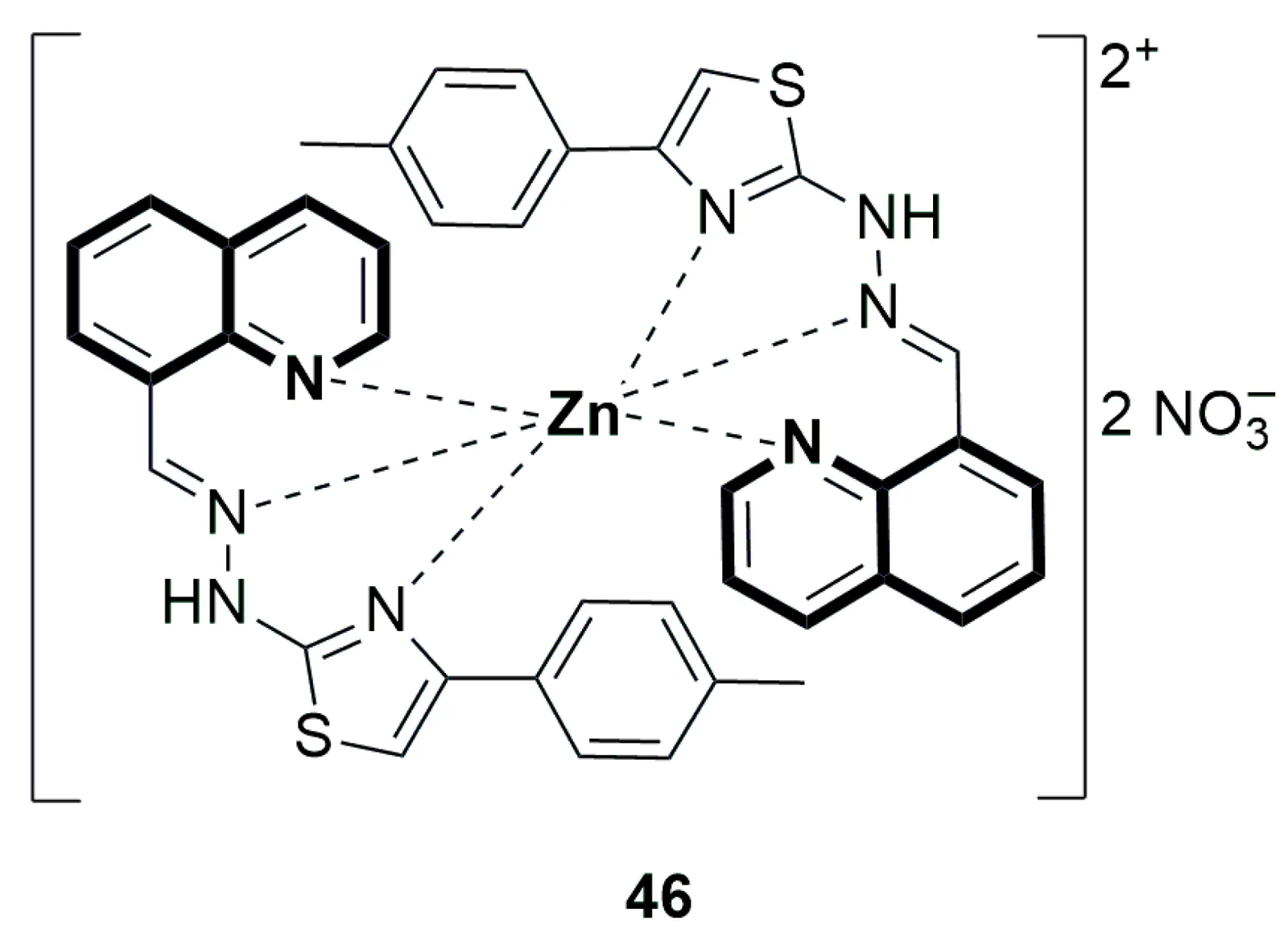
Mononuclear zinc(II) complex derived from quinolone-based ligand **46** [Zn(HL)_2_](NO_3_)_2_ with pronounced anticancer activity.

**Figure 51:**
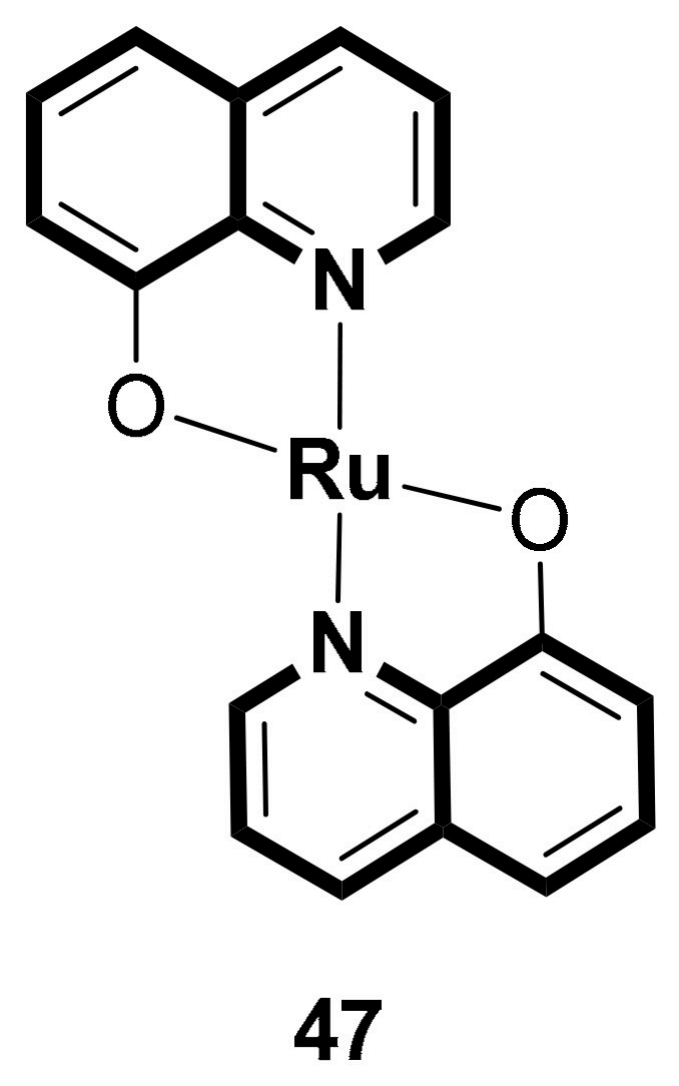
Quinoline-based Ru(II) complex Ru(quin)_2_ **47**—an effective anticancer agent against ER+ and TNBC cells.

**Figure 52:**
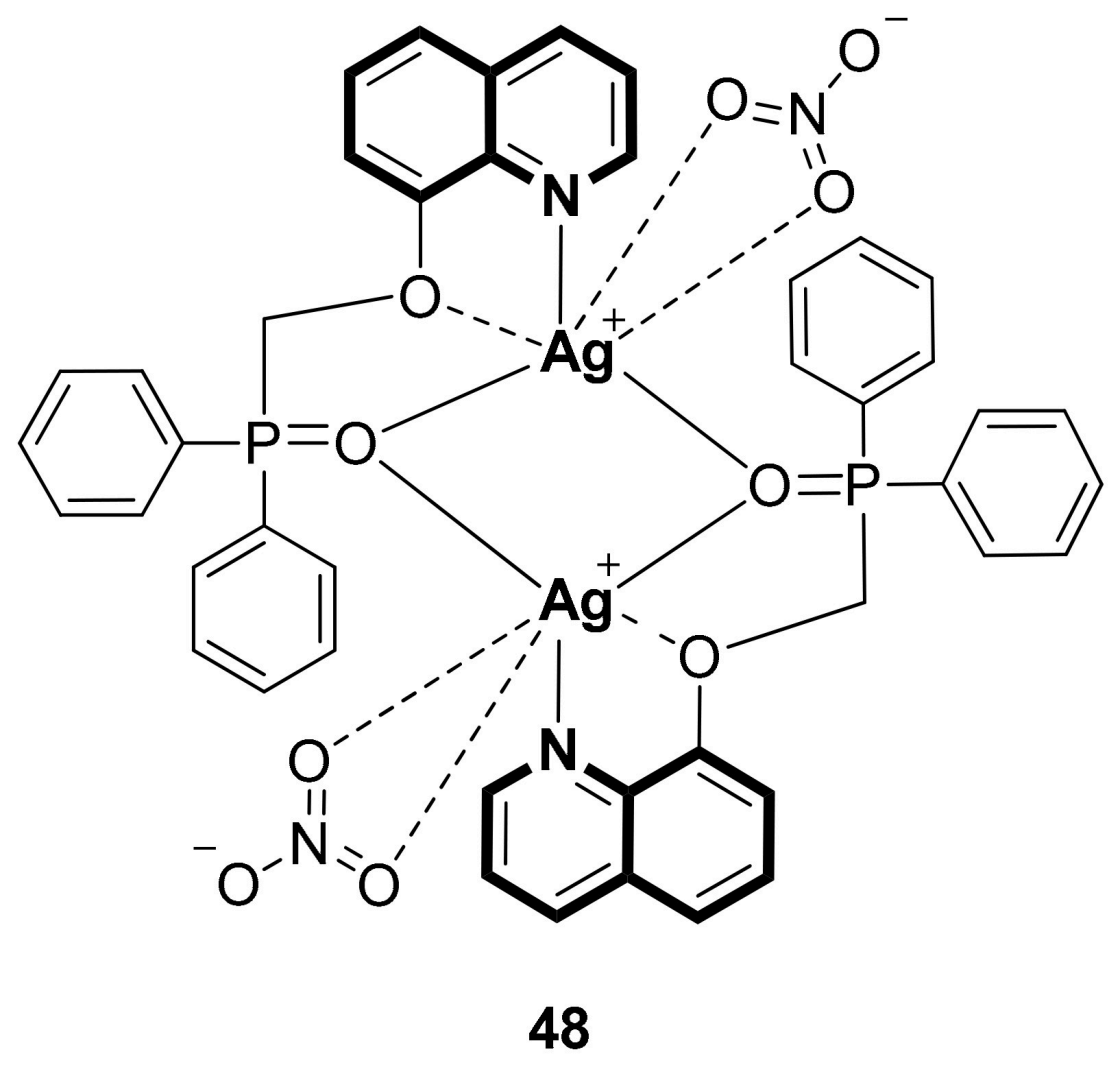
Silver(I) complex **48** [AgNO_3_L]_2_ with cytotoxic properties.

**Figure 53:**
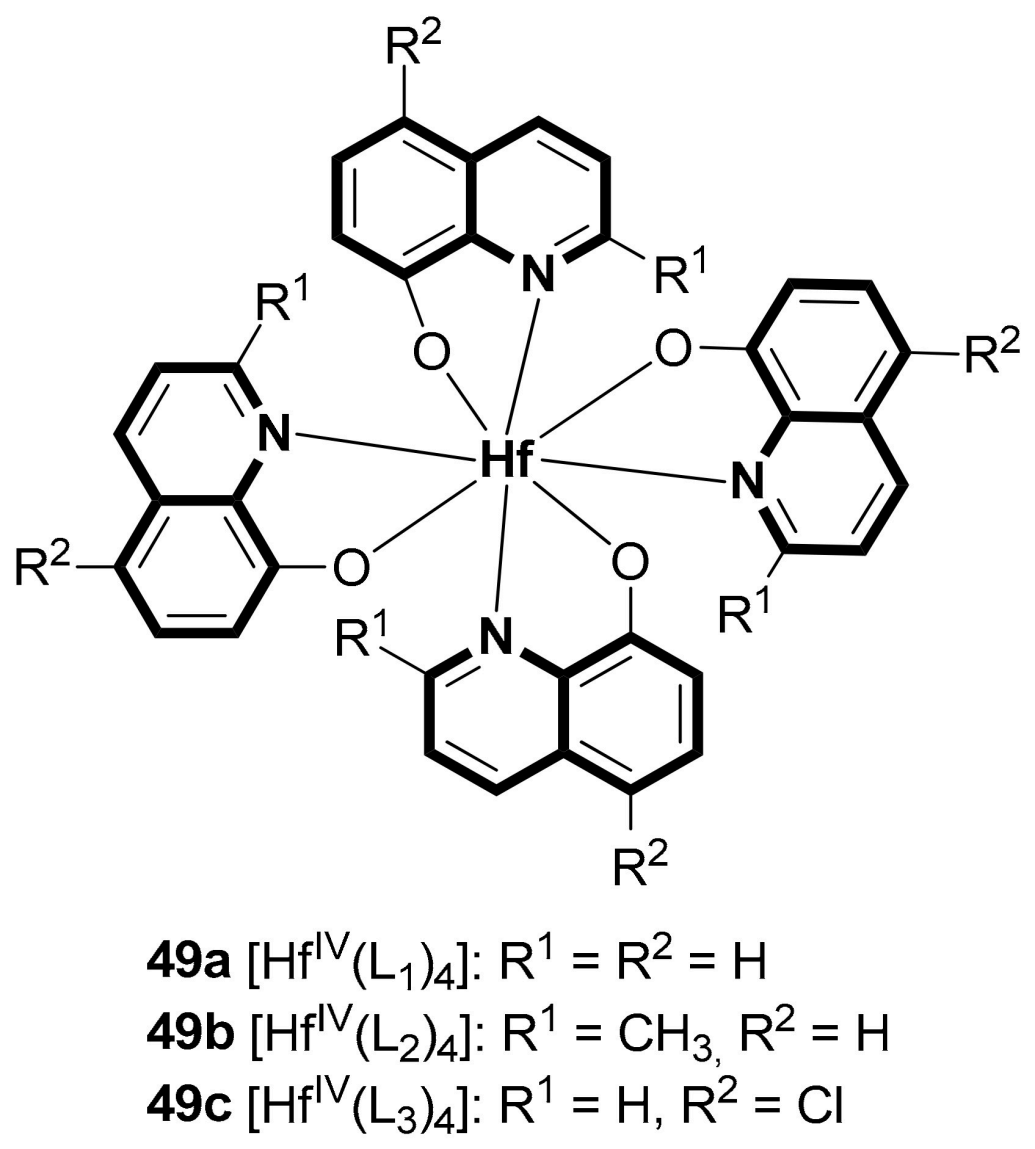
Hafnium(IV) complexes **49a**, **49b**, and **49c** with proven anticancer properties.

**Figure 54:**
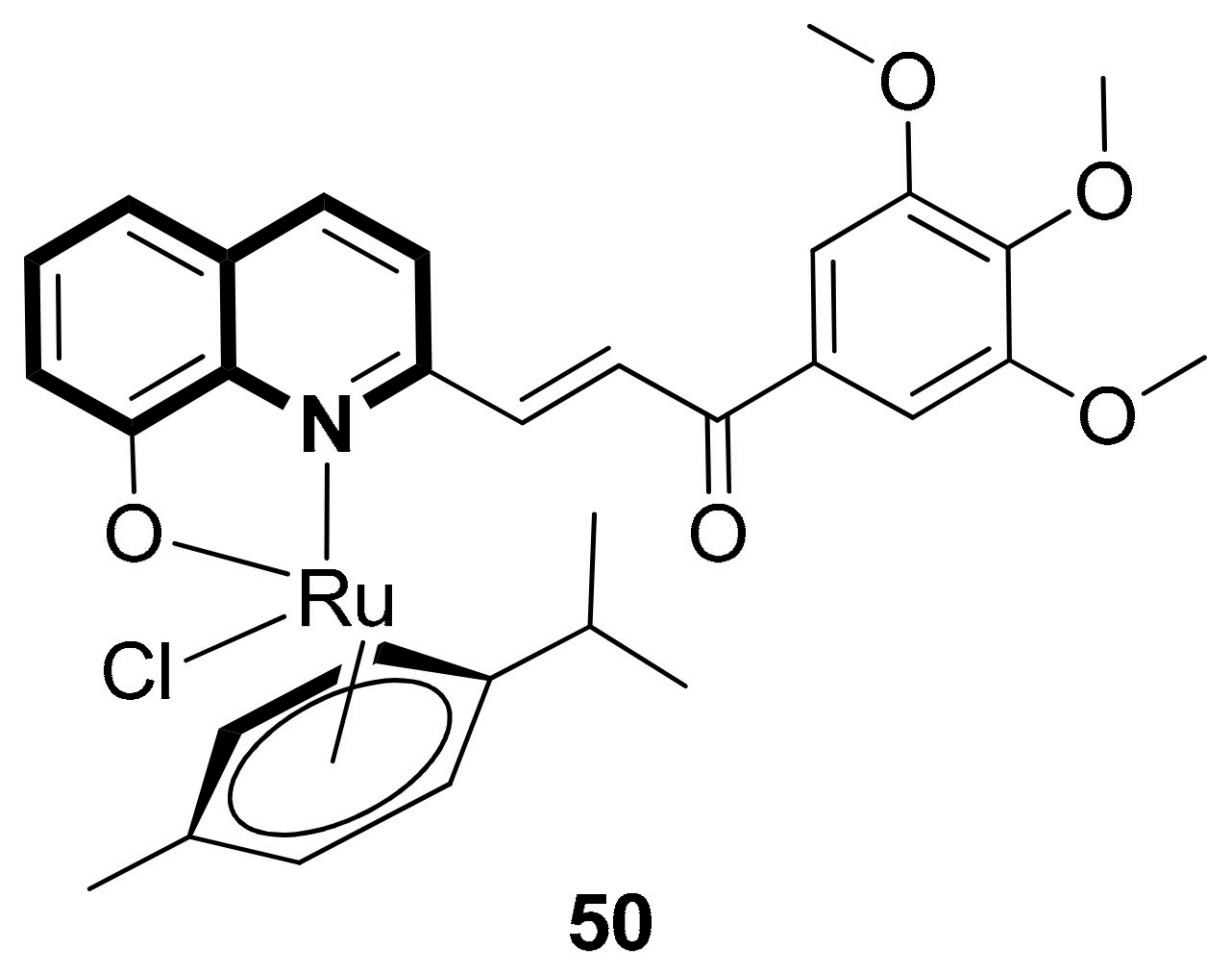
Ruthenium(II) complex **50** with anticancer and anti-inflammatory properties.

**Table 1:** Examples of valuable quinoline-based compounds with anticancer activity, and their molecular targets with study type and biological model.

Structure	Molecular Target/ Study Type/Biological Model	Number	Ref.
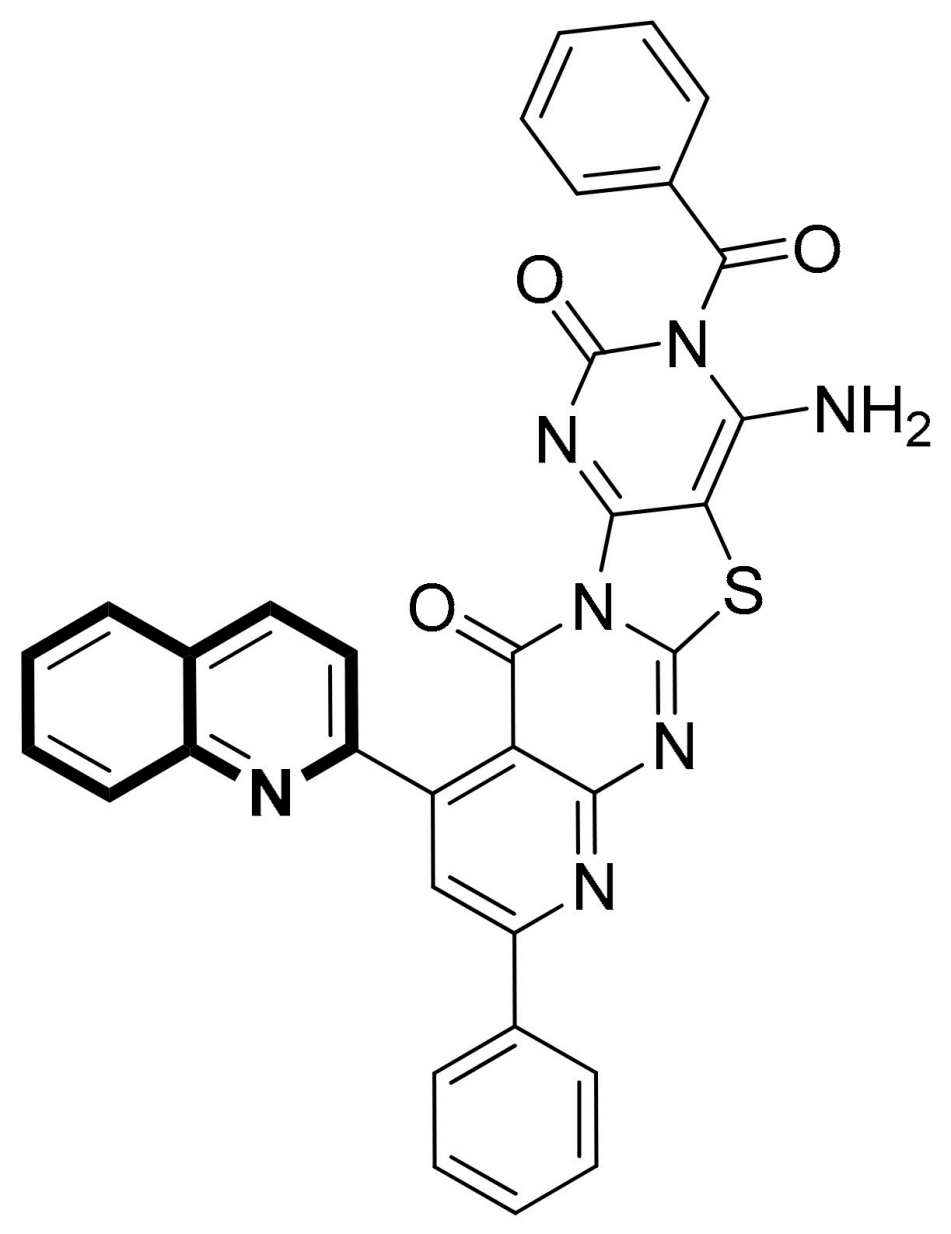	EGFR In vitro: Cervical cancer HeLa (oral carcinoma KB), nasopharyngeal carcinoma CNE2, gastric carcinoma MGC-803, and breast adenocarcinoma MCF-7 cell lines (enzymatic EGFR inhibition and cellular antiproliferative profiling) In silico: Molecular docking into the EGFR ATP-binding domain, ADMET profile prediction, and molecular dynamics (MD) trajectory stability simulations	**3b**	[86]
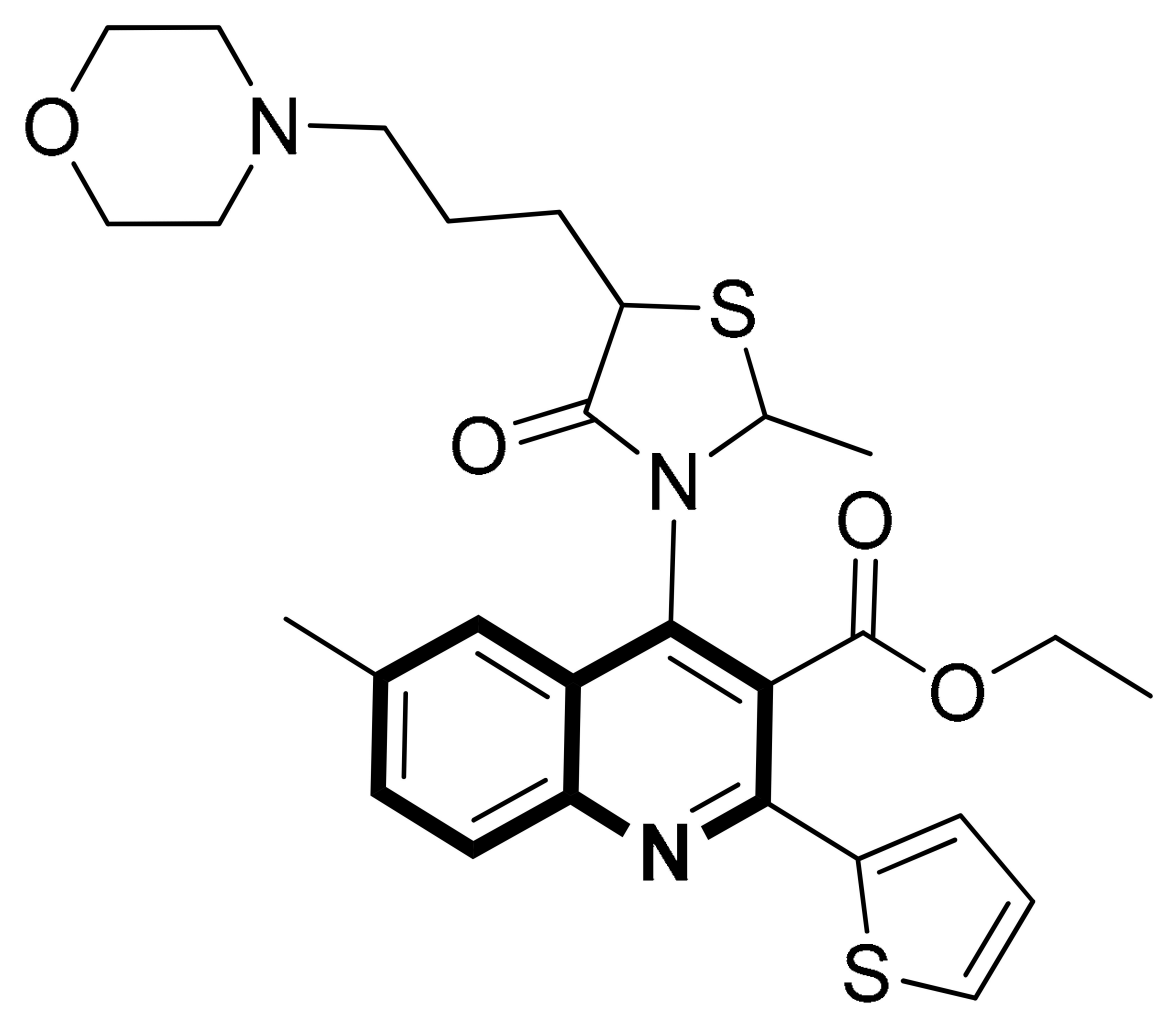	VEGFR-2 In vitro: Human hepatoblastoma HepG-2, MCF-7, and HCT-116 cell lines (cytotoxicity, antiproliferative, and anti-angiogenic evaluation) In silico: Computer-Aided Drug Design (CADD), computational virtual screening, and molecular docking within the VEGFR-2 kinase catalytic domain	**4**	[87]
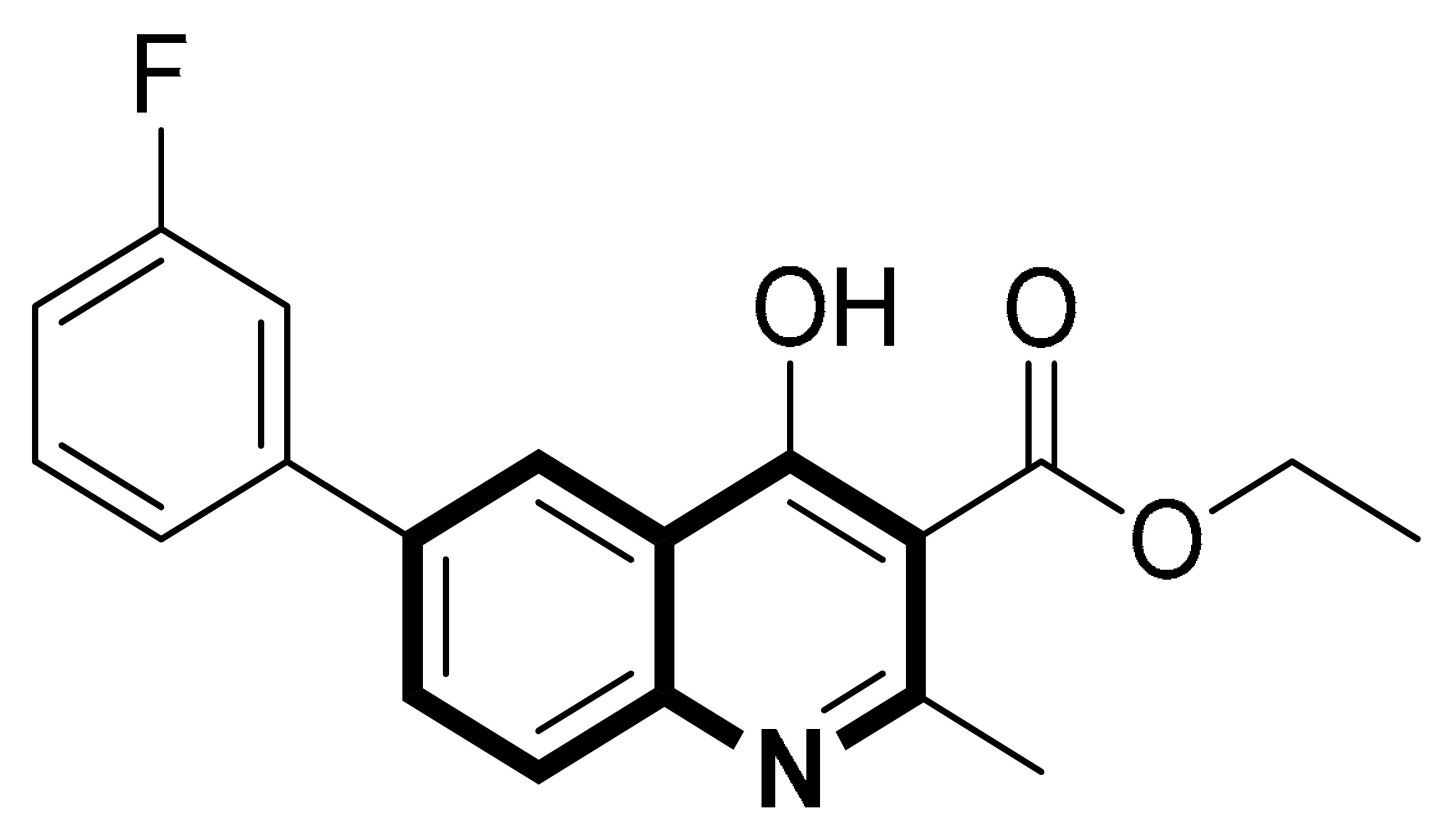	CK2 and VEGFR2 In vitro: Triple-negative breast cancer (TNBC) MDA-MB-468 cells evaluated in 2D monolayer and 3D tumor spheroid models (dual CK2/VEGFR2 multi-kinase inhibition, ROS accumulation, and 3D spheroid volume reduction)	**7a**	[91]
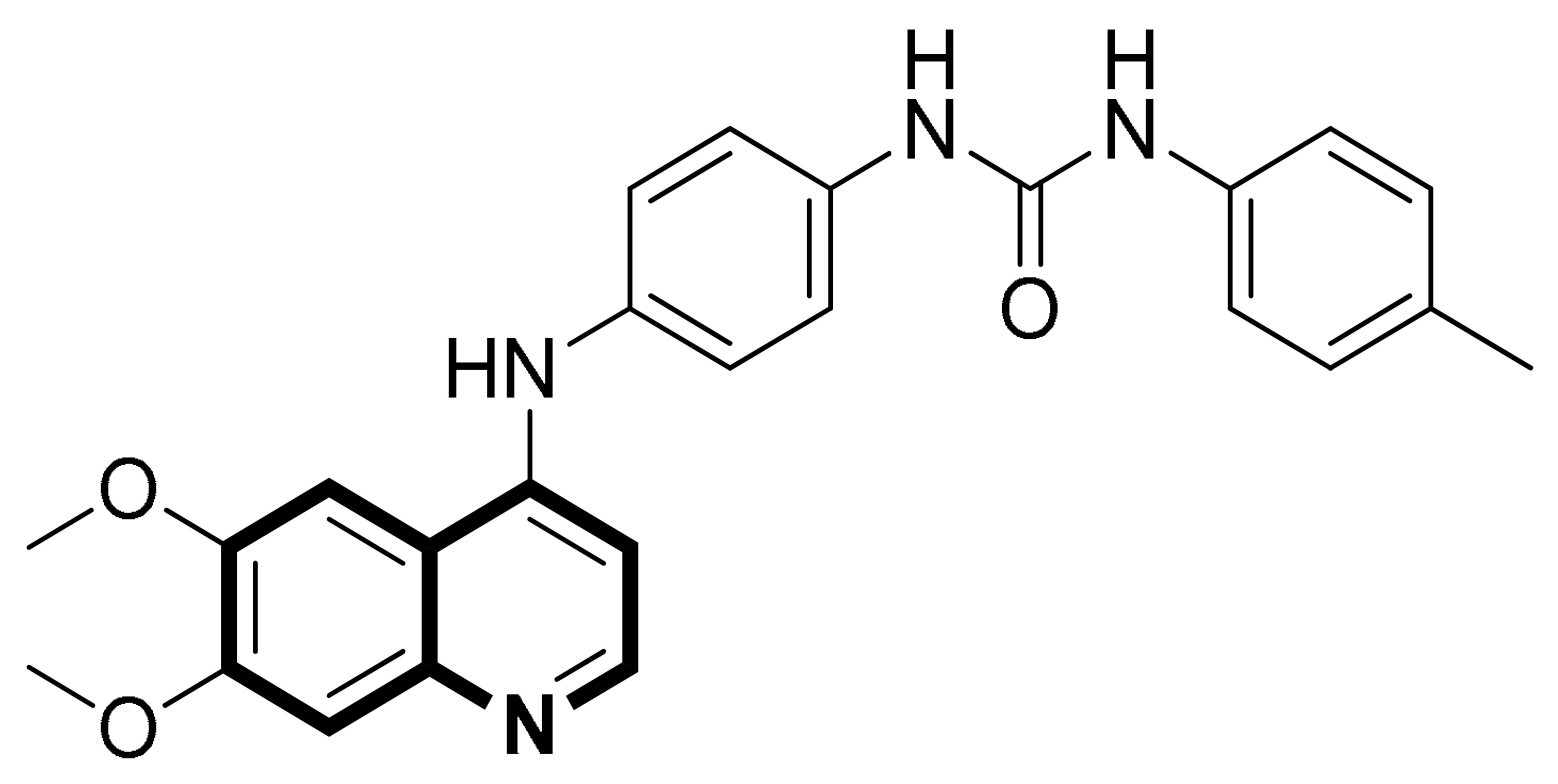	PDGFR-α In vitro: Human colon cancer HT-29 cell line (enzymatic PDGFR-α kinase inhibition and cellular anti-proliferative activity)	**8**	[94]
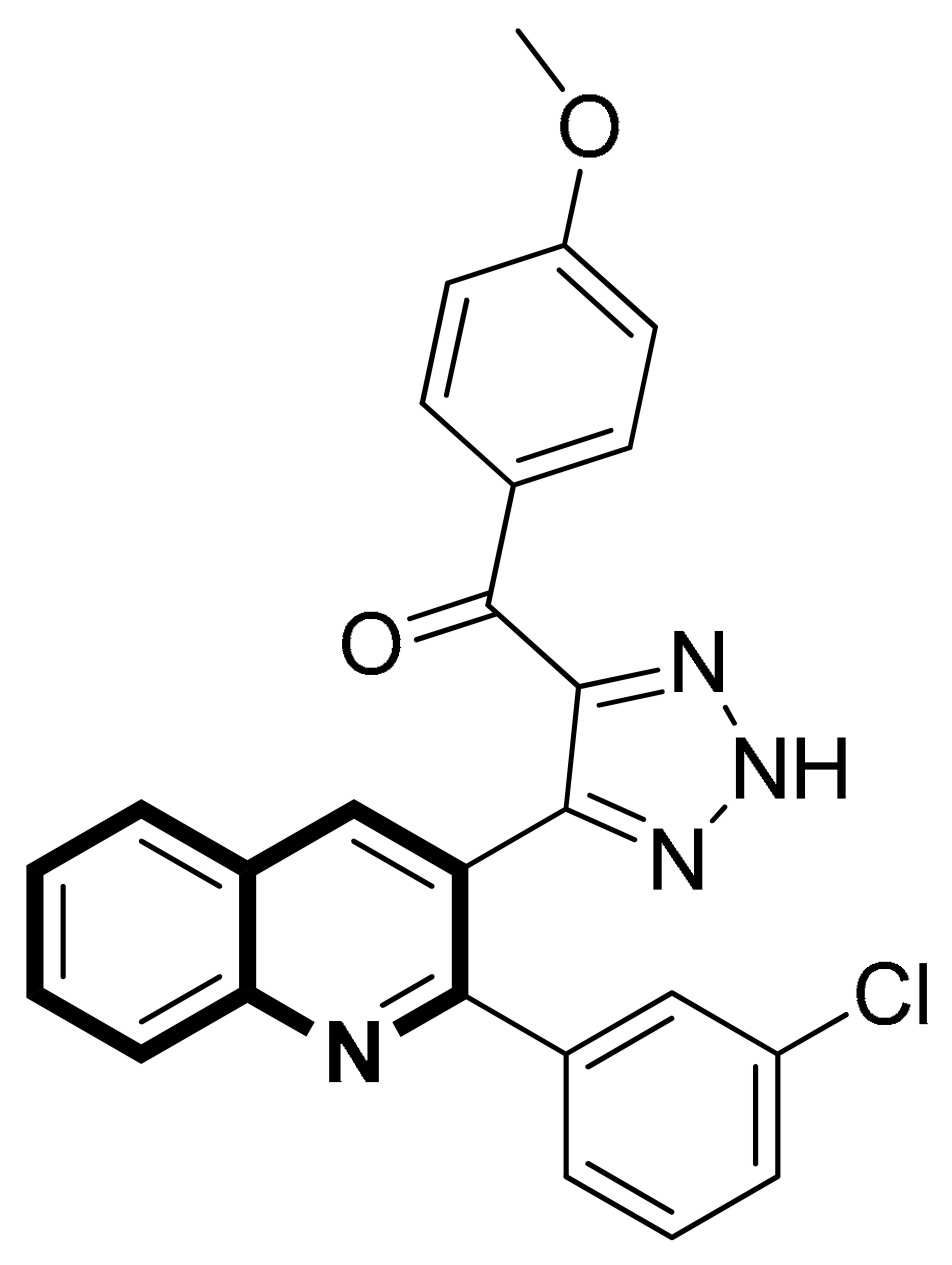	FGFR1 In vitro: Breast adenocarcinoma MCF-7, lung adenocarcinoma A-549, hepatoblastoma HepG2 cell lines (cytotoxicity and antiproliferative assays) In silico: Molecular docking against FGFR1 catalytic site, binding interaction analysis, and ADME/drug-likeness profiling	**9d**	[95]
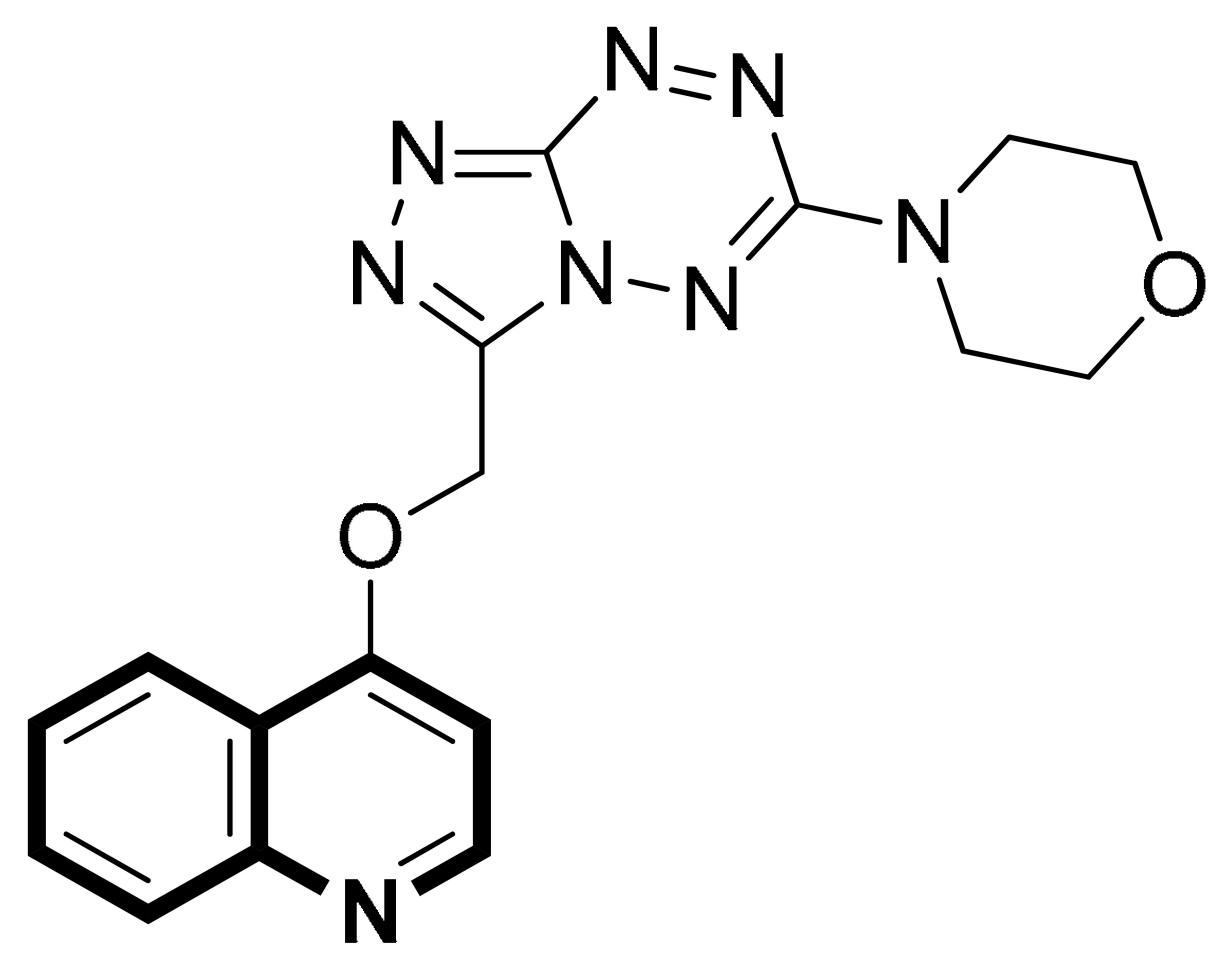	c-Met kinase In vitro: Human lung adenocarcinoma A549 and large cell lung carcinoma H460 cell lines (cytotoxicity, antiproliferative assays, and kinase inhibition profiling) In silico: Molecular docking into the c-Met ATP-binding pocket to analyze hinge region binding, H-bonding, and hydrophobic interactions	**10a**	[99]
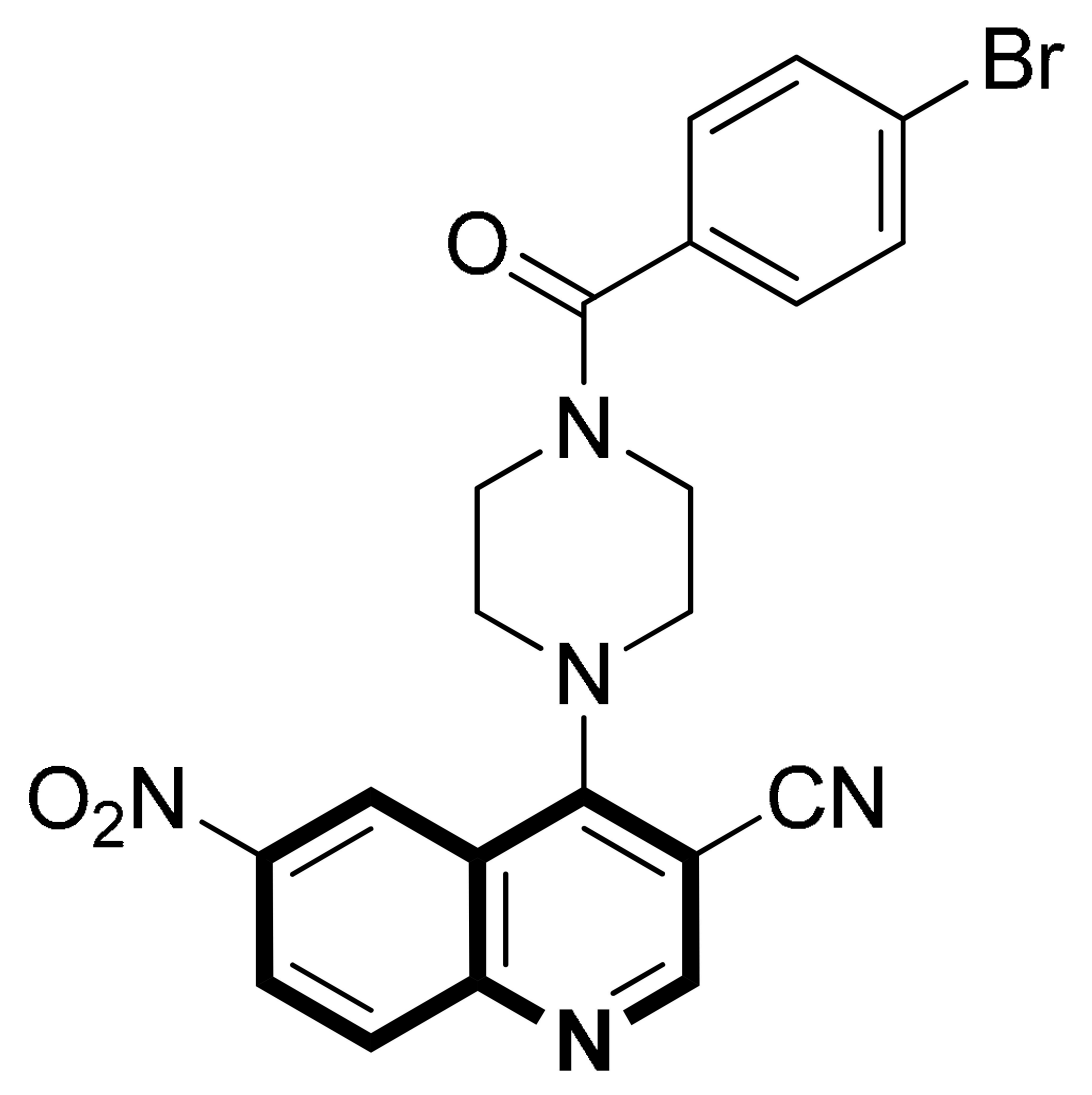	AXL receptor tyrosine kinase In vitro: Renal cell carcinoma UO-31, non-small cell lung cancer (NSCLC), CNS cancer, and breast cancer cell lines (enzymatic AXL inhibition, antiproliferative profiling, and anti-migratory evaluation) In silico: Structure-based molecular docking into the AXL kinase domain, key binding interaction mapping, and SAR profiling	**11b**	[100]
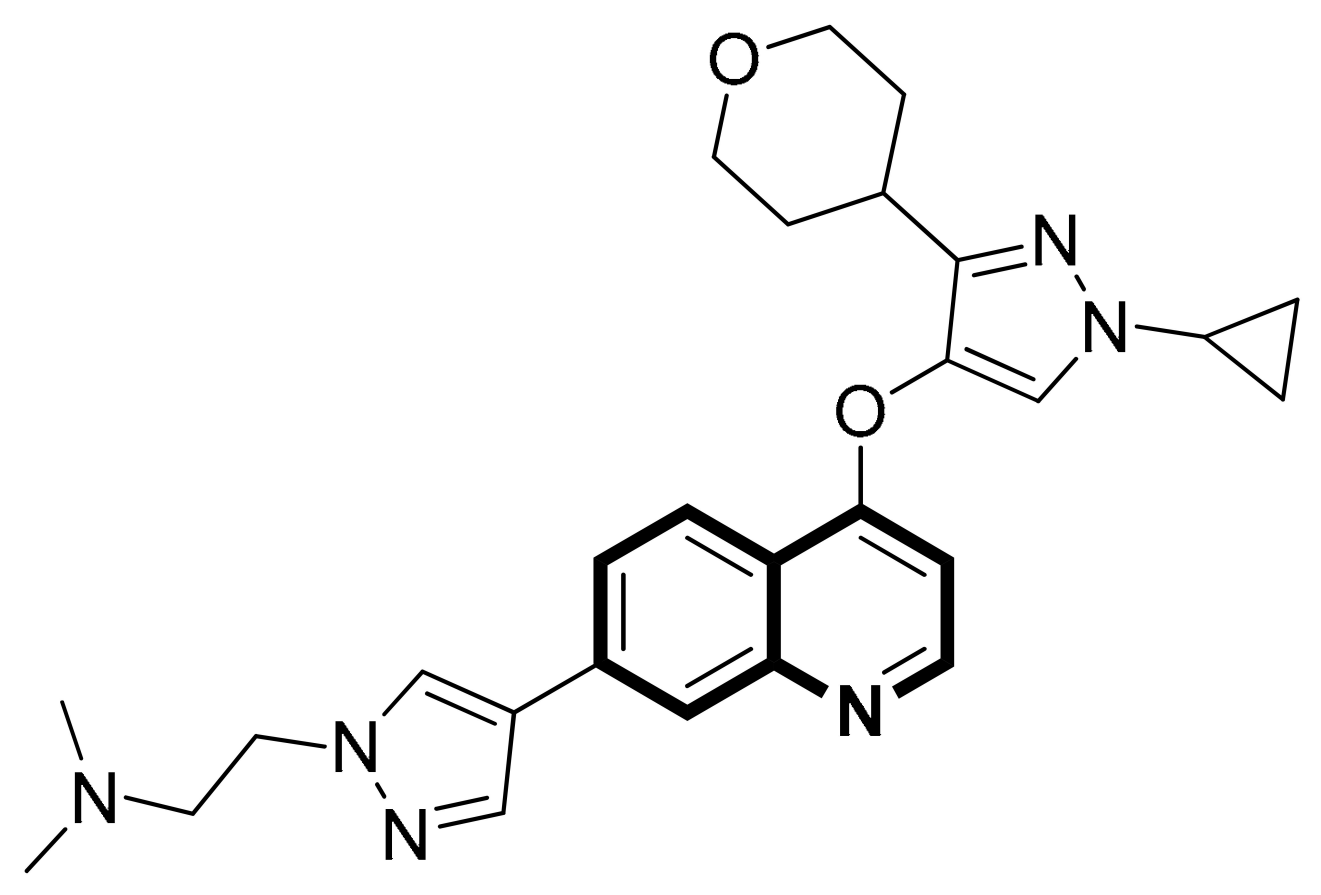	TGFβR1 In vitro: Mouse H22 hepatoma xenograft model (tumor volume reduction, survival prolongation, and antitumor efficacy)	**12**	[102]
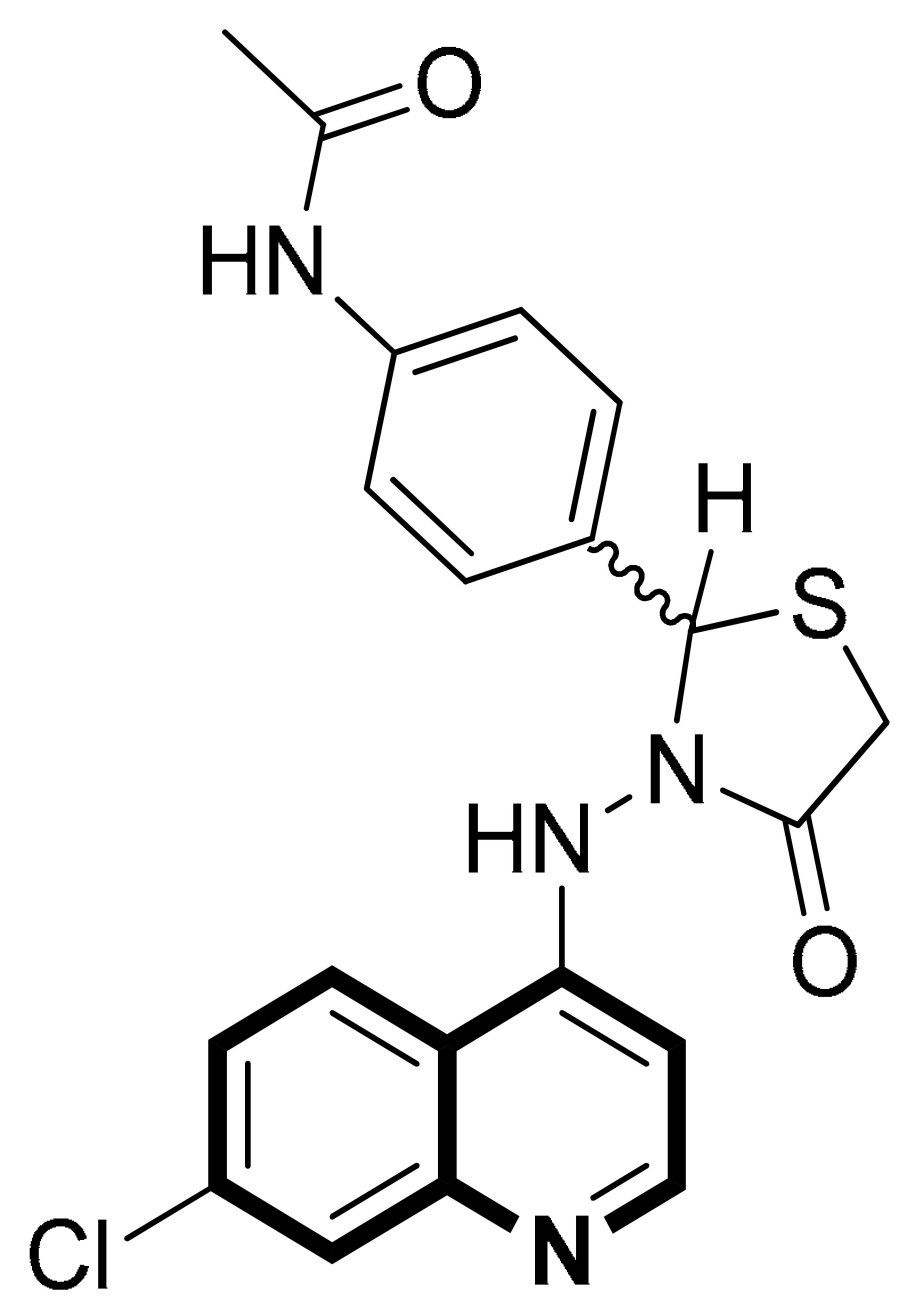	TLR4-LPS axis In vitro: Human breast adenocarcinoma MCF-7 and triple-negative breast cancer MDA-MB-231 cell lines (inhibition of LPS-induced TLR4 signaling, suppression of cell migration, and Transwell invasion)	**13**	[103]
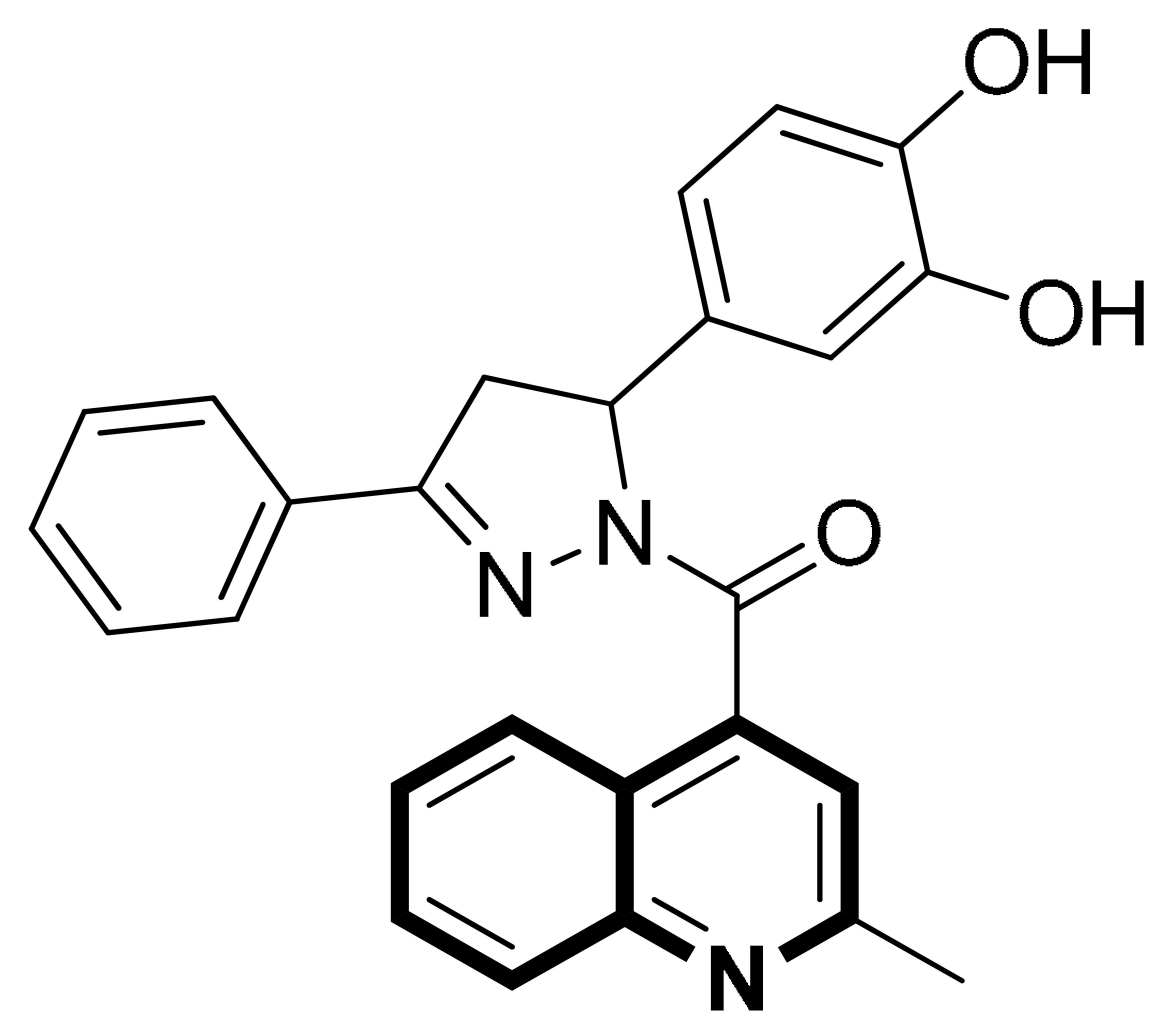	STAT3 In vitro: Human lung adenocarcinoma A549 cell line (cytotoxicity and anti-proliferative assays) In silico: 3D-QSAR modeling, molecular docking into the STAT3 SH2 domain, molecular dynamics (MD) simulations, and DFT quantum chemical analysis	**16d**	[109]
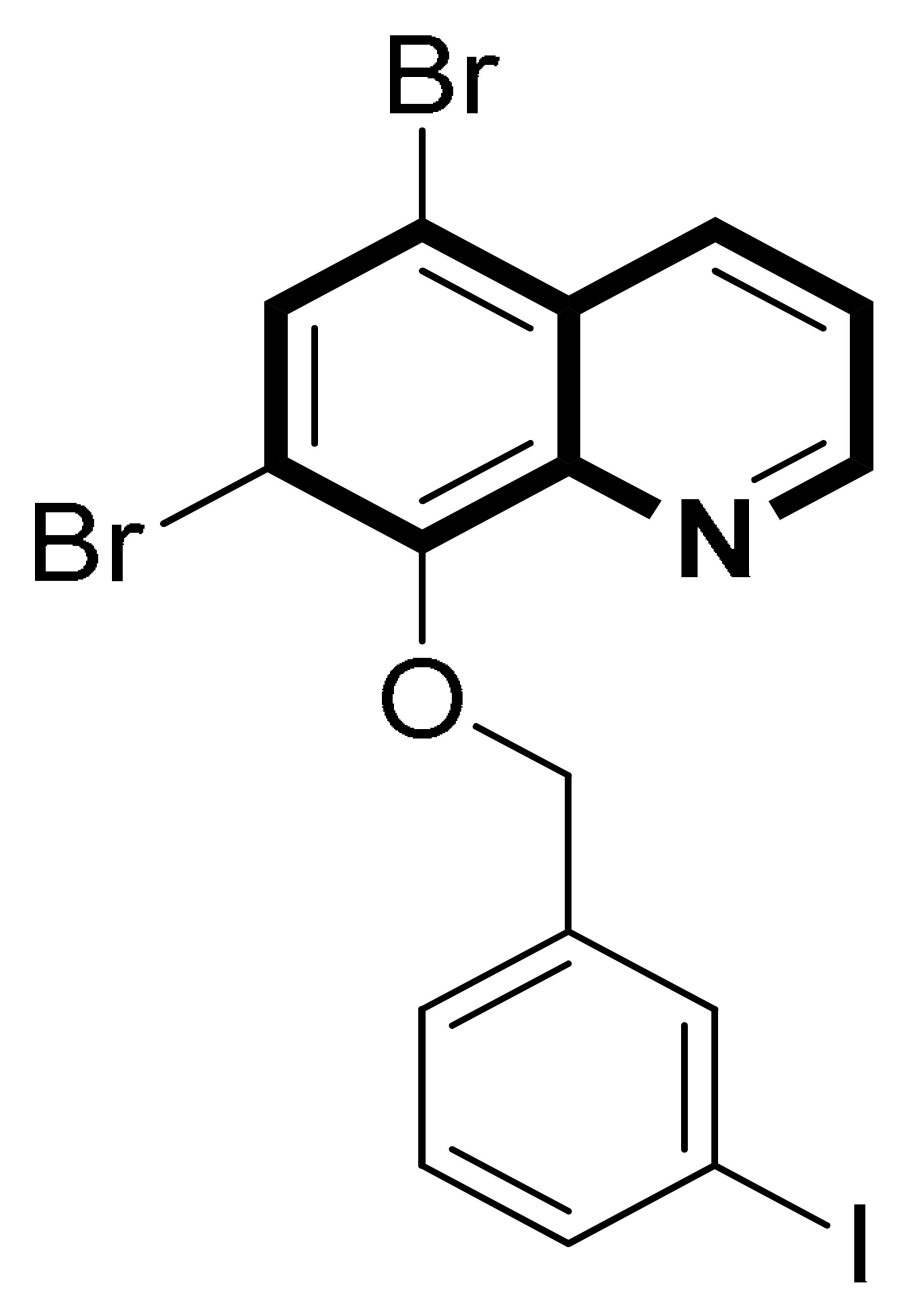	MKK7-JNK signaling pathway In vitro: Caspase-dependent apoptosis, triple-negative breast cancer (TNBC) MDA-MB-468 cells (induction of caspase-dependent apoptosis and G2/M phase arrest via MKK7-JNK phosphorylation)	**17b**	[112]
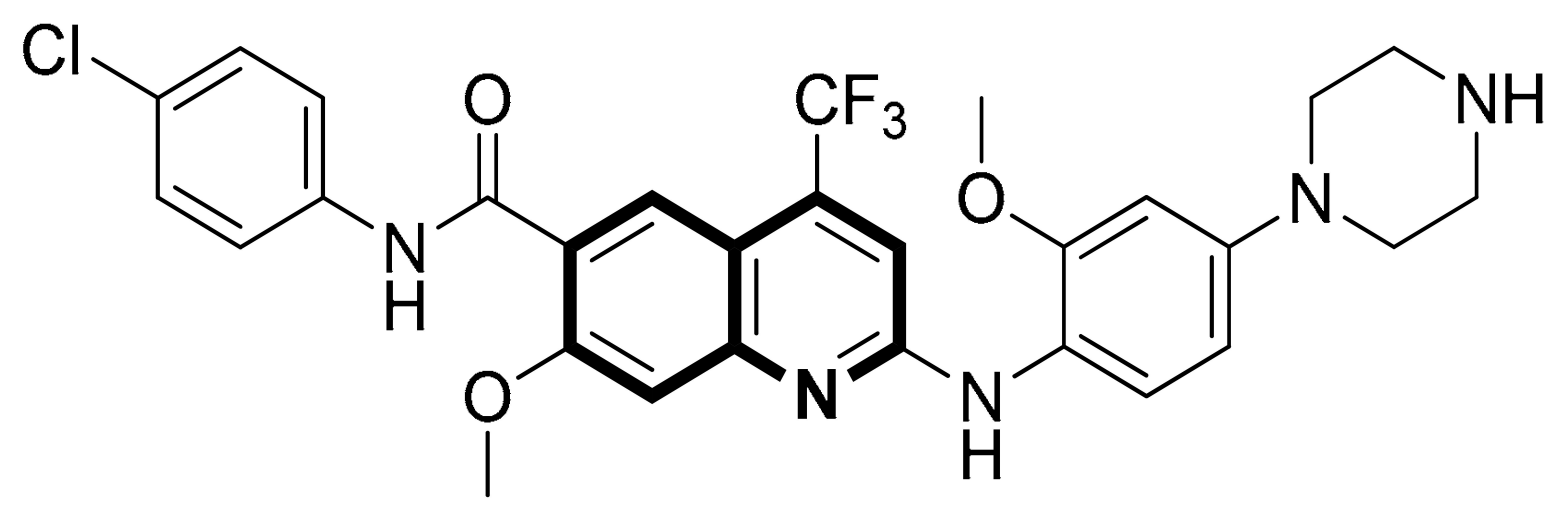	SGK1 In vitro: Prostate cancer PC3, androgen-sensitive prostate adenocarcinoma LNCaP In vivo: Mouse tumor xenograft models of prostate cancer progression	**19**	[117,118]
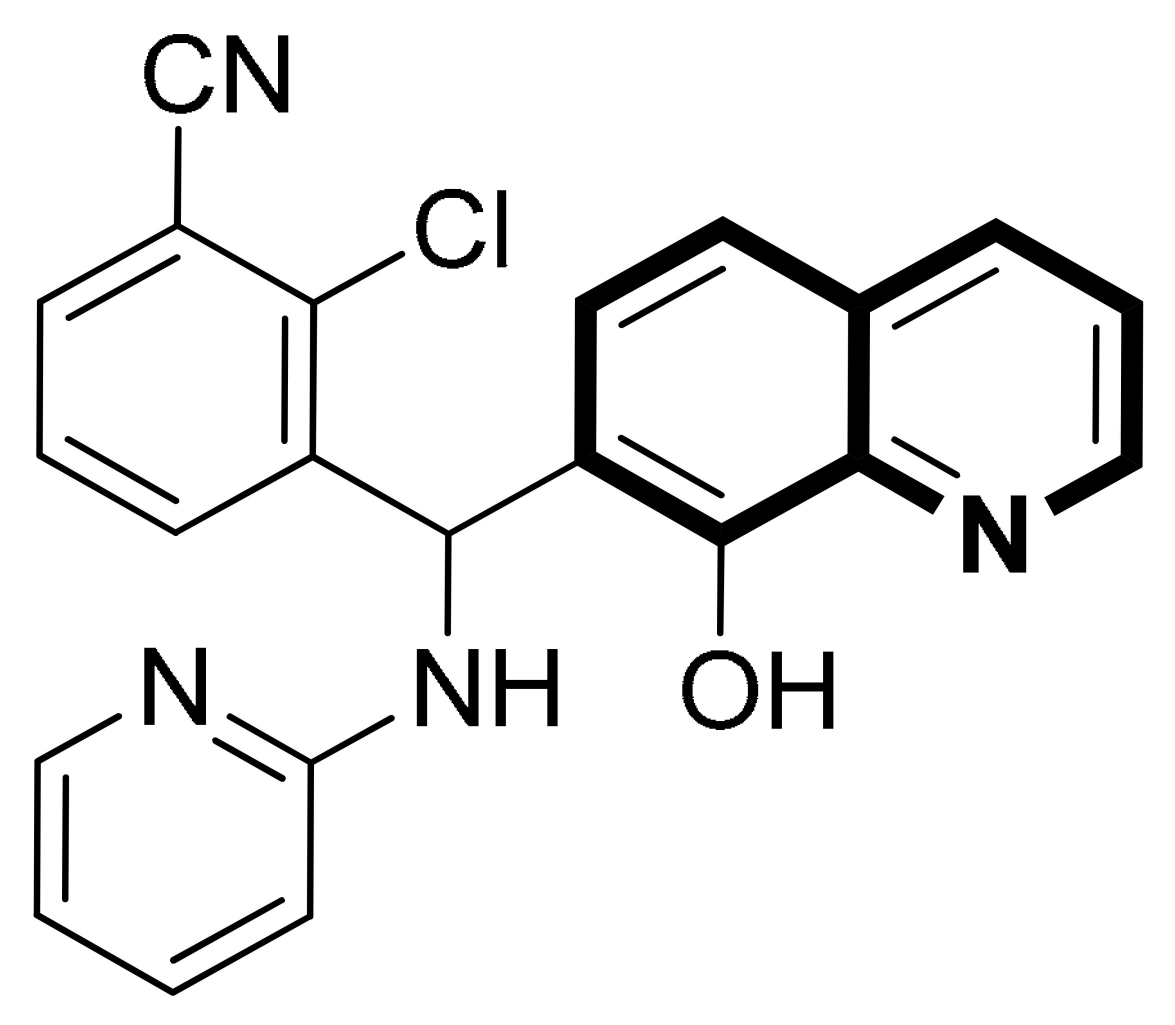	SGK1/MDM2-MDM4 complex In vitro: Melanoma A375 cells (including BRAF inhibitor-resistant models; inhibition of MDM2–MDM4 E3 ligase complex, suppression of survival pathways, and promotion of p53-independent cell death)	**21b**	[122]
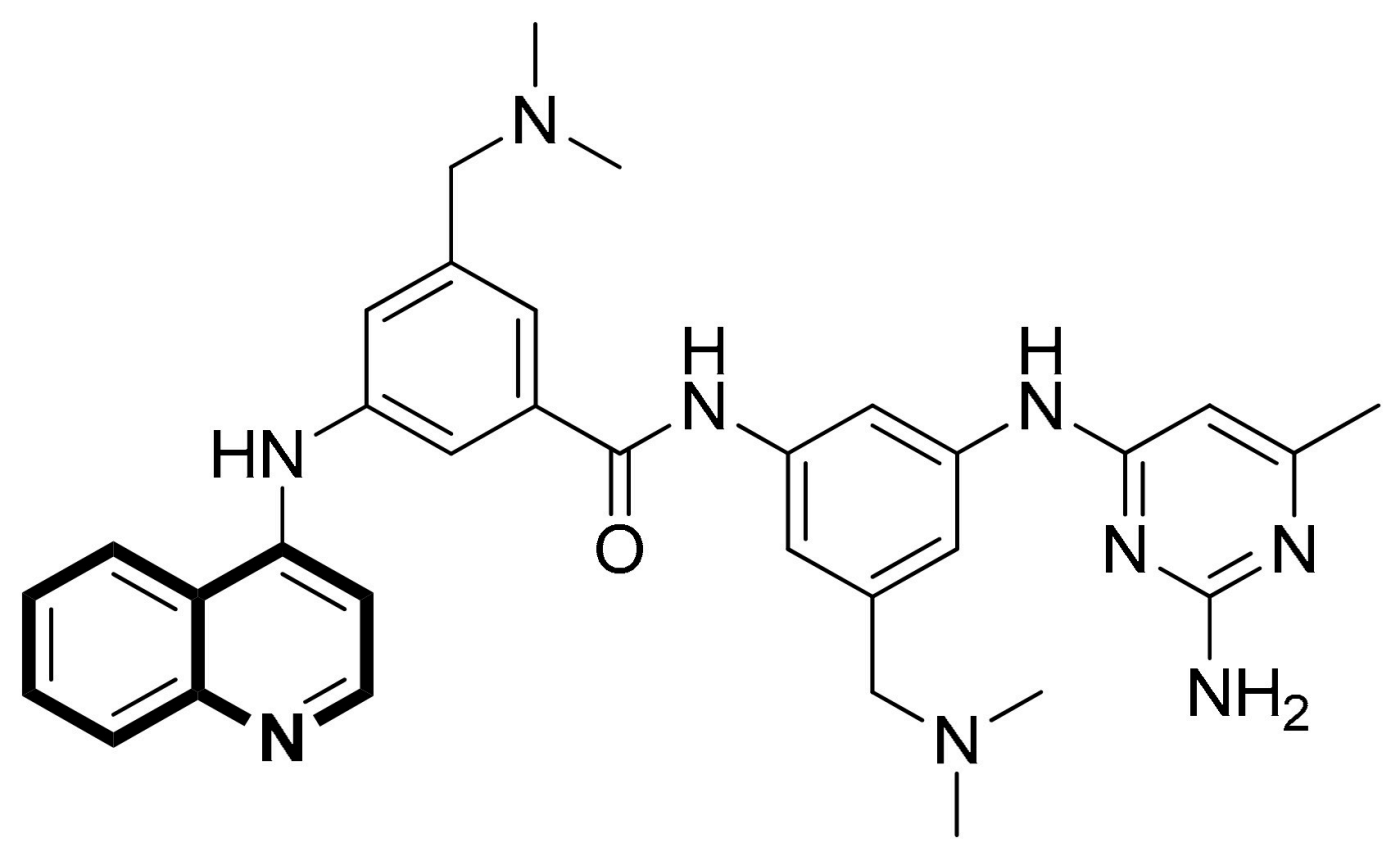	DNMT1 In vitro: Colon cancer (HCT-116) cells (including p53 models; selective enzymatic DNMT1 inhibition, DNA hypomethylation, p53-dependent apoptosis, and DNA damage induction)	**22b**	[126]
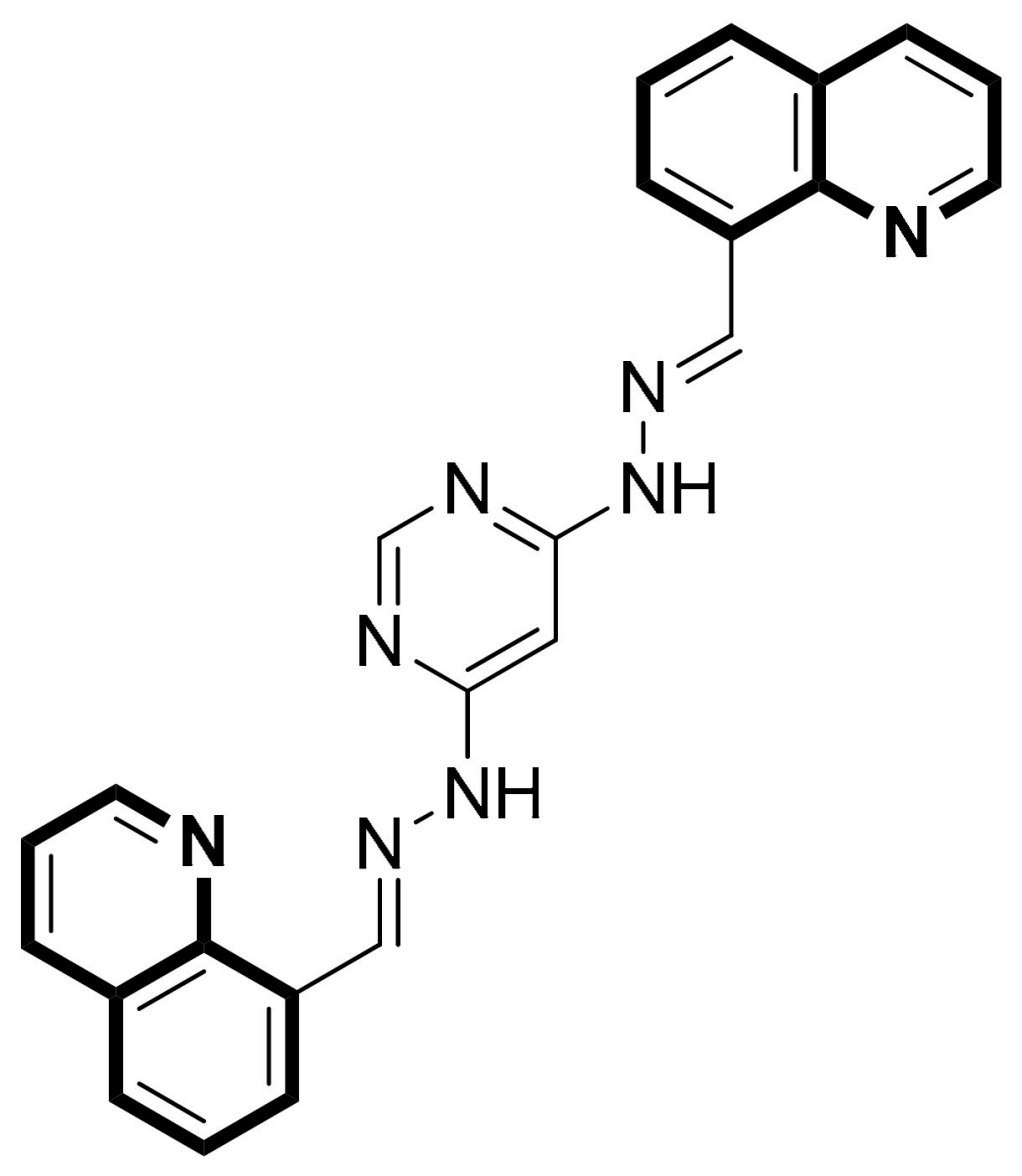	CDK2 In vitro: Breast adenocarcinoma MCF-7 cells (anti-proliferative activity and ROS/DNA binding evaluation) In silico: Molecular docking into the CDK2 ATP-binding domain to predict binding affinity, H-bonding interactions, and kinase inhibitory potential	**23a**	[129]
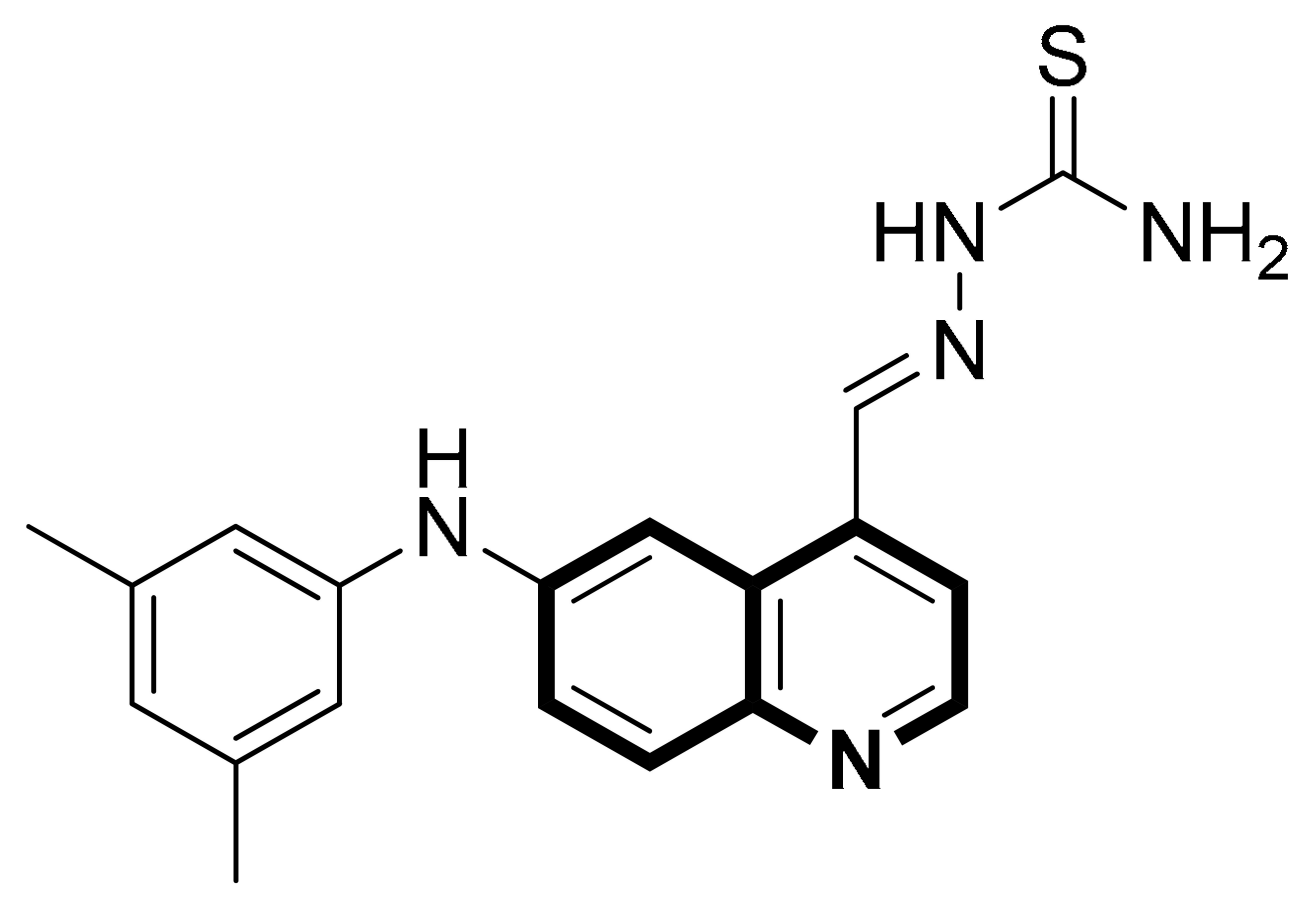	GAK In vitro: Ewing’s sarcoma cell models (nanomolar enzymatic GAK/NAK kinase inhibition and antiproliferative/antitumor activity)	**24**	[133]
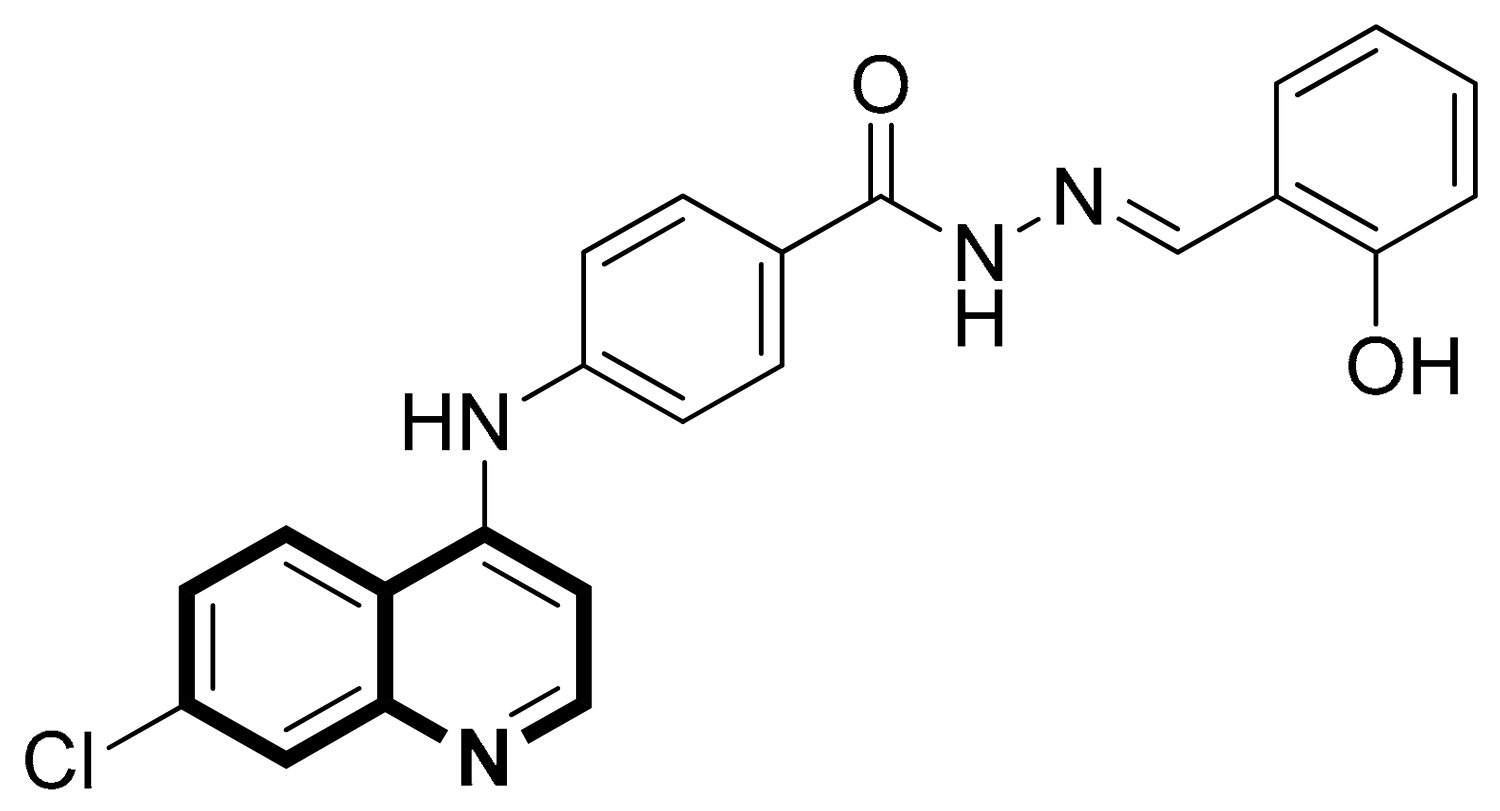	Eg5 protein (KSP) In vitro: TNBC MDA-MB-231 cells (inhibition of steady-state ATPase activity and cellular antiproliferative effect) In silico: Allosteric molecular docking, molecular dynamics simulations, Principal Component Analysis (PCA), and Free Energy Landscape (FEL) mapping	**25**	[139]
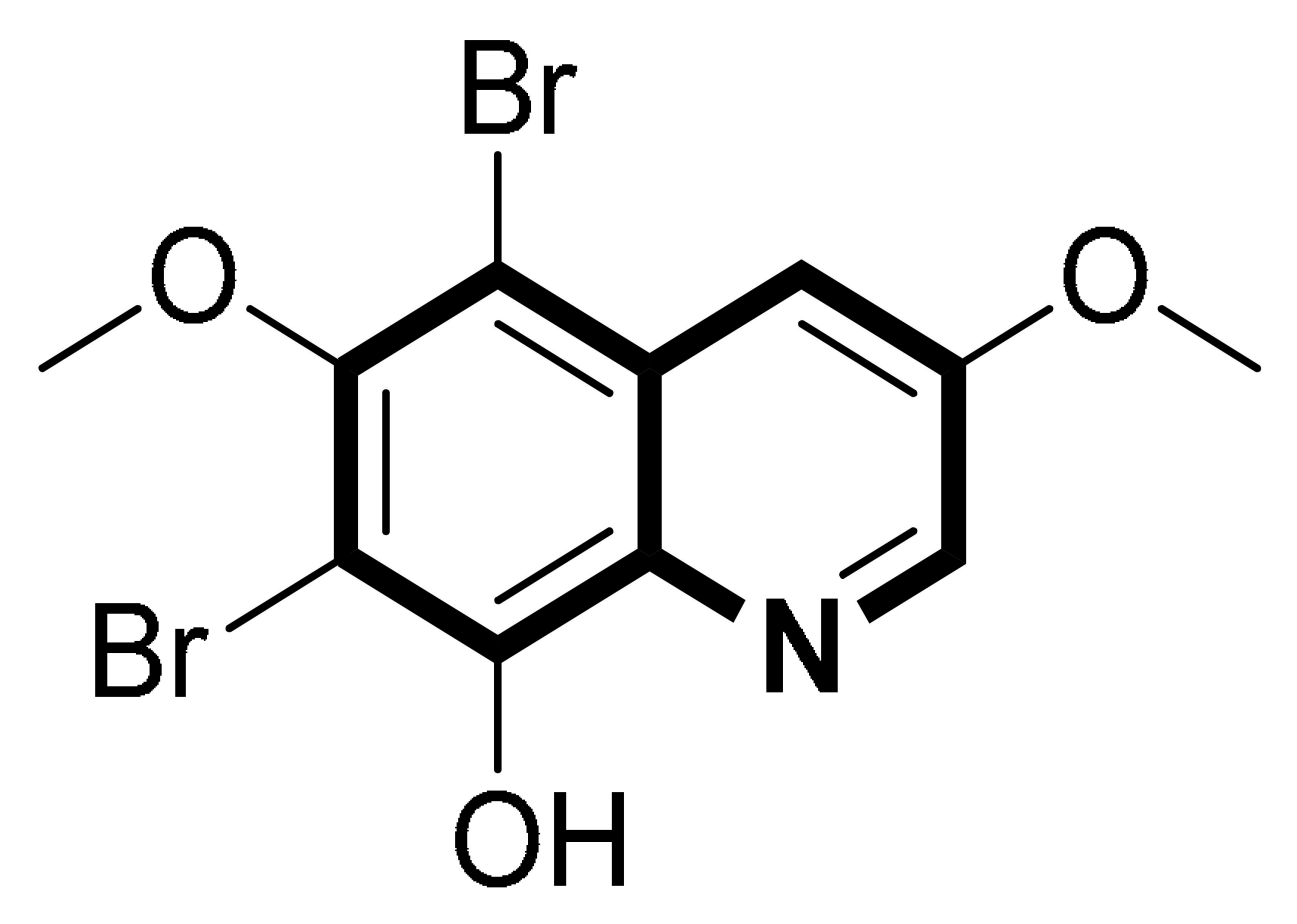	Topo-I In vitro: Rat glioblastoma C6, human cervical cancer HeLa, and colorectal adenocarcinoma HT29 cells (BCPE antiproliferative, LDH cytotoxicity, and wound-healing migration assays) In silico: Long-trajectory molecular dynamics (MD) simulations and MM-PBSA binding free energy calculations on the Topo-I–DNA adduct	**27b**	[142]
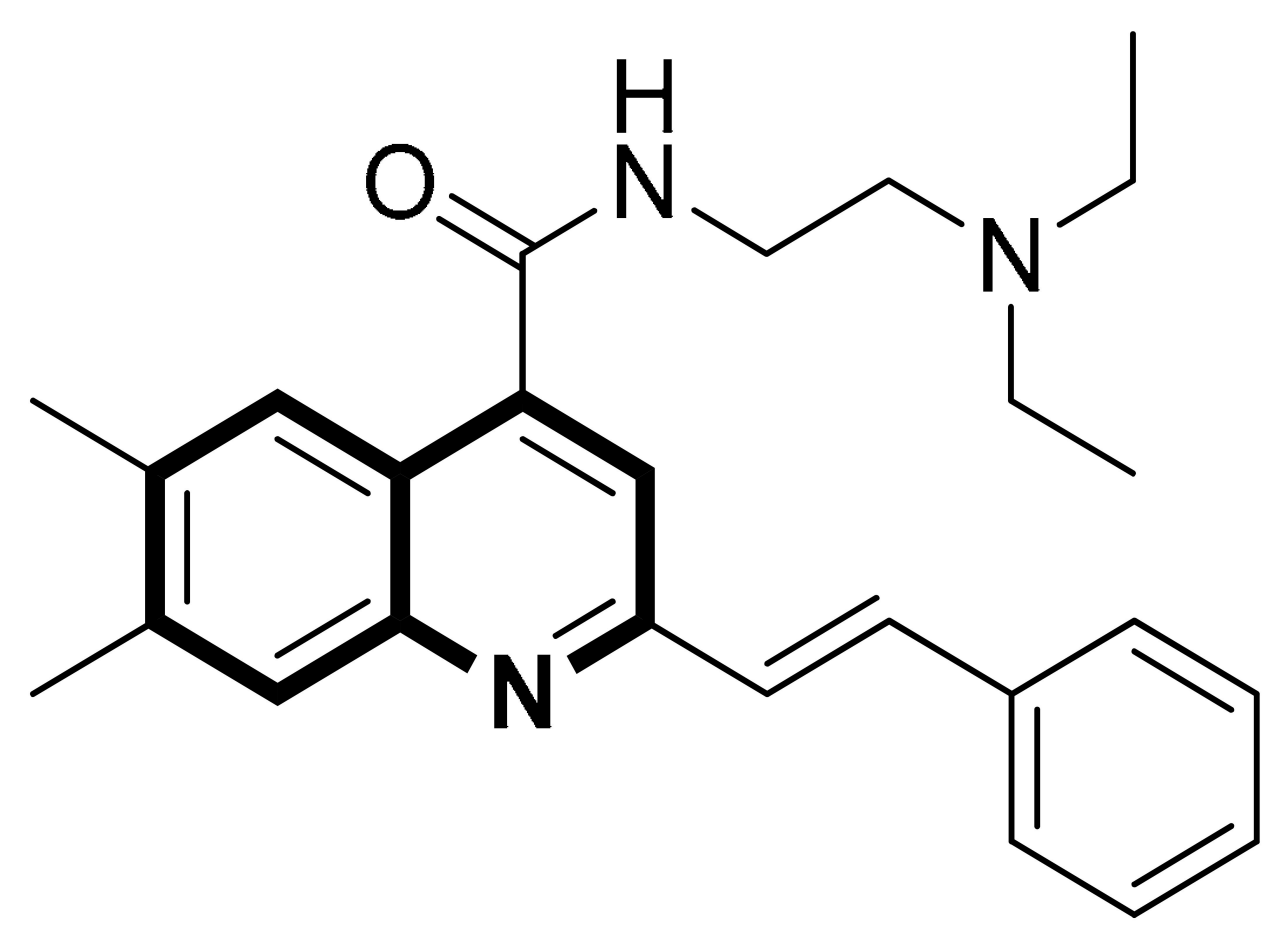	DNA intercalation In vitro: Lung adenocarcinoma A549 cell line (cytotoxicity) and ct-DNA multi-spectroscopic studies (UV-Vis, fluorescence competitive binding, CD spectroscopy, viscosity, and thermal melting assays) In silico: Molecular docking against DNA base pair double-helix to analyze intercalative insertion mode	**29**	[145]
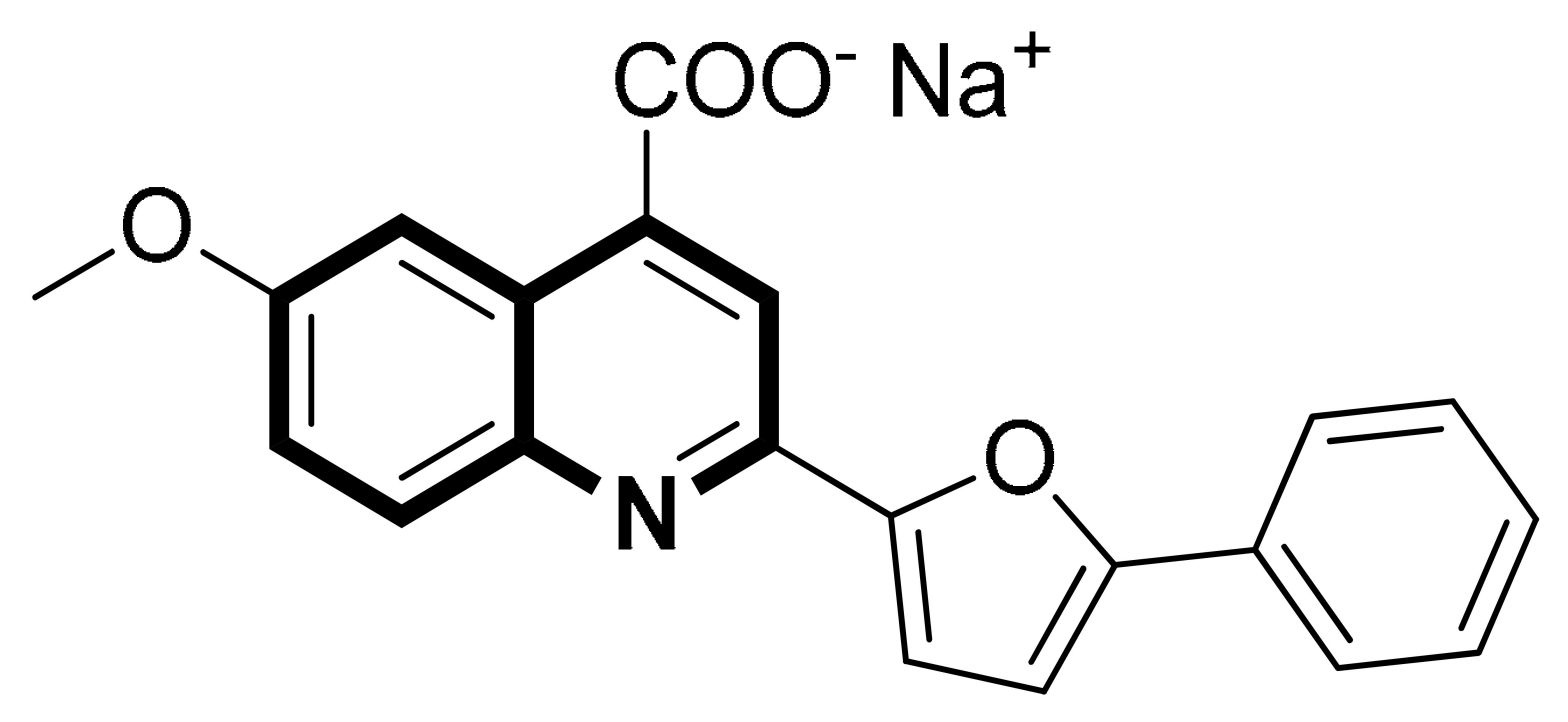	RAD51-BRCA2 interaction In vitro: Human pancreatic cancer cell models (BxPC-3; inhibition of RAD51–BRCA2 protein–protein interaction, disruption of homologous recombination repair, and enhancement of synthetic lethality with olaparib)	**31**	[148]
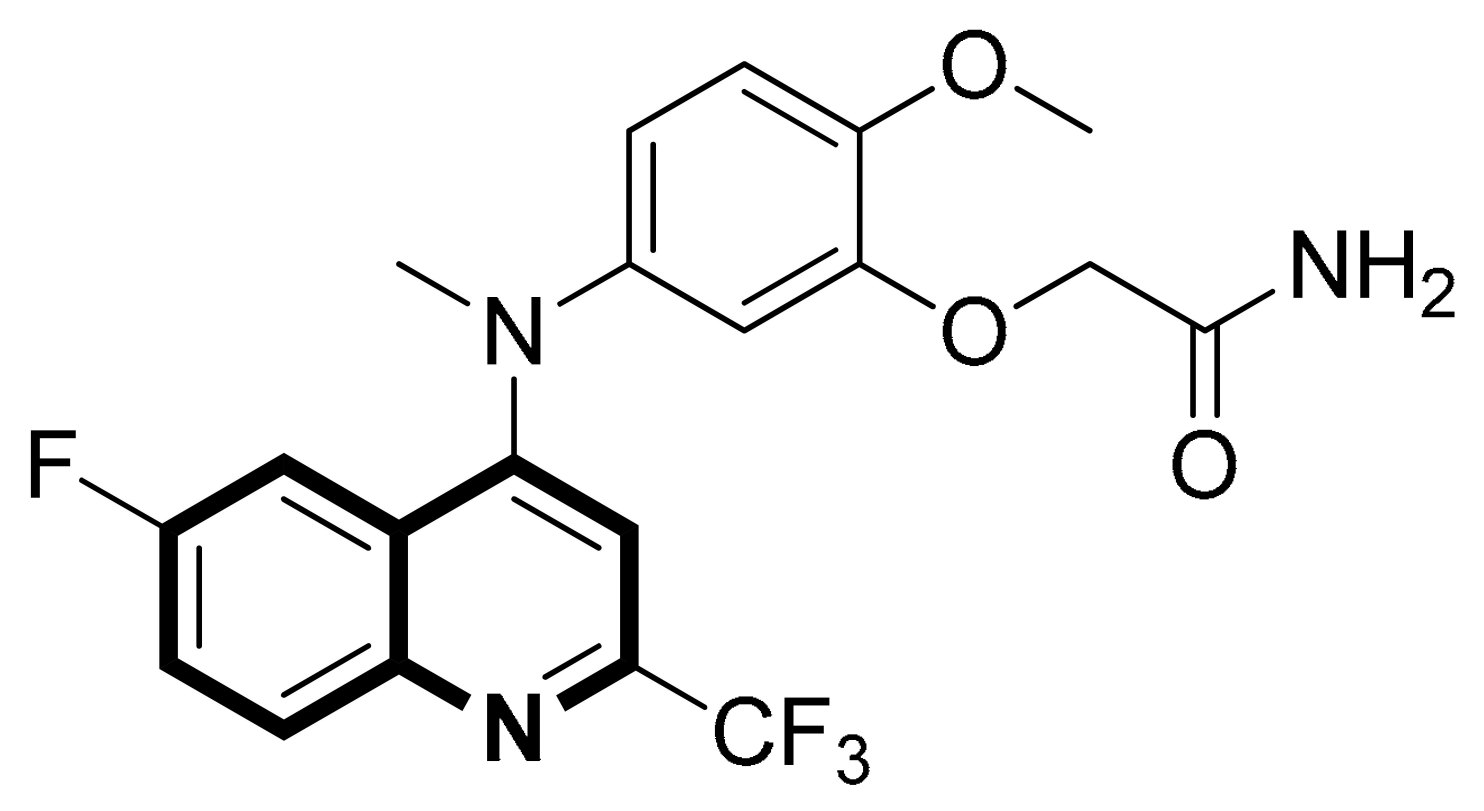	Tubulin (colchicine binding site) In vitro: Human prostate adenocarcinoma LNCaP cells (inhibition of tubulin polymerization, microtubule disruption, G2/M cell cycle arrest, and apoptosis induction)	**32**	[152]
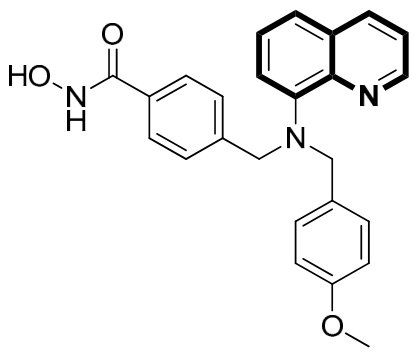	HDAC6 In vitro: Cervical cancer HeLa, non-small cell lung carcinoma A549, colorectal carcinoma HCT116, triple-negative breast cancer MDA-MB-231 and MDA-MB-468, acute T-cell leukemia Jurkat, multiple myeloma RPMI-8226, and acute lymphoblastic leukemia Molt-4 cells (enzymatic HDAC6 inhibition, substrate hyperacetylation, and antiproliferative/apoptotic profiling)	**33**	[155]
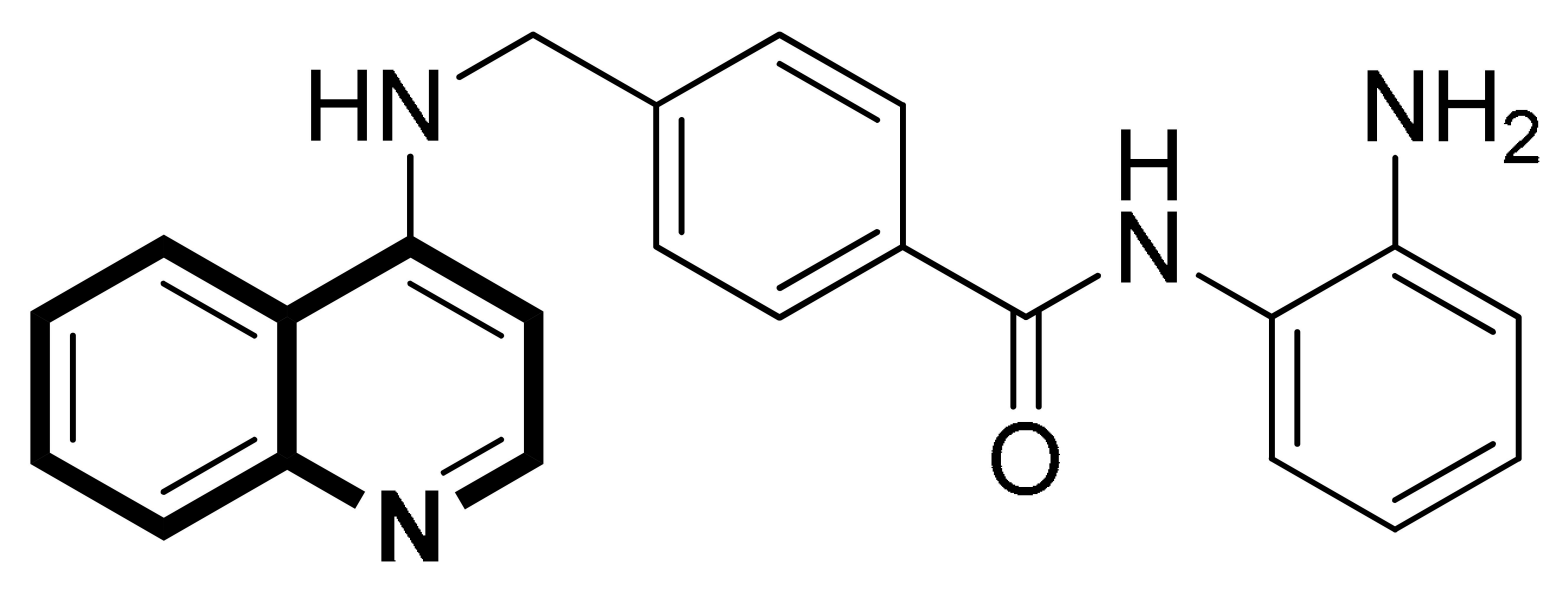	HDAC1 In vitro: Human promyelocytic leukemia HL-60, cutaneous T-cell lymphoma CCRF-CEM, and hepatoblastoma HepG2 cell lines (enzymatic HDAC1 inhibition and antiproliferative profiling) In vivo: Tumor xenograft efficacy studies (tumor growth inhibition) combined with full pharmacokinetic (PK) profiling (half-life, clearance, metabolic stability, and bioavailability)	**34**	[156]
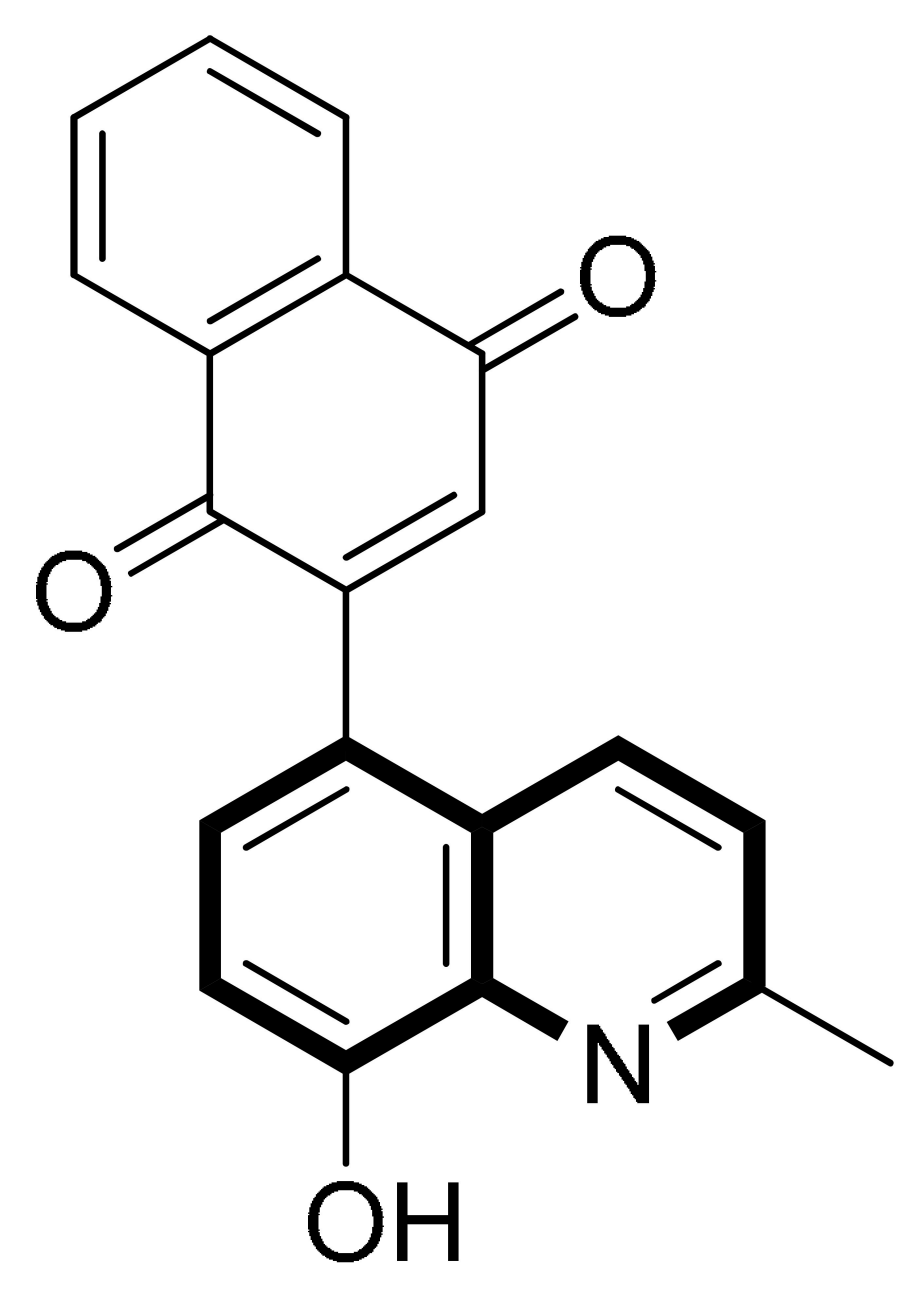	NQO1 In vitro: Lung carcinoma A549, breast adenocarcinoma MCF-7, and metastatic melanoma Colo-829 cell lines (cytotoxicity, enzymatic reduction assay, and mitochondrial apoptosis induction) In silico: Molecular docking inside the NQO1 active pocket to map FAD cofactor interactions and binding affinity	**37b**	[168]
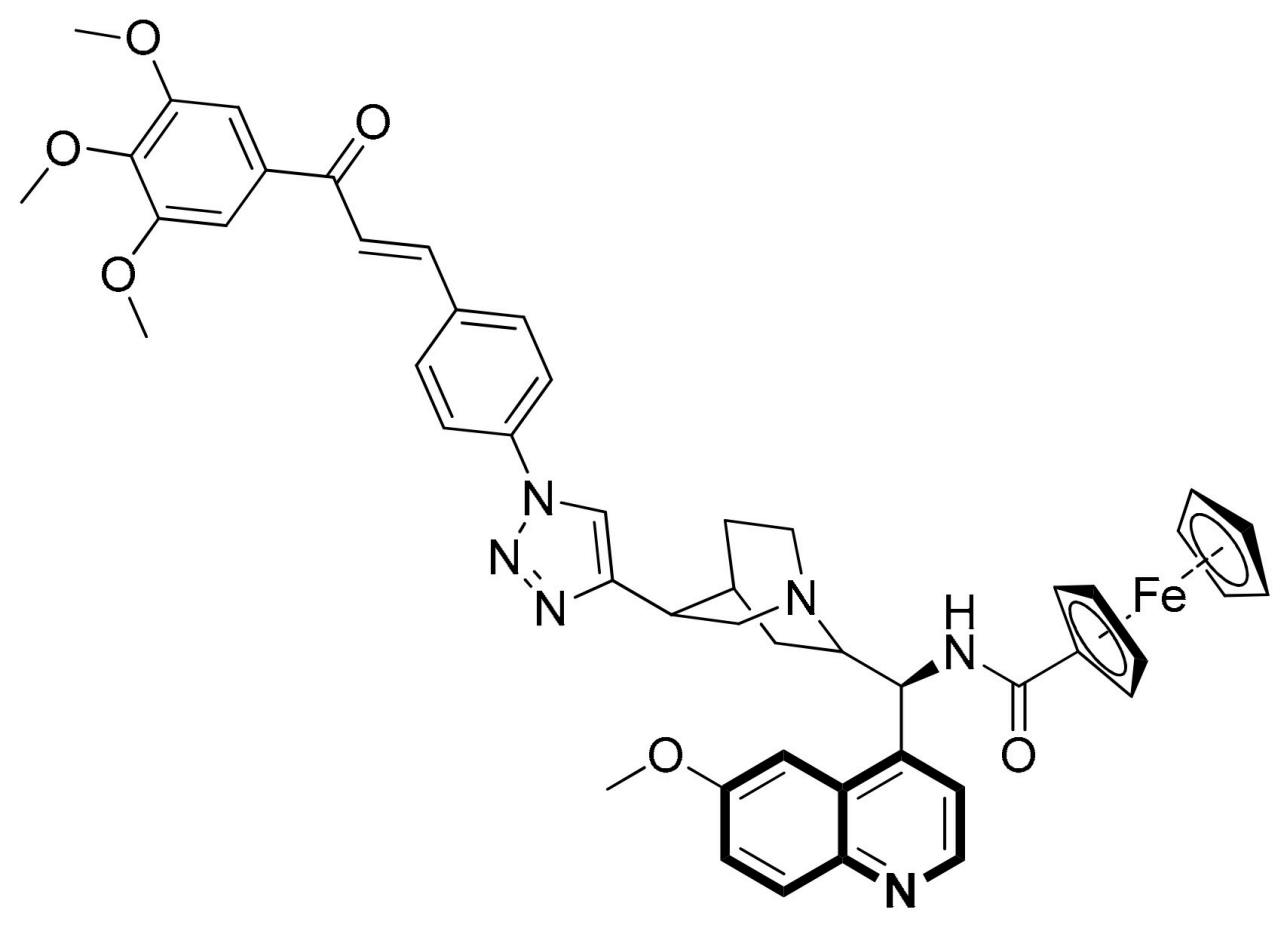	P-gp, caspase-8, and caspases-3/7 In vitro: Human lung squamous cell carcinoma EBC-1 cells (P-gp efflux inhibition to overcome multi-drug resistance, and activation of the extrinsic caspase-8/caspase-3/7 apoptotic cascade)	**38a**	[169]
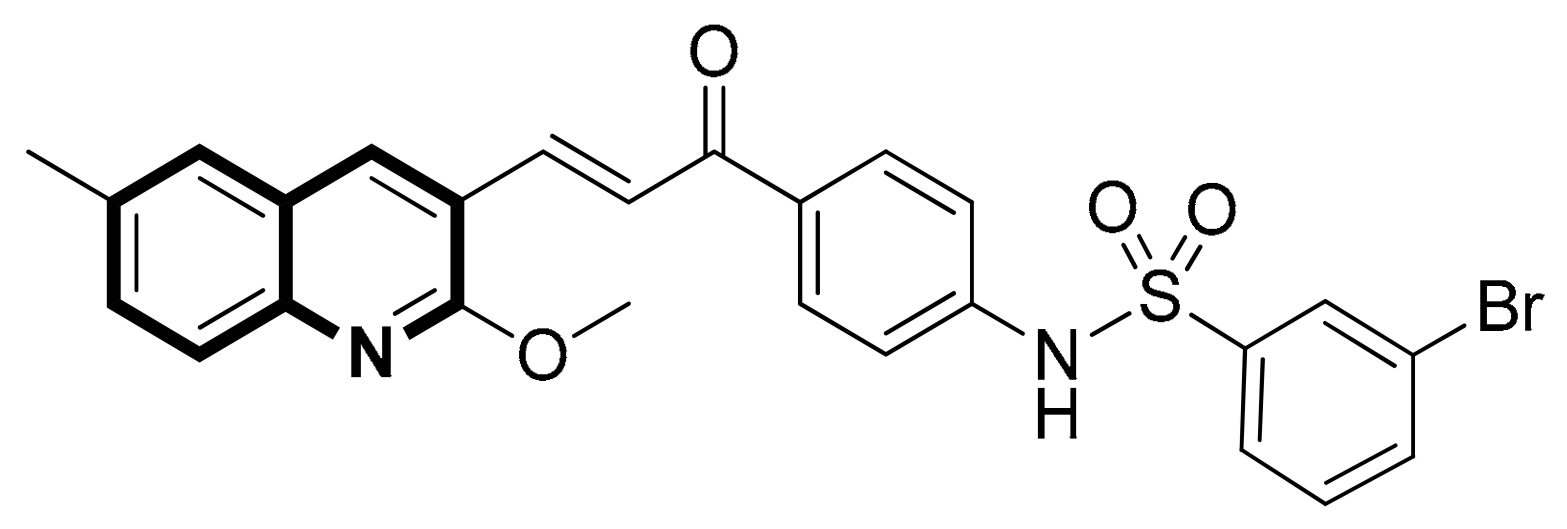	Bcl-2 protein In vitro: Human colon carcinoma HCT116, hepatoblastoma HepG2, and gastric adenocarcinoma AGS cell lines (cytotoxicity, Bcl-2 downregulation, mitochondrial membrane depolarization, and apoptosis induction)	**42b**	[177]

**Table 2:** Examples of valuable metal complexes of quinoline-based compounds with anticancer activity and their molecular targets, with study type and biological model.

Structure	Molecular Target/ Study Type/Biological Model	Number	Ref.
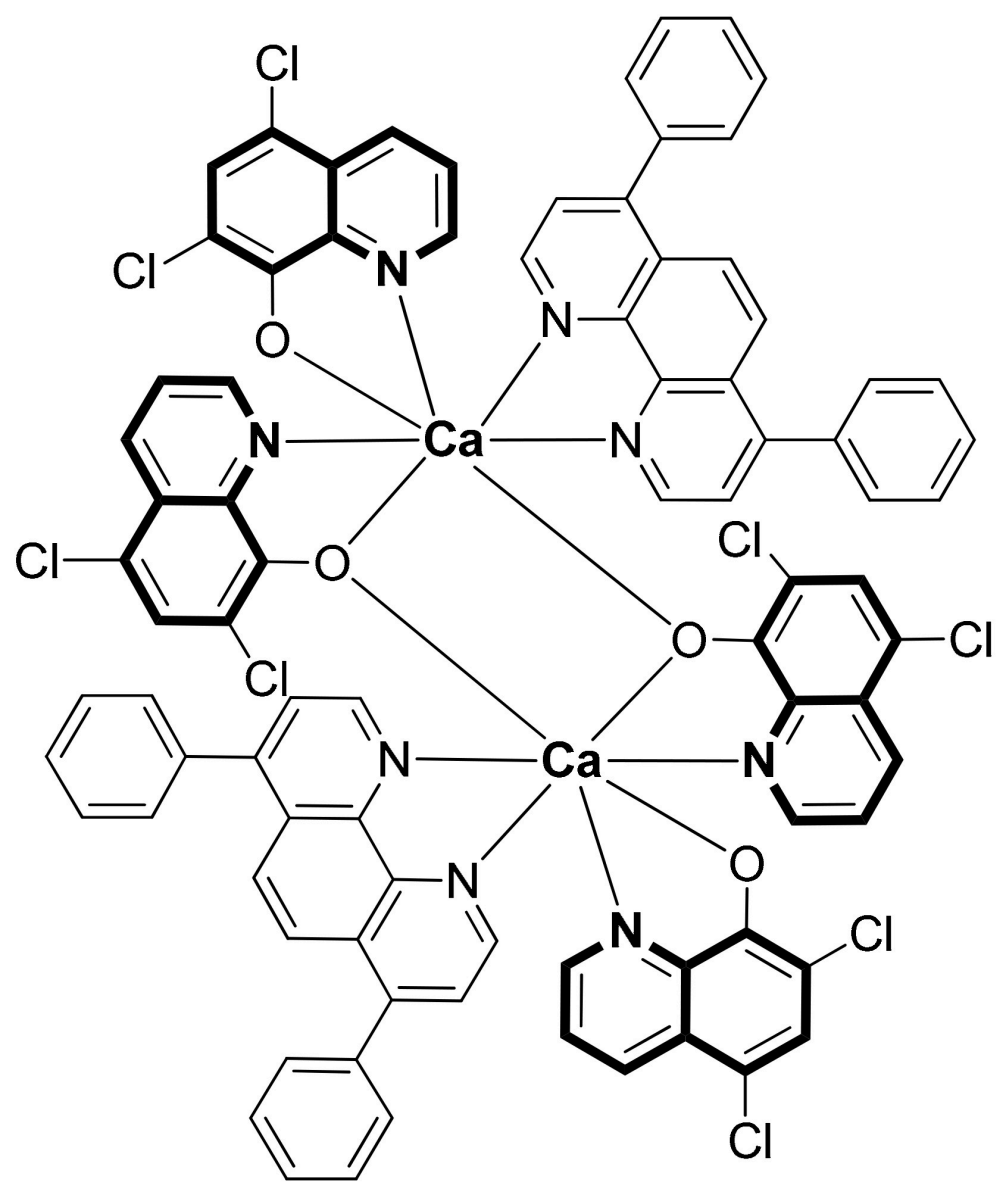	ROS LC3B II/I ration, Beclin1, PINK1, and Parkin protein expression In vitro: Human cisplatin-resistant ovarian cancer SK-OV-3/DDP (cis-SK3) cells (cytotoxicity, ROS generation, and mitophagy pathway regulation involving LC3B II/I, Beclin1, PINK1, and Parkin protein expression)	**43b**	[186]
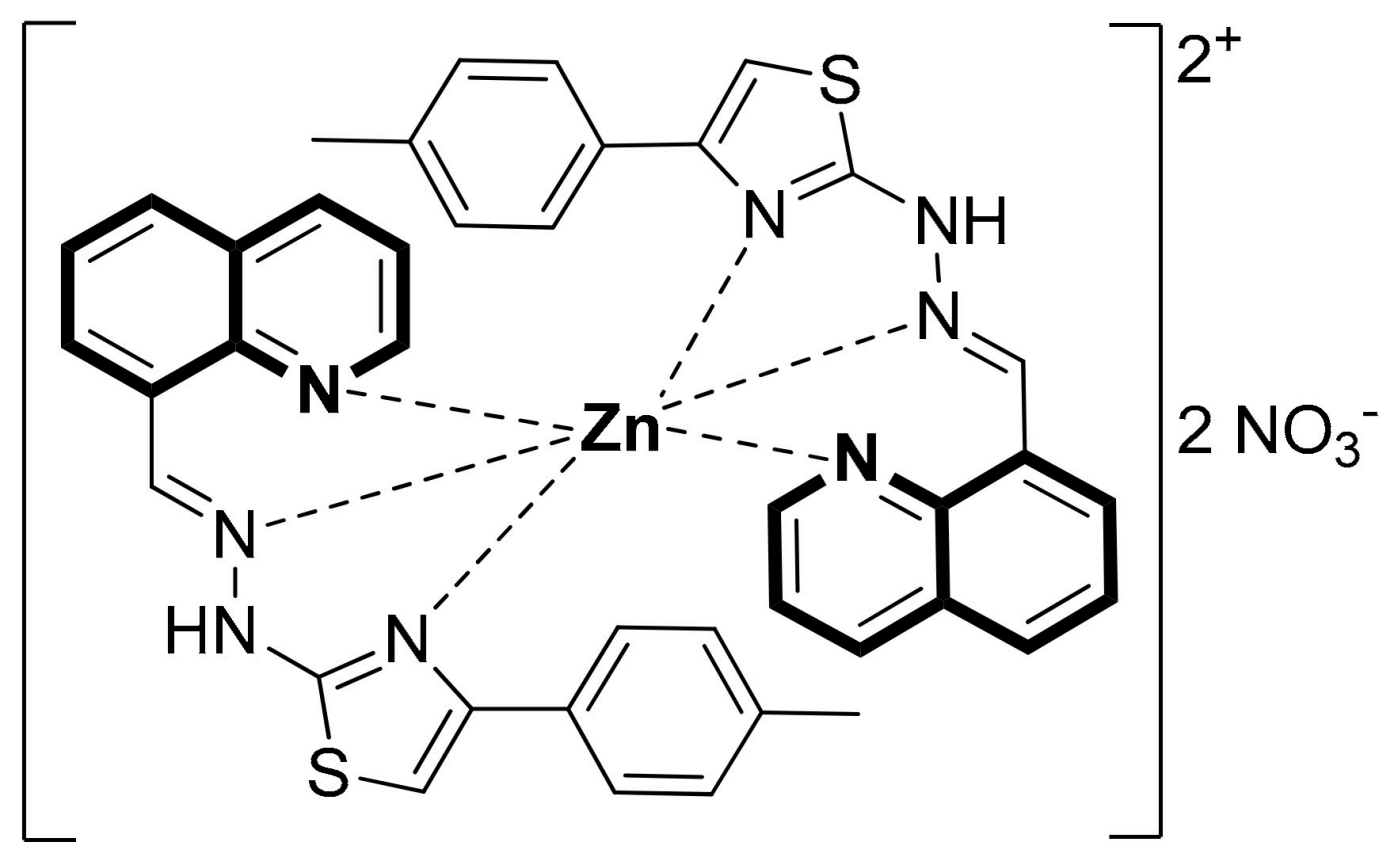	Caspase-3/7 (apoptosis activation) Mitochondrial membrane potential (ΔΨm) In vitro: Human lung adenocarcinoma A-549 and colorectal carcinoma HCT-116 cells (cytotoxicity, S-phase cell cycle arrest, inhibition of DNA synthesis, DNA damage indicated by increased p-*γ*H2AX expression, mitochondrial depolarisation, and intrinsic apoptosis induction) In vivo: Chick chorioallantoic membrane (CAM) tumor model (evaluation of tumor growth inhibition and antitumor activity).	**46**	[192]
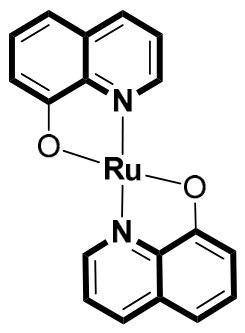	pro-apoptotic BAX (*BAX* gene) Aurora kinase B (*AURKB* gene) In vitro: Human estrogen receptor-positive breast cancer T47D and triple-negative breast cancer MDA-MB-231 cells (cytotoxicity, apoptosis induction, increased BAX protein expression and downregulation of AURKB gene expression)	**47**	[214]
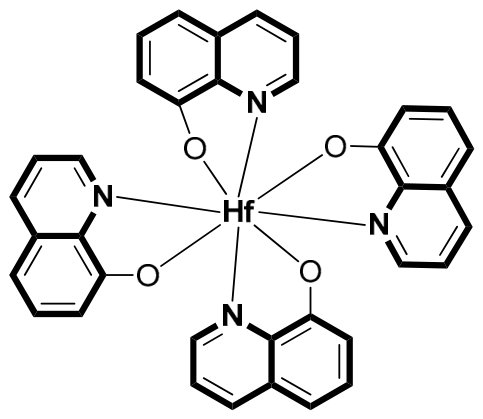	ROS BAX, BCL-2, and caspase-3 proteins In vitro: Human hepatoblastoma carcinoma HepG2, cervical adenocarcinoma HeLa S3, and lung cancer PC9 cell lines (cytotoxicity); ROS generation and mitochondrial dysfunction in Hep G2 cells.	**49a**	[216]

## Data Availability

No new data were created or analyzed in this study. Data sharing is not applicable to this article.
